# Identifying optimal ALK inhibitors in first- and second-line treatment of patients with advanced ALK-positive non-small-cell lung cancer: a systematic review and network meta-analysis

**DOI:** 10.1186/s12885-024-11916-4

**Published:** 2024-02-08

**Authors:** Mingye Zhao, Taihang Shao, Hanqiao Shao, Caicun Zhou, Wenxi Tang

**Affiliations:** 1 Department of Pharmacoeconomics, School of International Pharmaceutical Business, China Pharmaceutical University, Nanjing, Jiangsu China; 2 Center for Pharmacoeconomics and Outcomes Research, China Pharmaceutical University, Nanjing, Jiangsu China; 3 Shanghai Pulmonary Hospital, Tongji University, Shanghai, China

**Keywords:** ALK-inhibitors, Network meta-analysis, Non-small-cell lung cancer, Efficacy, Safety, Quality of life

## Abstract

**Objectives:**

To compare the efficacy, safety and effects on quality of life of different ALK-inhibitors for global and Asian patients with advanced ALK-positive non-small-cell lung cancer (NSCLC).

**Methods:**

The included RCTs were identified through a systematic search of PubMed, EMBASE, Cochrane Library, Clinical Trials.gov, and major cancer conferences. The assessment of progression-free survival (PFS), intracranial PFS, overall survival (OS), and patient-reported outcomes (PROs) was carried out using restricted mean survival time (RMST) model, fractional polynomial model and Royston-Parmar model. Time-invariant hazard ratio (HR) models were also used to validate and supplement the primary analysis. Objective response rate (ORR) and adverse events with any grade, grade 3–5 were assessed through a Bayesian network meta-analysis. The primary measures for OS, PFS, and PROs were HR and RMST. The odds ratio was the metric for evaluating safety, ORR, 12-month PFS rate, 24-month OS rate, and the 12-month non-deterioration rate of PROs. Subgroup analyses based on patient characteristics were performed.

**Results:**

A total of fourteen studies (ten for first-line, four for second-line) consisting of nine treatments (chemotherapy, crizotinib, alectinib [600mg BID], low-dose alectinib [300mg BID], brigatinib, ceritinib, ensartinib, envonalkib, and lorlatinib) were included. In the first-line setting, alectinib showed a significant advantage over crizotinib and had the longest OS among all ALK-inhibitors. Compared to crizotinib, lorlatinib had the best efficacy regarding PFS for global patients, followed closely by alectinib and brigatinib. For Asian patients, alectinib significantly improved PFS compared to other treatments. In second-line, alectinib had the highest PFS for patients pretreated with crizotinib, followed by brigatinib, ceritinib and chemotherapy. Alectinib, irrespective of the dose, was the safest first-line option, whereas lorlatinib, brigatinib, and ceritinib showed poorer safety profiles. Alectinib was also the safest ALK-inhibitor for crizotinib-resistant patients. Brigatinib had the best performance in terms of PROs.

**Conclusions:**

Considering both efficacy and safety, alectinib appears to be the preferable treatment in first-line and second-line, particularly for Asian patients.

**Supplementary Information:**

The online version contains supplementary material available at 10.1186/s12885-024-11916-4.

## Introduction

Lung cancer is the leading cause of new cancer-related deaths, representing 18% of all deaths caused by cancer [1]. Among all types of lung cancer, non-small cell lung cancer (NSCLC) accounts for approximately 80–85%, and 5-year survival rates for NSCLC are estimated to be 6.1% for those diagnosed between 2009 and 2015 [2], and anaplastic lymphoma kinase (ALK) rearrangements occur in approximately 5–8% of patients [3]. Advances in the development of targeted therapies significantly extended survival outcomes in NSCLC, especially for ALK-positive patients [4].

As of December 2022, seven ALK-tyrosine kinase inhibitors have been evaluated in Phase III randomized controlled clinical trials (RCT) [5–17]. Crizotinib, as the first-generation ALK-inhibitors, was the initial to receive approval from the US Food and Drug Administration. Compared to chemotherapy, it reportedly provided patients with considerably progression-free survival (PFS) benefits. Furthermore, further generations of ALK-inhibitors, including second-generation ceritinib, brigatinib, ensartinib, alectinib, envonalkib, and third-generation lorlatinib, displayed notably extended survival outcomes in comparison to crizotinib [6, 8–10, 13, 14, 16, 18, 19]. Sequential treatment with ALK-inhibitors has demonstrated increased survival benefits for patients who are resistant to crizotinib or chemotherapy [5, 6, 11, 12, 20].

Despite several systemic treatments being available for ALK-positive NSCLC, the comparative efficacy and safety of most first- or second-line treatments are still unknown considering the lack of clinical trials, and most of related phase III RCTs firstly reporting or updating survival data recently [5, 6, 10, 20–27]. This means that it is necessary to conduct indirect comparisons to compare the relative efficacy of these ALK-inhibitors. However, the existing network meta-analyses gathered limited clinical evidence and overlooked some important indicators, and utilized proportional hazards (PH) model to compare the efficacy without testing for PH assumption. This implies a pressing need for more refined evidence.

Therefore, we conducted a comprehensive network meta-analysis (NMA) using additional data and methodological approaches to compare the safety and efficacy of current therapies in first- or second-line treatment of ALK-positive NSCLC patients across various subgroups, and to evaluate the impacts of these therapies on patients’ quality of life (QoL).

## Methods

### Protocol

Our study was conducted in accordance with the Preferred Reporting Items for Systematic Reviews and Meta-Analyses extension statement for network meta-analyses of healthcare interventions (PRISMA) [28]. See Additional file 1 for the study checklist and Additional file 2 for the study protocol (CRD42021288638).

### Search strategy

The search strategy is provided in Additional file 3. Briefly, as of December 2022, two researchers (Zhao and Shao) systematically searched PubMed, EMBASE, Cochrane Library, and ClinicalTrials.gov for related clinical trials and published studies on associated drugs. The keywords "ALK", "Anaplastic Lymphoma Kinase", "non-small-cell lung cancer", "NSCLC", "randomized controlled trials", and "RCT" were used in the search. There is no limitation on publication date or language. Additionally, we searched abstracts from the European Society for Medical Oncology, American Society of Clinical Oncology, and World Conference on Lung Cancer.

### Selection criteria

The titles and abstracts of the included articles were initially screened by two researchers, Zhao and Shao. In case of any disagreement, a discussion was held among a panel of authors, which included an expert in oncology. The eligibility criteria, as per the PICOS framework, were as follows:

1. Population: Adult patients with histologically or cytologically confirmed ALK-positive NSCLC including those in advanced stages, no restrictions were placed on individual-level characteristics.
2. Interventions and comparisons: Reasonable systematic interventions, including pharmaceutical, surgical, radiological, and combination therapies, were evaluated.
3. Outcomes: The primary outcomes investigated in this study were systemic and intracranial PFS, overall survival (OS). Subgroup analysis was performed considering variables such as region, presence of brain metastasis (BM), Eastern Cooperative Oncology Group (ECOG) performance status, gender, age, and smoking status. Objective response rate (ORR), adverse events (AEs, [any grade, grade 3–4, grade 5 or fatal]) and patient-reported outcomes (PROs) were secondary outcomes for our study, PROs including European Organisation for Research and Treatment of Cancer (EORTC) Quality of Life Questionnaire: Lung Cancer Module (QLQ-LC13) composite score (dyspnea, coughing and pain in chest), European 0ranization for Research and Treatment of Cancer QoL questionnaire-Core 30 (QLQ-C30) global health status.
4. Study design: Phase III RCTs with intended data were primarily considered. We only incorporated trials with the most recent and informative data to avoid duplication. If eligible studies lacked the same kind of updated data, data reported previously could be used.

### Data extraction

Required data was extracted by two independent researchers. The extracted information included characteristics of eligible trials (publication year, first author, registration information, etc.), characteristics of populations (age, sample size, countries, etc.), and characteristics of program (interventions, outcomes of endpoints, etc.). Independent review committee (IRC) data were considered. For trials where data or updated data was unavailable from IRC, we utilized the investigator-assessed results as the primary analysis [5, 24, 29], and results based on IRC-data with shorter follow-up were used as a validation analysis [8, 9, 18]. Modified intent-to-treat population-based data were considered as subgroup data based on intent-to-treat population were unavailable in eXalt3 [13].

### Quality assessment

Quality of the included studies was evaluated by modified Cochrane Collaboration’s risk of bias (ROB) tool [30]. The quality of eligible studies was categorized as high, low, or unclear. The Egger regression test was conducted to determine publication bias, with p-values of less than 0.05 indicating publication bias. Two researchers independently assessed the quality of included RCTs. In case of any discrepancies, a final consensus was reached through discussions.

### Statistical analyses

We ran both fixed- and random-effects models, with the latter taking between-study heterogeneity into account. Out of the 14 treatment comparisons analyzed through RCTs, 12 were studied only in one trial. The consistent results from both fixed- and random-effects models led us to report the findings from fixed-effects consistency models. PFS, OS and ORR were used to evaluate efficacy, QLQ-LC13 composite score and QLQ-C30 global health status were used to evaluate PROs, and AEs were used as safety indicators.

For time-to-event data, the hazards ratios (HRs) between treatments were frequently found to be time-varying, and clear violations of the PH assumption were detected in studies such as ALEX, CROWN, and Profile 1014 [8, 12, 16] (See Additional file 4). Therefore, it would be inappropriate to solely use HRs based on Cox-PH models as measures of effect size in NMA. In response, we opted to utilize the restricted mean survival time (RMST) as a more comprehensive and appropriate short-term measure [31–34], the longest time used in RMST model depended on follow-up durations of RCTs. To rank the relative efficacy of different interventions, we calculated the mean ranking based on the probabilities of each rank obtained from Markov simulations. We estimated time-varying HRs and expected long-term survival rates using frequentist Fractional Polynomial (FP) models [35], we fitted a series of first-order FP models with power parameters of -2, -1, -0.5, 0.5, 1, 2, and 3. Model fit was assessed using the Akaike information criterion (AIC) [35]. FP models were performed by the “survival” package in R, version 4.1.0. To verify the results, Royston-Parmar (RP) models were also run under a Bayesian framework, considering the uncertainty of parametric models [36]. We utilized the RP model with three independent Markov chains running 5,000 burn-ins and 10,000 sample iterations per chain simultaneously, utilizing one step-size iteration in both Winbugs (version 1.4) and R. Survival rate and life-years gained was selected as measures in this part. Additionally, we digitalized published Kaplan–Meier curves via the GetData Graph Digitizer software version 2.24. To obtain individual patient data (IPD), we followed Guyot’s [37] method.

To conduct a more comprehensive analysis of studies lacking Kaplan–Meier curves and to conduct more subgroup analyses, we utilized Cox-PH models using the “Netmeta” package in R. This approach produced conservative outcomes and helped establish more complete networks. HRs with 95% credible intervals (CIs) were the measure for Cox-PH models.

Bayesian NMAs were conducted to evaluate AE, ORR, 12-month PFS rate, 24-month OS rate, and the non-deterioration rate of the 12-month QLQ-LC13. The analyses utilized the R 'BUGSnet' package and were carried out with 10,000 post-burn-in samples, derived from four parallel Markov chains, each preceded by a 1,000-sample burn-in period. Odds ratios (ORs) with 95% CIs were used as effect sizes. Heterogeneity between studies was assessed using the Cochran’s Q test and I^2^ statistic within a visual forest plot, I^2^ statistic > 50% or a *P* value < 0.1 for the Q test was considered as indicating significant heterogeneity [38], the inconsistency of models was evaluated using the edge-splitting method, which took into account all direct and indirect evidence [39, 40]. Convergence of Markov chains was verified by trace plots and Gelman-Rubin diagnostic statistics [41].

## Results

### Characteristics of the included studies

A total of 1110 records were identified from the databases mentioned above. Out of these, 945 records were initially excluded based on the selection criteria, 165 potentially relevant studies were identified for a detailed full-text review. Finally, after applying the eligibility criteria, 14 studies were included in our network. Further details are provided in Additional file 5, eFigure 1.

In total, 3474 ALK-positive NSCLC patients were included in this study. 10 RCTs focused on first-line treatments, while the remaining assessed second-line treatments for patients who had previously received chemotherapy, crizotinib, or both [5–18]. Nine first-line treatments were involved, comprising of chemotherapy (pemetrexed plus cisplatin or carboplatin), one first-generation ALK-inhibitor (crizotinib), five second-generation ALK-inhibitors (ceritinib, brigatinib, alectinib [600 mg bid, approved globally], low-dose alectinib [300 mg bid, only approved and utilized in Japan], and envonalkib), and one third-generation ALK-inhibitor (lorlatinib). Notably, the data for low-dose alectinib and envonalkib was specific to Asians [6, 7]. Second-line treatments included chemotherapy (pemetrexed or docetaxel), crizotinib (for chemotherapy-resistant patients), ceritinib, brigatinib and alectinib. Main characteristics are summarized in Table 1, more details are provided in Additional file 5, eTable 1.

**Table 1:** Baseline characteristics of included studies and patients

Trial information	Intervention	Total patients	Region	HR for OS (95% CI)	HR for PFS (95% CI)	ORR (95% CI, %)	Grade 3 + AE rate (%)
ALTA-1L [10, 26, 42]	Brigatinib VS Crizotinib	137 VS 138	Global	0.81 (0.53–1.22)	0.48 (0.35–0.66)^a^ 0.43 (0.31–0.58)^b^	71 (62–78) VS 60 (51–68)	61 VS 55
PROFILE 1014 [15, 43, 44]	Crizotinib VS Pemetrexed + Cisplatin/Carboplatin	172 VS 171	Global	0.76 (0.55–1.05)	0.45 (0.35–0.60)^a^	74 (67–81) VS 45 (37–53)	NA
CROWN [16, 22, 23, 27, 45]	Lorlatinib VS Crizotinib	149 VS 147	Global	0.72 (0.41–1.25)	0.27 (0.18–0.39)^a^ 0.19 (0.13–0.27)^b^	76 (68–83) VS 58 (49–66)	72 VS 56
ALEX [8, 29, 46–48]	Alectinib (600 mg twice daily) VS Crizotinib	152 VS 151	Global	0.67 (0.46–0.98)	0.67 (0.46–0.98)^a^ 0.43 (0.32–0.58)^b^	83 (76–89) VS 76 (68–82)	41 VS 50
eXalt3 [13, 21, 49]	Ensartinib VS Crizotinib	143 VS 147	Global	0.91 (0.54–1.54)	0.50 (0.36–0.71)^a^	74 (66–81) VS 67 (58–74)	NA
ASCEND-4 [14, 50]	Ceritinib VS Pemetrexed + Cisplatin/Carboplatin	189 VS 187	Global	0.73 (0.50–1.08)	0.55 (0.42–0.73)^a^ 0.49 (0.37–0.64)^b^	73 (66–79) VS 27 (21–34)	78 VS 62
PROFILE 1029 [17, 19]	Crizotinib VS Pemetrexed + Cisplatin/Carboplatin	104 VS 103	Asia	0.90 (0.56–1.44)	0.40 (0.29–0.57)^a^	88 (80–93) VS 46 (36–56)	NA
J-ALEX [7, 25]	Low-dose alectinib (300 mg twice daily) VS Crizotinib	103 VS 104	Asia	1.03 (0.67–1.58)	0.34 (0.17–0.71)^a^ 0.34 (0.21 -0.54)^b^	92 (86–98) VS 79 (71–87)	26 VS 52
ALESIA [9, 51]	Alectinib (600 mg twice daily) VS Crizotinib	125 VS 62	Asia	0.60 (0.37–0.99)	0.37 (0.22–0.61)^a^ 0.33 (0.23–0.49)n	91 VS 77	29 VS 48
TQ-B3139 [6]	Envonalkib VS Crizotinib	131 VS 133	Asia	NA	0.46 (0.32–0.66)^a^	82 VS 70	52 VS 41
ALUR [18, 24]	Alectinib (600 mg twice daily) VS Pemetrexeor Docetaxel	72 VS 35	Global	0.91 (0.49–1.70)	0.32 (0.17–0.59)^a^ 0.20 (0.12–0.33)^b^	27 (26–50) VS 1 (0–15)	27 VS 41
ASCEND-5 [11]	Ceritinib VS Pemetrexed or Docetaxel	115 VS 116	Global	1.0 (0.67–1.49)	0.49 (0.36–0.67)^a^	39 (30–49) VS 7 (3–13)	NA
ALTA-3 [5]	Brigatinib VS Alectinib (600 mg twice daily)	125 VS 123	Global	NA	0.97 (0.66–1.42)^a^ 1.23 (0.86–1.76)^b^	52 (43–61) VS 61 (52–70)	44 VS 18
Profile 1007 [12, 52]	Crizotinib VS Pemetrexed or Docetaxel	173 VS 174	Global	1.02 (0.68–1.54)	0.49 (0.37–0.64)^a^	65 (58 − 72) VS 20 (14 − 26)	56 VS 46

^a^Blind Independent Central Review data

^b^Investigator-assessed data

### Risk of Bias

The assessment of ROB is presented in Additional file 4. Overall, the ROB in all RCT studies was generally low. However, all included RCTs were open-label, which increased the ROB in the blinding of participants and personnel as well as allocation concealment. In addition, concerns were raised about potential bias in the ALTA-3 and TQ-B3139 due to incomplete outcome data [5, 6]. Besides, selective reporting raised concerns of bias in eXalt3 [13]. Egger regression test results suggested no publication bias in our network, the funnel plots are displayed in Additional file 4.

### Syntheses of results

HRs, life-years gain and differences in RMST (RMSD) of the treatments in the included trials and for each network are summarized in Additional file 6. Network plots for PFS, OS, PROs, ORR, and AEs are provided on Additional file 5, eFigure 2.

### Efficacy outcomes

#### Overall survival

**Fig. 1:**
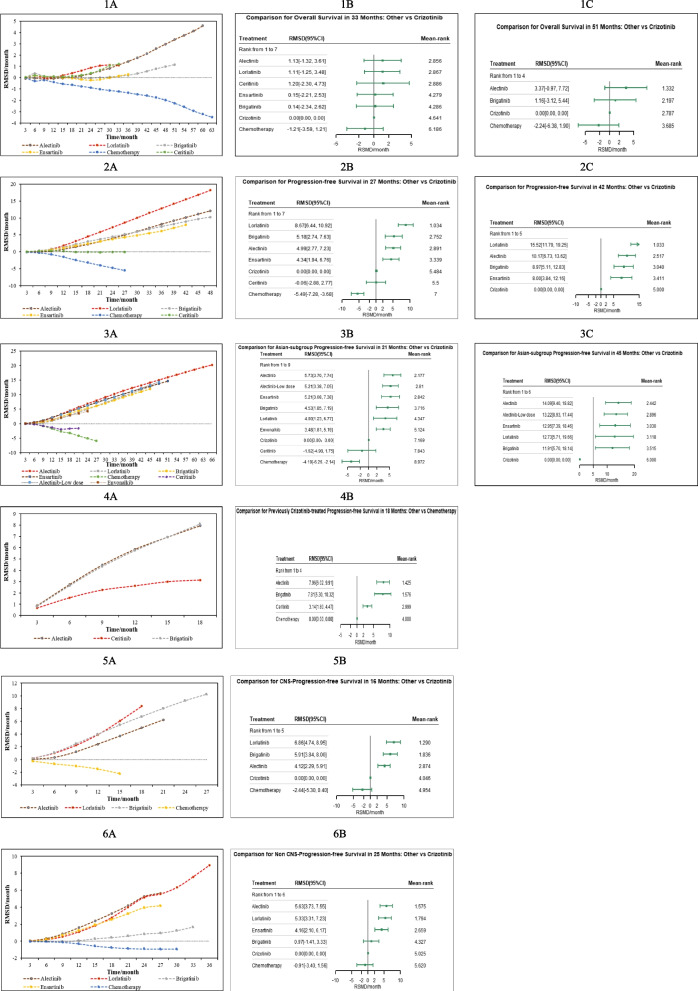
Summary Results of RMST (1A. RMSD of Treatments Compared with Crizotinib over Time for OS on global patients; 1B. Forest Plots of Treatments Compared with Crizotinib in 33 Months for OS on global patients; 1C. Forest Plots of Treatments Compared with Crizotinib in 51 Months for OS on global patients; 2A. RMSD of Treatments Compared with Crizotinib over Time for PFS on global patients; 2B. Forest Plots of Treatments Compared with Crizotinib in 27 Months for PFS on global patients; 2C. Forest Plots of Treatments Compared with Crizotinib in 42 Months for PFS on global patients; 3A. Asian-subgroup RMSD of Treatments Compared with Crizotinib over Time for PFS; 3B. Asian-subgroup Forest Plots of Treatments Compared with Crizotinib in 21 Months for PFS; 3C. Asian-subgroup Forest Plots of Treatments Compared with Crizotinib in 45 Months for PFS; 4A. RMSD of Second-line Treatments Compared with Chemotherapy over Time for PFS; 4B. RMSD of Second-line Treatments Compared with Chemotherapy in 18 Months for PFS; 5A. RMSD of Treatments Compared with Crizotinib over Time of intracranial PFS for Baseline Brain Metastasis Patients; 5B. RMSD of Treatments Compared with Crizotinib in 16 Months of intracranial PFS for Baseline Brain Metastasis Patients; 6A. RMSD of Treatments Compared with Crizotinib over Time of intracranial PFS for Baseline No Brain Metastasis Patients; 6B. RMSD of Treatments Compared with Crizotinib in 16 Months of intracranial PFS for Baseline No Brain Metastasis Patients)

**Fig. 2:**
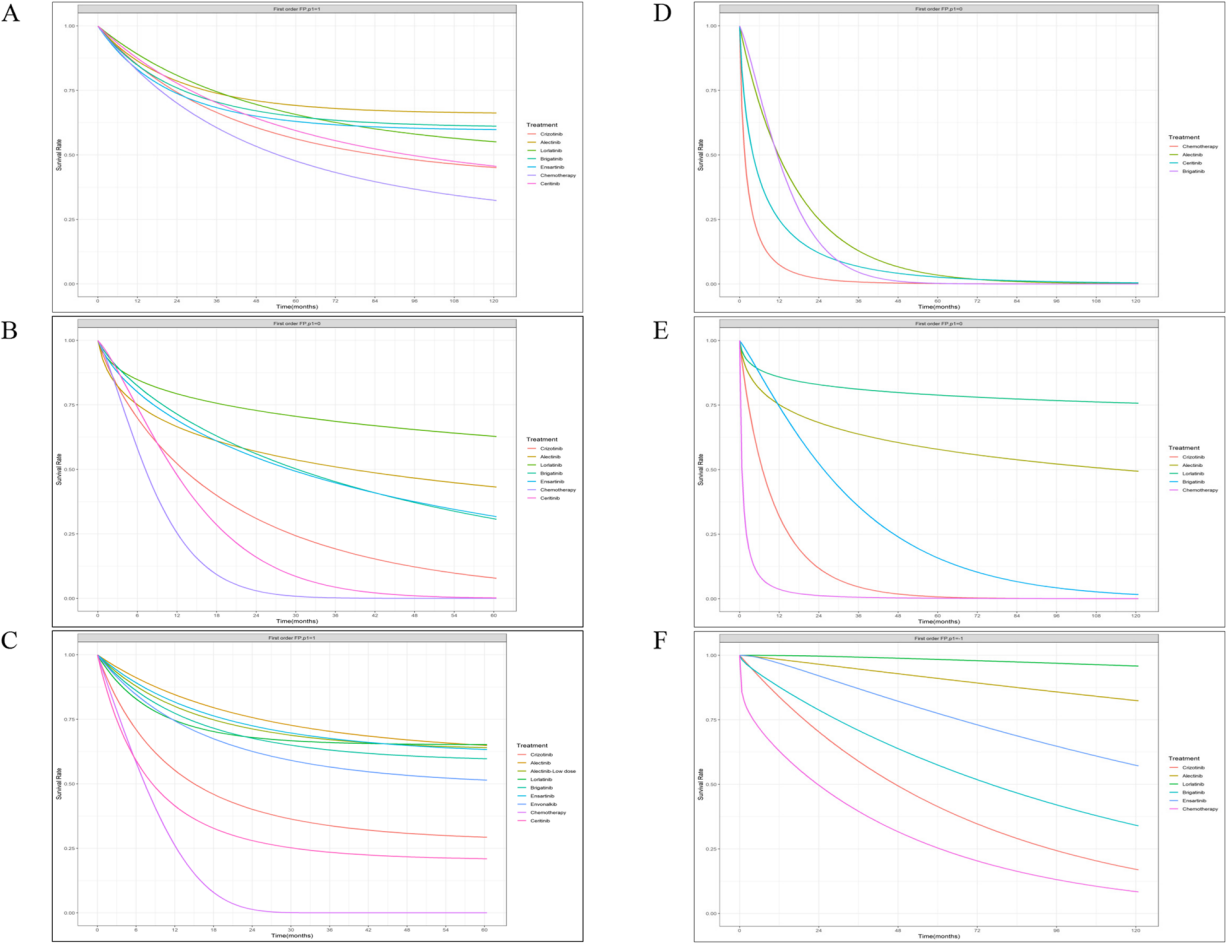
Survival Curves based on FP models (**A** OS of First-line Treatments on global patients; **B** PFS of First-line Treatments on global patients; **C** PFS of First-line Treatments on Asian Patients; **D** PFS of Second-line Treatments; **E** intracranial PFS of First-line Treatments for Baseline BM Patients; **F** intracranial PFS of First-line Treatments for Baseline No BM Patients)

**Fig. 3:**
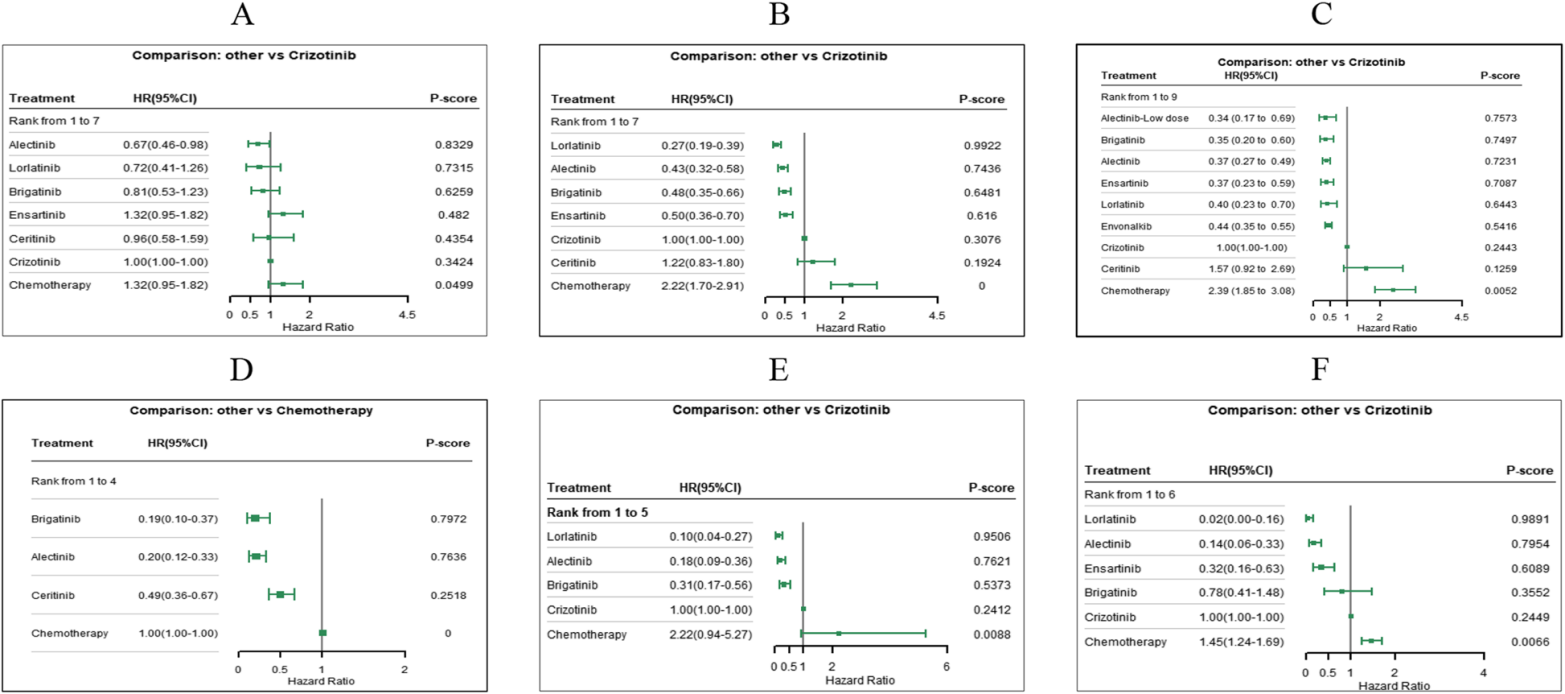
Forest Plots for Cox-PH Model (**A** OS of First-line Treatments on Global patients; **B** PFS of First-line Treatments on Global patients; **C** PFS of First-line Treatments on Asian Patients; **D** PFS of Second-line Treatments; **E** intracranial PFS of First-line Treatments for Baseline Brain Metastasis Patients; **F** intracranial PFS of First-line Treatments for Baseline No Brain Metastasis Patients)

Evidence from RMST models indicated that compared with crizotinib until 33 months (the shortest follow-up time of all included trials), alectinib had the highest mean rank of improved OS (RSMD, 1.13 months [95% CI, -1.32 ~ 3.61]). No significant difference was existed among alectinib, lorlatinib and ceritinib, and these three drugs were significantly better than other four treatments. Detailed information is presented in Fig. 1 1A-1B and Additional file 5, eFigure 3. Specifically, the FP model with power parameter (P) = 1 fit the data best. As shown in Fig. 2A, alectinib had the highest OS rate, followed by lorlatinib, ensartinib, brigatinib, ceritinib and chemotherapy. The HRs of each treatment relative to crizotinib, the ranking of treatments over time, and the survival curves predicted by the RP model all support similar ranking, more details are presented in Additional file 5, eFigures 5–7. PH assumption held in this network, and when compared HRs, only alectinib significantly improved OS compared to crizotinib (HR, 0.67 [0.46 ~ 0.98]). More details are provided in Fig. 3A. Relative outcomes of 24-month OS rate demonstrated comparable outcomes, with lorlatinib and alectinib emerging as the top-performing regimens (Fig. 5). Subgroup analysis based on the baseline presence of BM showed that alectinib remained the preferred choice for patients, regardless of their baseline BM status (Additional file 5, eFigure 4). The league table is presented in Additional file 5, eTable 4.

We conducted additional sensitivity analyses to assess the impact of "crossover" on study outcomes. Specifically, we independently modeled scenarios where crossover in control groups was either permitted or not permitted. The results indicate that, in trials disallowing crossover, alectinib had the highest ranking compared to crizotinib (HR, 0.67 [0.46 ~ 0.98]), followed by lorlatinib and ensartinib. In contrast, in trials allowing crossover, brigatinib ranked highest compared to crizotinib (HR, 0.81 [0.53–1.23]), followed by ceritinib and chemotherapy. For details, see Additional file 5, eFigure 4.

#### Progression-free survival for first-line treatments

### Systemic progression-free survival

Until 27 months, evidence from RMST models indicates that lorlatinib (RSMD, 8.67 months [95%CI, 6.44 ~ 10.92]) significantly improved PFS most compared to crizotinib. In 42-month follow-up, lorlatinib was still the optimal choice, followed by alectinib, which had an RMSD about 1.2 months compared to brigatinib. As shown in Additional file 5, eFigure 3, all ALK-inhibitors significantly improved PFS compared with chemotherapy, results of over time RMSD shown consistent conclusion (Fig. 1 2A-2B). The FP model with *P* = 0 provided the best fit for the data (Fig. 2B). Lorlatinib exhibited the highest PFS rate in the long-term. Time-varying HRs, treatment ranking over time and survival curves predicted by RP model are presented in Additional file 5, eFigures 5–7. Evidence from Cox-PH model are shown in Fig. 3B, the league table is provided in Additional file 5, eTable 4. Consistently, lorlatinib also exhibited the highest 12-month PFS rate (Fig. 5).

For Asian patients, the RMST model showed that compared with crizotinib over a 21-month period, alectinib significantly improved PFS most (RSMD 5.73 months [95% CI, 3.70 ~ 7.74]). All ALK inhibitors significantly improved PFS compared to chemotherapy, with alectinib performed better than other treatments (Fig. 1 3A-3B and Additional file 1, eFigure 3). The best-fitted FP model (*P* = 1) predicted that in a longer follow-up, alectinib appeared to be the optimal choice in PFS, while ensartinib was suboptimal (Fig. 2C). Time-varying HRs, treatment ranking over time and survival curves predicted by RP model are provided in Additional file 5, eFigures 5–7. Results of the Cox-PH model are provided in Fig. 3C and Additional file 5, eTable 4. Our findings were consistent when considering short-term IRC-accessed data from ALEX and ALESIA (Additional file 7 Part A-B). Results for patients with or without baseline BM are showed in Additional file 5, eFigure 8. (during 16 months) and Additional file 5, eFigure 9 (a longer follow-up). Details about survival curves, time-varying HRs and treatment ranking for these subgroups are provided in Additional file 5, eFigures 5–7. Evidence from Cox-PH model is provided in Additional file 5, eFigure 10 and eTable 4. Similarly, findings from the 12-month PFS rate revealed that ensartinib and alectinib were the top-performing regimens.

Time-invariant Cox-PH models were applied in other subgroups. For non-Asian and smoking subgroups, lorlatinib, alectinib and brigatinib were treatments that had significantly longer PFS compared to crizotinib. For patients over the age of 65, lorlatinib and alectinib performed significantly better than crizotinib. More details are shown in Additional file 5, eFigure 11 and eTable 4.

### Intracranial progression-free survival

As depicted in Fig. 1, for patients with baseline BM, the evidence from RMST models suggests that compared to crizotinib over a 16-month follow-up, lorlatinib significantly improved PFS most (RSMD, 6.86 months [95% CI, 4.74 ~ 8.95]). For patients without baseline BM, alectinib(5.63 months [95% CI, 3.73 ~ 7.55]) significantly improved PFS most compared with crizotinib in 25-month.No significant difference was observed among these treatments. In a longer follow-up, both FP models (*P* = 0 and 1 for intracranial and non-intracranial PFS network respectively) and RP models suggested that lorlatinib was the optimal choice, followed by alectinib. More details see Fig. 2 E–F, eFigure 3 and Additional file 5, eFigure 5–7. Results of Cox-PH models consistently support the conclusion, more details can be seen in Fig. 3 E–F and Additional file 5, eTable 4.

#### Progression-free survival for second-line treatments

Evidence from RMST models based on three trials indicated that compared with chemotherapy, alectinib (RSMD, 7.96 months [95%CI, 6.02 ~ 9.91]) significantly improved PFS of patients previously given crizotinib most. In the long-term, the best-fitted FP models (*P* = 0) predicted that alectinib and brigatinib were the best options. More details see Fig. 2D and Additional file 5, eFigure 3. As Fig. 3D shows, assuming HRs are time-invariant, compared with chemotherapy, alectinib (HR 0.20 [95% CI, 0.12 ~ 0.33]) and brigatinib (0.19 [0.10 ~ 0.37]) were the better options, followed by ceritinib (0.49 [0.36 ~ 0.67]). The league table is provided in Additional file 5, eTable 4. While using short-term IRC-accessed data of ALUR, overall results remained consistent (Additional file 7 Part C). As only Profile 1007 reported effects of ALK-inhibitors in chemotherapy naive patients [15], related NMA was not feasible for such patients.

#### Objective response rate

**Fig. 4:**
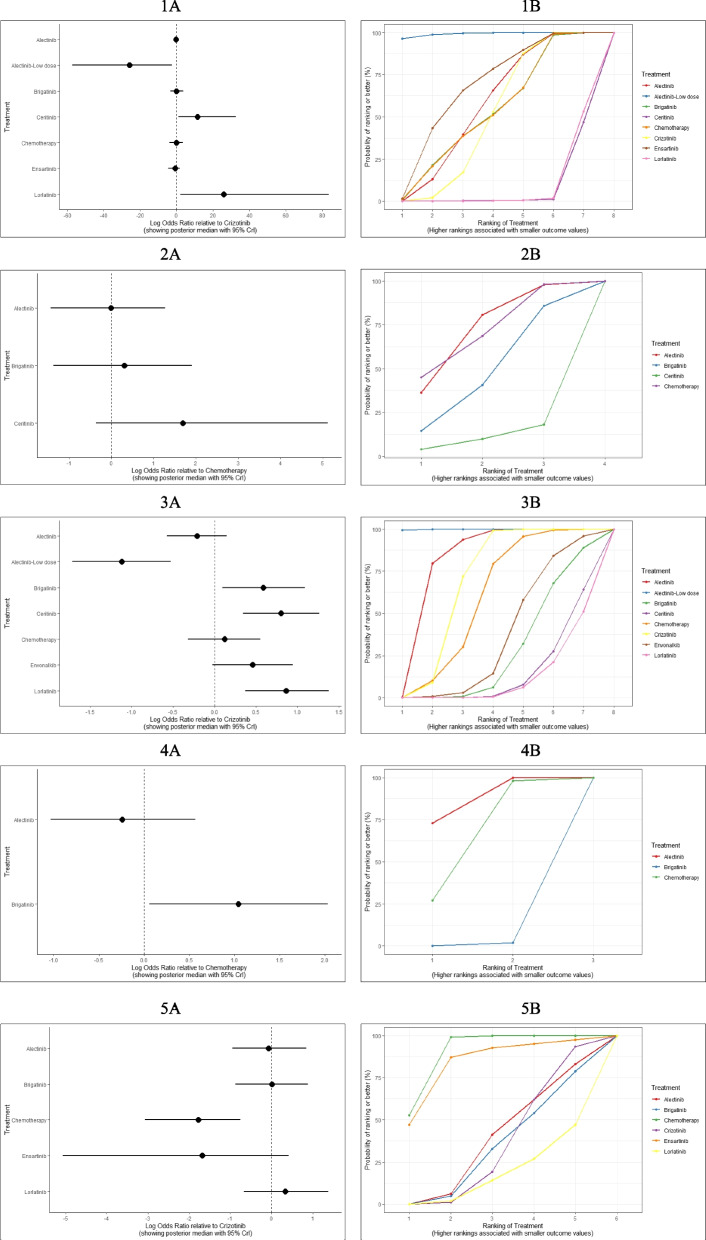
Summary Results of AE (1. Any-grade AE for first-line treatment; 2. Any-grade AE for second-line treatment; 3. Grade 3–4 AE for first-line treatment; 4. Grade 3–4 AE for second-line treatment; 5. Grade 5 or fatal AE for first-line treatment.)

For systemic ORR of first-line treatments, compared to crizotinib, low-dose alectinib (OR, 3.03 [95% CI, 1.21 ~ 8.17]), lorlatinib (2.41 [1.46 ~ 4.01]), ceritinib (2.18 [1.12 ~ 4.10]), alectinib (1.92 [1.20 ~ 3.13]) and envonalkib (1.92 [1.08 ~ 3.49]) performed significantly better; followed by ensartinib (1.43 [0.87 ~ 2.39]) and brigatinib (1.28 [0.76 ~ 2.18]). For intracranial ORR of first-line treatments, envonalkib (13.07 [4.48 ~ 41.68]), brigatinib (12.43 [4.66 ~ 36.23]), lorlatinib (9.03 [3.29 ~ 28.79]), alectinib (7.54 [1.40 ~ 49.40]) and ensartinib (6.42 [2.59 ~ 16.44]) were associated with significant advantages compared to crizotinib, ceritinib (1.92 [0.36 ~ 10.80]) showed similar efficacy as crizotinib. Regarding ORR and intracranial ORR in second-line treatments for crizotinib-treated patients, alectinib and brigatinib performed better compared to ceritinib and chemotherapy. Notably, alectinib and brigatinib were significantly superior to ceritinib for intracranial ORR. ALK-inhibitors were significantly improved ORR or intracranial ORR compared with chemotherapy. Forest and rank plots are provided in Fig. 4, league tables presenting the logarithm ORs for all possible treatment comparisons is available in Additional file 5, eFigure 12.

### Safety

Compared to crizotinib, low-dose alectinib (logarithm OR, -20.46 [95% CI, -58.03 ~ 1.79]) significantly decreased the incidence of any-grade AEs in first-line treatments. Conversely, lorlatinib (25.63 [2.02 ~ 85.01]) and ceritinib (30.74 [2.47 ~ 70.01]) significantly increased the incidence of any-grade AEs. In terms of any-grade AEs of second-line treatments, chemotherapy and alectinib were found to be the safest options, followed by brigatinib and ceritinib. No significant difference was observed among the four treatments. Regarding grade 3–4 AEs of first-line treatments, compared to crizotinib, low-dose alectinib (OR, 0.33 [95% CI, 0.18 ~ 0.59]) significantly reduced incidence. Envonalkib significantly increased AEs compared to low-dose alectinib and alectinib. As for second-line treatments, alectinib appeared to be the safest in terms of grade 3–4 AEs, whereas brigatinib performed the worst in safety. For grade 5 or fatal AEs, chemotherapy had the highest safety while lorlatinib had the lowest. Forest or rank plots are shown in Fig. 4, league tables presenting the logarithm ORs for all comparisons are available in Additional file 5, eFigure 14. The incidences of grade 3–4 AEs with rates over 5% are concluded in Additional file 5, eTable 2. Specifically, high incidences of increased blood creatine phosphokinase, lipase increased, hypertension and pneumonia were related to brigatinib. Ceritinib was associated with high rates of increased alanine aminotransferase, aspartate aminotransferase, and gamma-glutamyltransferase. Lorlatinib exhibited clear signs of hyperlipidemia, hypertension, weight gain, and other central nervous system AEs such as cognitive and mood effects.

### Patient-reported outcomes

**Fig. 5:**
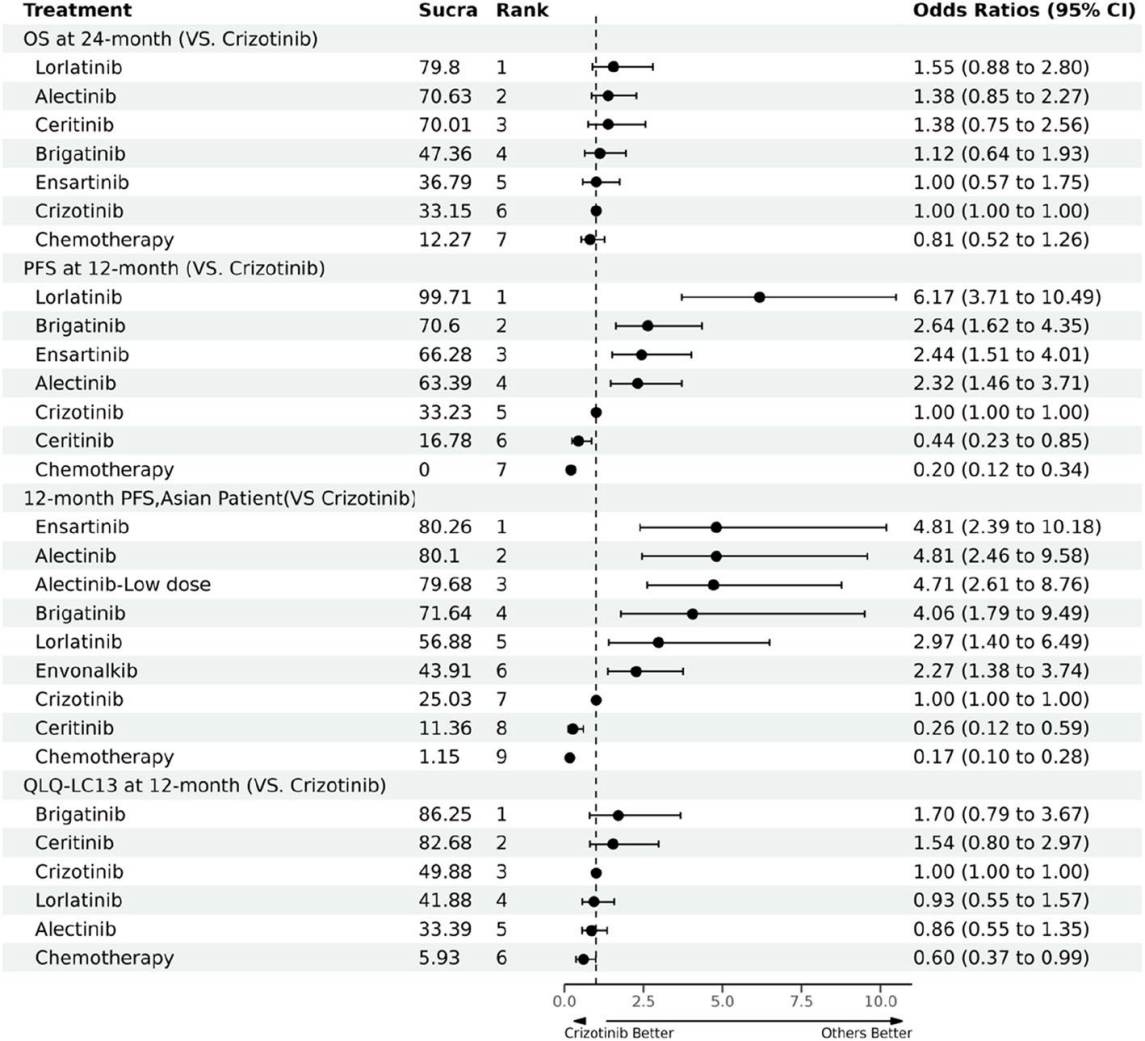
Relative Outcomes of 12-month Progression-free Survival Rate, 24-month Overall Survival Rate, and 12-month Non-deterioration Rate of Patient-reported Outcomes

For NMA of QLQ-LC13 composite score, evidence from RMST models indicated that compared with crizotinib over a 16-month follow-up, brigatinib ranked first (RSMD, 1.28 months [95%CI, -0.85 ~ 3.40]) (Additional file 5, eFigure 3 and eFigure 7). The best-fitted FP model (*P* = 0.5) indicated that brigatinib had slight advantages over other options in the long-term, while alectinib was deemed a suboptimal choice (Additional file 5, eFigure 8). The relative outcomes for the 12-month non-deterioration rate of the QLQ-LC13 composite score suggest that brigatinib remains the optimal choice (Fig. 5). For QLQ-C30 global health status, brigatinib had the best performance in increasing QoL compared to other treatments (Additional file 5, eFigures 7–8). No significant difference was found in both aspects among the ALK-inhibitors. Results of over time HRs, ranking plots and survival curves are provided in Additional file 5, eFigure 5–7. Cox-PH model provided consistent conclusion, more details see Additional file 5, eFigure 9 and eTable 4. Results of changes in the global health status score and individual symptom scores for each RCT have been summarized in Additional file 5, eTable 5.

### Convergence and heterogeneity assessment

The Gelman-Rubin method revealed that the three Markov chains were stable and replicable of the inferential iterations in all models. Overall, the findings of the Q-test, the I^2^ statistic, and the forest plots all indicated low heterogeneity across the specific arm. More details are provided in Additional file 5, eTable 3.

## Discussion

Our study compared systemic treatments for advanced ALK-rearranged NSCLC to inform decision-making. We conducted a comprehensive search for eligible RCTs, critically appraised their quality, synthesized their data and ranked treatments based on their efficacy, safety, and PROs as depicted in the RCTs. We identified 14 eligible studies and constructed scarce networks, in which most treatments have not been compared in head-to-head trials, which underscored the significance of our study. Regarding first-line OS, alectinib, lorlatinib and ceritinib were three regimens that have significant advantages over other ALK-inhibitors and chemotherapy, and alectinib seemed to be the most advantageous option for both short-term and long-term effectiveness. Analysis of scenarios with and without control group crossover showed that in non-permitting trials, alectinib led the ranking, followed by lorlatinib, ensartinib, and crizotinib. When crossover was allowed, brigatinib ranked highest, then ceritinib, crizotinib, and chemotherapy. Alectinib was the only treatment significantly outperforming crizotinib. However, current data does not conclusively support alectinib’s OS superiority. Even with crossover limits in the CROWN, crizotinib-resistant patients could still access alectinib or brigatinib post-treatment, with only 2.9% given conventional chemotherapy; by contrast, 23.6% in the ALEX trial received chemotherapy post-crizotinib resistance [16, 22, 47]. Data constraints, such as detailed OS for specific subsequent treatments, impede assessing impact from treatment variations. Additionally, the OS data from the CROWN are not yet mature. Therefore, further data is needed to verify alectinib's OS benefit [16, 22]. Notably, in the ALTA-1L and PROFILE 1014, adjusted HR values for crossover were reported using model-based methods like the marginal structural model, with brigatinib showing significant OS benefit over crizotinib (HR, 0.54 [0.31–0.92]) [10, 44]. Nonetheless, due to potential biases in model-derived HRs versus true RCT results without permitted crossover, we excluded these outcomes.

For first-line PFS of global patients, lorlatinib seemed to the optimal choice, followed by alectinib and brigatinib. Similarly to crizotinib, ceritinib showed significantly worse performance than other ALK-inhibitors. Consistent conclusions were observed in subgroup analysis based on ECOG performance status, baseline BM, gender, age, and smoking status. However, for Asian patients, alectinib demonstrated significant improvement in PFS when compared to other treatments. For crizotinib-treated patients, alectinib and brigatinib proved to be the top choices in terms of PFS, with a potential slight edge for alectinib over brigatinib. For intracranial PFS, both lorlatinib and alectinib were found to be potentially optimal choices irrespective of the presence of baseline brain metastases. Additionally, low-dose alectinib and alectinib demonstrated the highest ORR for first- and second-line treatment, respectively. In terms of safety, low-dose alectinib and alectinib exhibited superior performance overall in any-grade, grade 3 + AEs. On the other hand, lorlatinib and ceritinib were associated with a substantial increase in any-grade AEs, and lorlatinib and brigatinib were found to be significantly worse in grade 3–4 AEs compared to crizotinib. As for PROs, based on the results of QLQ-LC13 and QLQ-C30, brigatinib appeared to be the best option. Noteworthily, for lorlatinib, in addition to increase AEs, reduce QoL, compared to other ALK inhibitors, the prognostic options for lorlatinib-resisted patients are worse [53]. Considering the comparable effectiveness of alectinib and loratinib, alectinib may be the preferred option for initial treatment worldwide.

Our study provides several insights. Firstly, we evaluated relative efficacy of second-line treatments for patients previously given crizotinib, which was not evaluated in previous reviews. Secondly, we are the first to compare PROs. Nowadays, cancer treatment philosophy has changed to “perfect control of the disease with perfect quality of life” [38], thus, PROs is a crucial factor in the selection of clinical drugs. Thirdly, this is the first IPD-based NMA in the area of ALK-rearranged NSCLC. We innovatively used RMST as a short-term outcome indicator. Compared with HR, non-parametric RMST has a much clearer clinical interpretation, especially when PH assumption is invalid [54]. Besides, we closely modeled the observed Kaplan–Meier curves and validated the robustness of results against different assumptions regarding HRs (time invariant vs. time varying), this analysis is crucial given that non-PH were detected in many included RCTs. Long-term effectiveness was also firstly predicted by us using FP and RP models.

We have confirmed and updated the findings from previous reviews. Currently, there are several similar NMAs available [38, 55–64]. The reviews in question focused solely on the efficacy and safety of first-line treatments in the global population, without validating PH assumptions and only considering Cox-PH models. Meanwhile, analysis of post-line treatments, subgroup analysis or evaluation of PROs were also lost, making it difficult to provide sufficient evidence to guide the clinical use of drugs. Furthermore, over half of the data from RCTs was updated during 2022, and two RCTs reported interim results for the first time. Using the updated data, our study confirmed the conclusions drawn from prior assessments. Specifically, loratinib emerged as the most effective treatment in terms of PFS for global patients, but it also exhibited the worst safety profile. Alectinib outperformed other treatments regarding OS and had low AEs. Low-dose alectinib was the safest option. Moreover, our research uncovered several innovative results. Firstly, alectinib was found to be the optimal choice for patients who were resistant to crizotinib. Secondly, alectinib was found to be the best option for Asian patients in terms of PFS, while loratinib had the best PFS for patients with other characteristics. Thirdly, loratinib and alectinib were identified as the best options for patients with or without baseline BM, respectively, in terms of intracranial PFS. Fourthly, brigatinib appeared to be the best option for patients in terms of PROs. Finally, the safety and efficacy of envonalkib were firstly compared to other ALK-inhibitors.

This study holds great importance for patients, clinicians, and payers due to the ambiguity surrounding the ideal treatment for ALK-rearranged NSCLC. In the age of precision therapy, it is insufficient to limit research to comparing only first-line treatments. Deciding on the best post-line treatment and selecting the most suitable treatment option based on patient characteristics are questions that cannot be overlooked. Moreover, as disease control improves, emphasis must also be placed on PROs. Our study can effectively guide the clinical use of drugs in these aforementioned areas.

### Limitations

There is heterogeneity among clinical trials in terms of patient inclusion, trial design and evaluation methods of disease metastases. Second- or third-generation ALK inhibitors, as compared to chemotherapy or first-generation ALK inhibitors, significantly improve efficacy for patients with baseline BM. For instance, in the CROWN, lorlatinib vs. crizotinib had an HR for PFS of 0.2 (0.1–0.43) in the patients with baseline BM, and an HR of 0.32 (0.2–0.49) in those without baseline BM [16, 22]. This indicated that RCTs with more baseline BM patients tend to show lower HRs for PFS and OS when comparing advanced ALK inhibitors to first-generation or chemotherapy. Baseline BM percentages differed among RCTs; for instance, ALEX had about 40% vs. CROWN's 26% [16, 22, 47]. Furthermore, ALTA-1L exclusively enrolled ALK inhibitor-naïve patients, with approximately 26% previously treated with chemotherapy. While other RCTs involved patients who had not received prior systemic treatment. This means that in the ALTA-1L, patient performance in terms of PFS or OS might be diminished due to the upfront chemotherapy [10]. Likewise, the trial design and evaluation method can affect the study outcome. Crossover medication usage in the control group has implications for OS. Trials like ALEX, CROWN, and eXalt3 did not allow crossover in the control group, while others did. This suggests that trials permitting crossover, including ASCEND-4, ALTA-1L, and PROFILE 1014, may be at a comparative disadvantage in OS analyses [10, 13, 16, 22, 44, 47]. Moreover, the primary outcomes of trials like ALEX were based on investigator assessments, while those like CROWN were based on IRC. Given that the included RCTs were open-label, investigator-assessed PFS might be subjective. Although we conducted multiple subgroup analyses and sensitivity analyses to mitigate the impact of patient characteristics, trial design, and evaluation methods, their influence on the results was still inevitable and difficult to evaluate. Hence, the primary outcomes of this study require cautious interpretation.

Limited by the availability of data, the exploration of OS subpopulations in this study requires further depth. In the J-ALEX based on an Asian cohort, low-dose alectinib vs. crizotinib had an OS HR of 1.05 (0.68–1.61), and the HR of alectinib vs. crizotinib for OS in the ALEX was 0.74 (0.40–1.36) for the Asian population [25, 47]. Moreover, unlike its significant advantage in PFS (HR 0.08, 0.01–0.61), low-dose alectinib vs. crizotinib had an OS HR of 1.56 (0.64 to 3.80) for the baseline BM patients. Correspondingly, the ALTA-1L and ALEX trials indicated that brigatinib and alectinib vs. crizotinib had OS HRs of 0.43 (0.21–0.89) and 0.58 (0.34–1), respectively, in patients with baseline BM [10, 47]. This suggests that although low-dose alectinib may bring PFS improvement and better tolerability, its performance in terms of OS, especially in the BM subgroup, is lacking.

Other potential limitations include: The follow-up durations differed across trials, and the data for some outcomes was immature. In the RMST model, we could only select data within the shortest follow-up durations, which limited the extrapolation of conclusions. For ALEX, ALESIA and ALUR, the updated PFS data used in primary analysis in our NMA was only available from investigator-assessed, considering that the RCTs included in this study were open-label, our results may have bias, although we found results were generally consistent when using short-term IRC-accessed data. For future directions, more direct comparative evidence among ALK inhibitors is necessary to validate our conclusions. Finally, limited data hampers precise comparisons of OS across treatments, factoring in "crossover" and subsequent therapies.

## Conclusions

Alectinib appears to be the preferred first-line option in OS, and Asian-specific PFS, superiority of alectinib also found in intracranial PFS for patients without baseline BM. For second-line treatment of crizotinib-resistant patients, alectinib still is the optimal option. Low-dose alectinib was the best safety option, and brigatinib seemed to be optimal treatment in terms of PROs. Though loratinib performed the best regarding PFS and intracranial PFS for patients in many subgroups, alectinib, which has similar efficacy and a favorable safety and QoL, may be considered a valid alternative.

### Supplementary Information

**Additional file 1.** 

** Additional file 2.** 

** Additional file 3.** 

** Additional file 4.** 

** Additional file 5.** 

** Additional file 6.** 

** Additional file 7.**

## Data Availability

Data sharing statement Full data set is available, on request from the corresponding author, e-mail, tokammy@cpu.edu.cn.

## Abbreviations

ALK: Anaplastic Lymphoma Kinase
NSCLC: Non-Small-Cell Lung Cancer
HRs: Hazard ratios
ORs: Odds ratios
OS: Overall survival
PFS: Progression-free survival
PROs: Patient-reported outcomes
RMST: Restricted mean survival time
ORR: Objective response rate
QoL: Quality of life
PH: Proportional hazards
NMA: Network meta-analysis
AEs: Adverse events
BM: Brain metastasis
ROB: Risk of bias
FP: Fractional Polynomial
RP: Royston-Parmar
AIC: Akaike information criterion
IPD: Individual patient data
QLQ-LC13: Quality of Life Questionnaire: Lung Cancer Module
QLQ-C30: Quality of Life of Cancer Patients Questionnaire global Quality of Life
