# Supplementary material for: Identifying optimal ALK inhibitors in first- and second-line treatment of patients with advanced ALK-positive non-small-cell lung cancer: a systematic review and network meta-analysis
Source: BMC Cancer. 2024 Feb 8;24:186. doi: 10.1186/s12885-024-11916-4 (PMC10851546; doi:10.1186/s12885-024-11916-4)
Supplement: Supplementary file 2 — Additional file 2. [file 12885_2024_11916_MOESM2_ESM.pdf]

Systematic review

A list of fields that can be edited in an update can be found here

1. * Review title.

Give the title of the review in English

Effectiveness and safety of treatment strategies for anaplastic lymphoma kinase positive non-small cell lung cancer: a network meta-analysis

2. Original language title.

For reviews in languages other than English, give the title in the original language. This will be displayed with the English language title.

???????????????????????????????Meta??

3. * Anticipated or actual start date.

Give the date the systematic review started or is expected to start.

01/12/2022

4. * Anticipated completion date.

Give the date by which the review is expected to be completed.

01/07/2023

5. * Stage of review at time of this submission.

This field uses answers to initial screening questions. It cannot be edited until after registration.

Tick the boxes to show which review tasks have been started and which have been completed.

Update this field each time any amendments are made to a published record.

The review has not yet started: No

Review stage	Started	Completed
Preliminary searches	Yes	No
Piloting of the study selection process	No	No
Formal screening of search results against eligibility criteria	No	No
Data extraction	No	No
Risk of bias (quality) assessment	No	No
Data analysis	No	No

Provide any other relevant information about the stage of the review here.

6. * Named contact.

The named contact is the guarantor for the accuracy of the information in the register record. This may be any member of the review team.

Tai hang Shao

Email salutation (e.g. "Dr Smith" or "Joanne") for correspondence:

Mr Shao

7. * Named contact email.

Give the electronic email address of the named contact.

15161834391@163.com

8. Named contact address

Give the full institutional/organisational postal address for the named contact.

639 Longmian Avenue, Jiangning District, Nanjing 211198

9. Named contact phone number.

Give the telephone number for the named contact, including international dialling code.

15161834391

10. * Organisational affiliation of the review.

Full title of the organisational affiliations for this review and website address if available. This field may be completed as 'None' if the review is not affiliated to any organisation.

China Pharmaceutical University

Organisation web address:

11. * Review team members and their organisational affiliations.

Give the personal details and the organisational affiliations of each member of the review team. Affiliation refers to groups or organisations to which review team members belong. **NOTE: email and country now MUST be entered for each person, unless you are amending a published record.**

Mr Tai hang Shao. China Pharmaceutical University
Mingye Zhao. China Pharmaceutical University
Professor Wenxi Tang. China Pharmaceutical University

12. * Funding sources/sponsors.

Details of the individuals, organizations, groups, companies or other legal entities who have funded or sponsored the review.

General Program of National Natural Science Foundation of China

Grant number(s)

State the funder, grant or award number and the date of award

72174207

13. * Conflicts of interest.

List actual or perceived conflicts of interest (financial or academic).

None

14. Collaborators.

Give the name and affiliation of any individuals or organisations who are working on the review but who are not listed as review team members. **NOTE: email and country must be completed for each person, unless you are amending a published record.**

15. * Review question.

State the review question(s) clearly and precisely. It may be appropriate to break very broad questions down into a series of related more specific questions. Questions may be framed or refined using PI(E)COS or similar where relevant.

This study aims to compare the effectiveness and safety of six available ALK-TKI in China for the treatment of advanced ALK-positive non-small cell lung cancer.

16. * Searches.

State the sources that will be searched (e.g. Medline). Give the search dates, and any restrictions (e.g.

language or publication date). Do NOT enter the full search strategy (it may be provided as a link or attachment below.)

The PubMed, Web of Science, ClinicalTrials.gov were searched systematically to retrieve clinical trials and published studies of PD-1/PD-L1; and abstracts published in ESMO (European Society for Medical Oncology), ASCO (American Society of Clinical Oncology)) And WCLC (World Conference on Lung Cancer). There is no restriction on the research time limit, and the language is limited to Chinese or English.

17. URL to search strategy.

Upload a file with your search strategy, or an example of a search strategy for a specific database, (including the keywords) in pdf or word format. In doing so you are consenting to the file being made publicly accessible. Or provide a URL or link to the strategy. Do NOT provide links to your search **results**.

Alternatively, upload your search strategy to CRD in pdf format. Please note that by doing so you are consenting to the file being made publicly accessible.

Do not make this file publicly available until the review is complete

18. * Condition or domain being studied.

Give a short description of the disease, condition or healthcare domain being studied in your systematic review.

According to estimates by the International Agency for Research on Cancer, 19.3 million new cancer cases and nearly 10 million cancer deaths occurred in 2020. Among them, lung cancer accounted for 11.4% of new cancer cases; new deaths accounted for 18%. Non-small cell lung cancer (NSCLC) accounts for about 80%-85% of the total number of lung cancers. However, anaplastic lymphoma kinase (ALK) gene fusions occur in approximately 3-5% of all patients with non-small cell lung cancer.

19. * Participants/population.

Specify the participants or populations being studied in the review. The preferred format includes details of both inclusion and exclusion criteria.

The research population refers to the research subjects of clinical trials, which are patients with stage III and stage IV non-small cell lung cancer with ALK+ aged 18 years and above.

20. * Intervention(s), exposure(s).

Give full and clear descriptions or definitions of the interventions or the exposures to be reviewed. The preferred format includes details of both inclusion and exclusion criteria.

Intervention group: Six available ALK-TKI in China, including Crizotinib, Ceritinib, Brigatinib, Ensartinib, Alectinib, Lorlatinib. The dosage and method of medication will refer to clinical trials and literature reports.

21. * Comparator(s)/control.

Where relevant, give details of the alternatives against which the intervention/exposure will be compared (e.g. another intervention or a non-exposed control group). The preferred format includes details of both inclusion and exclusion criteria.

Mainly crizotinib and chemotherapy

22. * Types of study to be included.

Give details of the study designs (e.g. RCT) that are eligible for inclusion in the review. The preferred format includes both inclusion and exclusion criteria. If there are no restrictions on the types of study, this should be stated.

RCT

23. Context.

Give summary details of the setting or other relevant characteristics, which help define the inclusion or exclusion criteria.

24. * Main outcome(s).

Give the pre-specified main (most important) outcomes of the review, including details of how the outcome is defined and measured and when these measurement are made, if these are part of the review inclusion criteria.

PFS, OS, life years gained and HR(95% CI)

Measures of effect

Please specify the effect measure(s) for you main outcome(s) e.g. relative risks, odds ratios, risk difference, and/or 'number needed to treat.

25. * Additional outcome(s).

List the pre-specified additional outcomes of the review, with a similar level of detail to that required for main outcomes. Where there are no additional outcomes please state ‘None’ or ‘Not applicable’ as appropriate to the review

safety(?3 grade AE), health-related outcomes

Measures of effect

Please specify the effect measure(s) for you additional outcome(s) e.g. relative risks, odds ratios, risk difference, and/or 'number needed to treat.

26. * Data extraction (selection and coding).

Describe how studies will be selected for inclusion. State what data will be extracted or obtained. State how this will be done and recorded.

This study will extract in detail the experimental design of all clinical trials included in the study, and patient baseline characteristics (including the NCT number, age, gender, country, stage, ECOG score, smoking status, brain metastasis, treatment lines, etc.), intervention measures (method of medication, dosage),

outcome indicators (PFS, OS, and HR value and 95% CI of PFS and OS, PFS curve, OS curve) and grade 3-5 adverse reactions

27. * Risk of bias (quality) assessment.

State which characteristics of the studies will be assessed and/or any formal risk of bias/quality assessment tools that will be used.

The risk of bias in included studies will be independently evaluated by two reviewers. Any disagreements over risk of bias will be resolved in discussion with a third reviewer. To assess the risk of bias in controlled trials we will use the Cochrane risk of bias assessment tool (in Reviewer manager 5.3).

28. * Strategy for data synthesis.

Describe the methods you plan to use to synthesise data. This **must not be generic text** but should be **specific to your review** and describe how the proposed approach will be applied to your data. If meta-analysis is planned, describe the models to be used, methods to explore statistical heterogeneity, and software package to be used.

Combination of survival data based on proportional hazards/non proportional hazards assumption. Indirect comparison including methods of Generalized Linear Model and Bayesian models (including Fractional polynomials, Generalized gamma model, RP model, etc.)

29. * Analysis of subgroups or subsets.

State any planned investigation of ‘subgroups’. Be clear and specific about which type of study or participant will be included in each group or covariate investigated. State the planned analytic approach.

subgroups will be conducted according to treatment lines (first-line and second-line) and region (we will specially focus on Asian people)

30. * Type and method of review.

Select the type of review, review method and health area from the lists below.

Type of review

Cost effectiveness

No

Diagnostic

No

Epidemiologic

No

Individual patient data (IPD) meta-analysis

No

Intervention

No

Living systematic review

No

Meta-analysis

No

Methodology

No

Narrative synthesis

No

Network meta-analysis

Yes

Pre-clinical

No

Prevention

No

Prognostic

No

Prospective meta-analysis (PMA)

No

Review of reviews

No

Service delivery

No

Synthesis of qualitative studies

No

Systematic review

Yes

Other

No

Health area of the review

Alcohol/substance misuse/abuse

No

Blood and immune system

No

Cancer

Yes

Cardiovascular

No

Care of the elderly

No

Child health

No

Complementary therapies

No

COVID-19

No

Crime and justice

No

Dental

No

Digestive system

No

Ear, nose and throat

No

Education

No

Endocrine and metabolic disorders

No

Eye disorders

No

General interest

No

Genetics

No

Health inequalities/health equity

No

Infections and infestations

No

International development

No

Mental health and behavioural conditions

No

Musculoskeletal

No

Neurological

No

Nursing

No

Obstetrics and gynaecology

No

Oral health

No

Palliative care

No

Perioperative care

No

Physiotherapy

No

Pregnancy and childbirth

No

Public health (including social determinants of health)

No

Rehabilitation

No

Respiratory disorders

No

Service delivery

No

Skin disorders

No

Social care

No

Surgery

No

Tropical Medicine

No

Urological

No

Wounds, injuries and accidents

No

Violence and abuse

No

31. Language.

Select each language individually to add it to the list below, use the bin icon to remove any added in error.

English

There is not an English language summary

32. * Country.

Select the country in which the review is being carried out. For multi-national collaborations select all the countries involved.

China

33. Other registration details.

Name any other organisation where the systematic review title or protocol is registered (e.g. Campbell, or The Joanna Briggs Institute) together with any unique identification number assigned by them. If extracted data will be stored and made available through a repository such as the Systematic Review Data Repository (SRDR), details and a link should be included here. If none, leave blank.

34. Reference and/or URL for published protocol.

If the protocol for this review is published provide details (authors, title and journal details, preferably in Vancouver format)

Add web link to the published protocol.

Or, upload your published protocol here in pdf format. Note that the upload will be publicly accessible.

No I do not make this file publicly available until the review is complete

Please note that the information required in the PROSPERO registration form must be completed in full even if access to a protocol is given.

35. Dissemination plans.

Do you intend to publish the review on completion?

No

Give brief details of plans for communicating review findings.?

36. Keywords.

Give words or phrases that best describe the review. Separate keywords with a semicolon or new line. Keywords help PROSPERO users find your review (keywords do not appear in the public record but are included in searches). Be as specific and precise as possible. Avoid acronyms and abbreviations unless these are in wide use.

37. Details of any existing review of the same topic by the same authors.

If you are registering an update of an existing review give details of the earlier versions and include a full bibliographic reference, if available.

38. * Current review status.

Update review status when the review is completed and when it is published.New registrations must be ongoing so this field is not editable for initial submission.

Please provide anticipated publication date

Review_Ongoing

39. Any additional information.

Provide any other information relevant to the registration of this review.

Our study targeted on more specific population and the methods we used were the latest.

40. Details of final report/publication(s) or preprints if available.

Leave empty until publication details are available OR you have a link to a preprint (NOTE: this field is not editable for initial submission). List authors, title and journal details preferably in Vancouver format.

Give the link to the published review or preprint.
