# Supplementary material for: Identifying optimal ALK inhibitors in first- and second-line treatment of patients with advanced ALK-positive non-small-cell lung cancer: a systematic review and network meta-analysis
Source: BMC Cancer. 2024 Feb 8;24:186. doi: 10.1186/s12885-024-11916-4 (PMC10851546; doi:10.1186/s12885-024-11916-4)
Supplement: Supplementary file 3 — Additional file 3. [file 12885_2024_11916_MOESM3_ESM.docx]

**Additional file 3**

1. PubMed Search Strategy

| 1 Non-small cell lung cancer | ("Carcinoma, non-small cell lung"[Mesh] OR " carcinomas, non-small cell lung" OR "lung carcinoma, non-small cell" OR " lung carcinomas, non-small cell " OR "non-small cell lung carcinomas" OR "nonsmall cell lung cancer" OR "non-small-cell lung carcinoma" OR "non small cell lung carcinoma" OR "carcinoma, non-small cell lung " OR "non-small cell lung cancer") |
| --- | --- |
| 2  ALK rearrangement | ("ALK" OR "ALK-mutant" OR "ALK mutations" OR "alk" OR "ALK rearrangement " OR "anaplastic lymphoma kinase " OR "ALK-mutant patients" OR "patients with ALK mutations" OR "NPM-ALK" OR "ALK Kinase" OR "CD246 Antigen") |
| 3  RCT | (randomized controlled trial[pt] OR controlled clinical trial[pt] OR randomized[tiab] OR placebo[tiab] OR clinical trials as topic[mesh:noexp] OR randomly[tiab] OR trial[ti]) |
| 4 | (animals[mh] NOT humans [mh]) |
| 5 | **1** AND **2** AND **3** |
| 6 | **5** NOT **4** |

#Search Strategy of Web of Science is similar to this.

1. Embase

**#1** 'Anaplastic Lymphoma Kinase':ti,ab,kw,de,tn OR 'ALK Tyrosine Kinase Receptor':ti,ab,kw,de,tn OR 'Anaplastic Lymphoma Receptor Tyrosine Kinase':ti,ab,kw,de,tn OR 'Nucleophosmin-Anaplastic Lymphoma Kinase':ti,ab,kw,de,tn OR 'Nucleophosmin Anaplastic Lymphoma Kinase':ti,ab,kw,de,tn OR 'ALK Kinase':ti,ab,kw,de,tn OR 'CD246 Antigen':ti,ab,kw,de,tn

**#2** 'Carcinoma, Non-Small-Cell Lung':ti,ab,kw,de,tn OR 'Carcinoma, Non Small Cell Lung':ti,ab,kw,de,tn OR 'Carcinomas, Non-Small-Cell Lung':ti,ab,kw,de,tn OR 'Lung Carcinoma, Non-Small-Cell':ti,ab,kw,de,tn OR 'Lung Carcinomas, Non-Small-Cell':ti,ab,kw,de,tn OR 'Non-Small-Cell Lung Carcinomas':ti,ab,kw,de,tn OR 'Non-Small-Cell Lung Carcinoma':ti,ab,kw,de,tn OR 'Non Small Cell Lung Carcinoma':ti,ab,kw,de,tn OR 'Carcinoma, Non-Small Cell Lung': ti,ab,kw,de,tn OR 'Non-Small Cell Lung Carcinoma':ti,ab,kw,de,tn OR 'Non-Small Cell Lung Cancer':ti,ab,kw,de,tn OR 'Nonsmall Cell Lung Cancer':ti,ab,kw,de,tn

**#3** 'Randomized Controlled Trial':ti,ab,kw,de,tn OR 'Clinical Trials, Randomized':ti,ab,kw,de,tn OR 'Trials, Randomized Clinical':ti,ab,kw,de,tn OR ' Controlled Clinical Trials, Randomized':ti,ab,kw,de,tn

**#4** #1 AND #2 AND #3

1. The Cochrane Central Register of Controlled Trials (CENTRAL)

**#1** (Anaplastic Lymphoma Kinase or ALK Tyrosine Kinase Receptor or Anaplastic Lymphoma Receptor Tyrosine Kinase or Nucleophosmin-Anaplastic Lymphoma Kinase or Nucleophosmin Anaplastic Lymphoma Kinase or ALK Kinase or CD246 Antigen)

**#2** (Carcinoma, Non-Small-Cell Lung or Carcinoma, Non Small Cell Lung or Carcinoma, Non Small Cell Lung or Lung Carcinomas, Non-Small-Cell or Non-Small-Cell Lung Carcinomas or Non-Small-Cell Lung Carcinoma or Non Small Cell Lung Carcinoma or Carcinoma, Non-Small Cell Lung or Non-Small Cell Lung Carcinoma or Carcinoma, Non-Small Cell Lung or Non-Small Cell Lung Carcinoma or Non-Small Cell Lung Cancer or Nonsmall Cell Lung Cancer):ti,ab,kw

**#3** (Randomized Controlled Trial or Clinical Trials, Randomized or Trials, Randomized Clinical or Controlled Clinical Trials, Randomized):ti,ab,kw *(Randomized controlled trials were defaulted in CENTRAL of The Cochrane Library Central Register)
