# Supplementary material for: Identifying optimal ALK inhibitors in first- and second-line treatment of patients with advanced ALK-positive non-small-cell lung cancer: a systematic review and network meta-analysis
Source: BMC Cancer. 2024 Feb 8;24:186. doi: 10.1186/s12885-024-11916-4 (PMC10851546; doi:10.1186/s12885-024-11916-4)

**Additional file 4**

1. **Log-log Plots Showing Time-varying HRs (Take Progression-free Survival as A Example)**


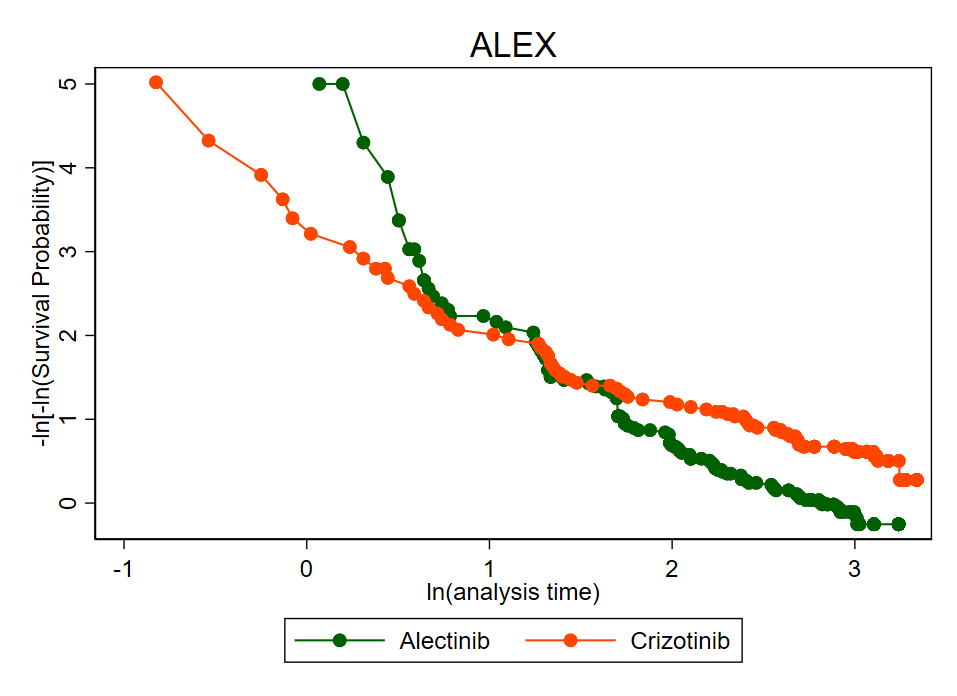


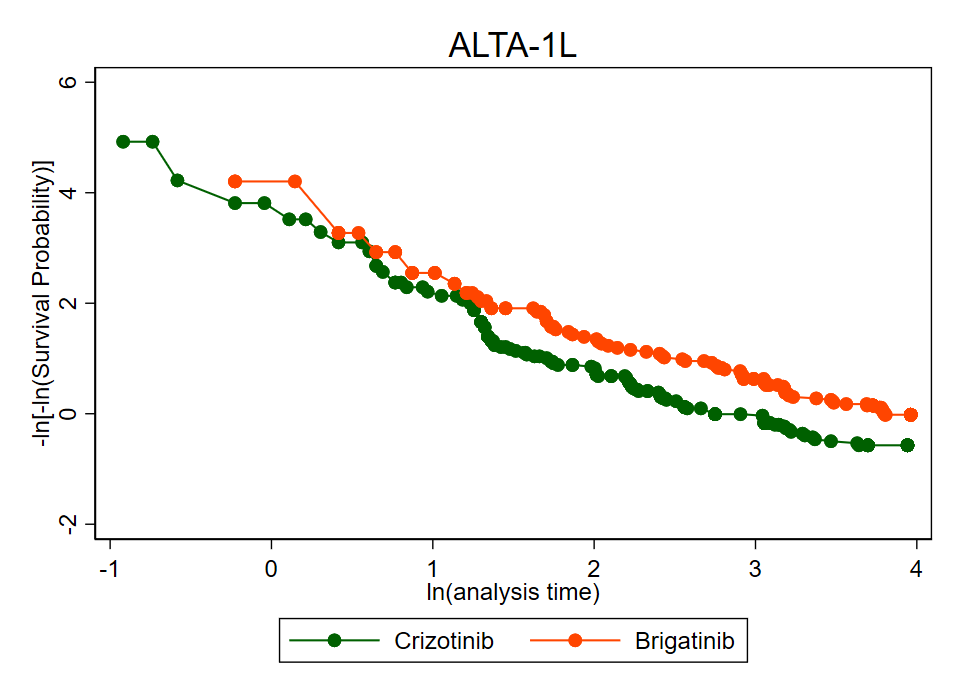


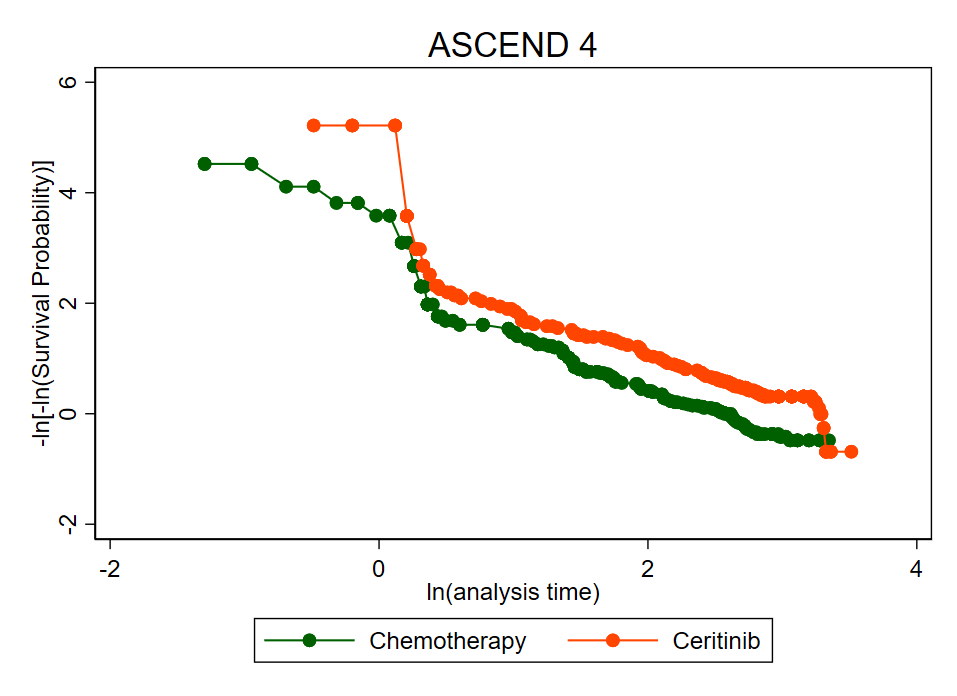


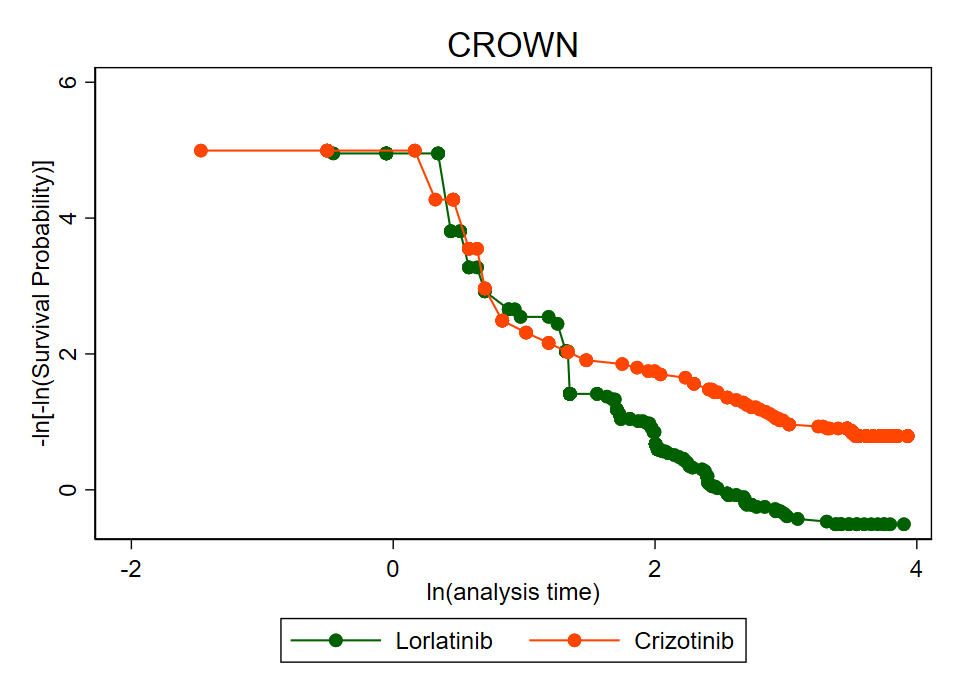


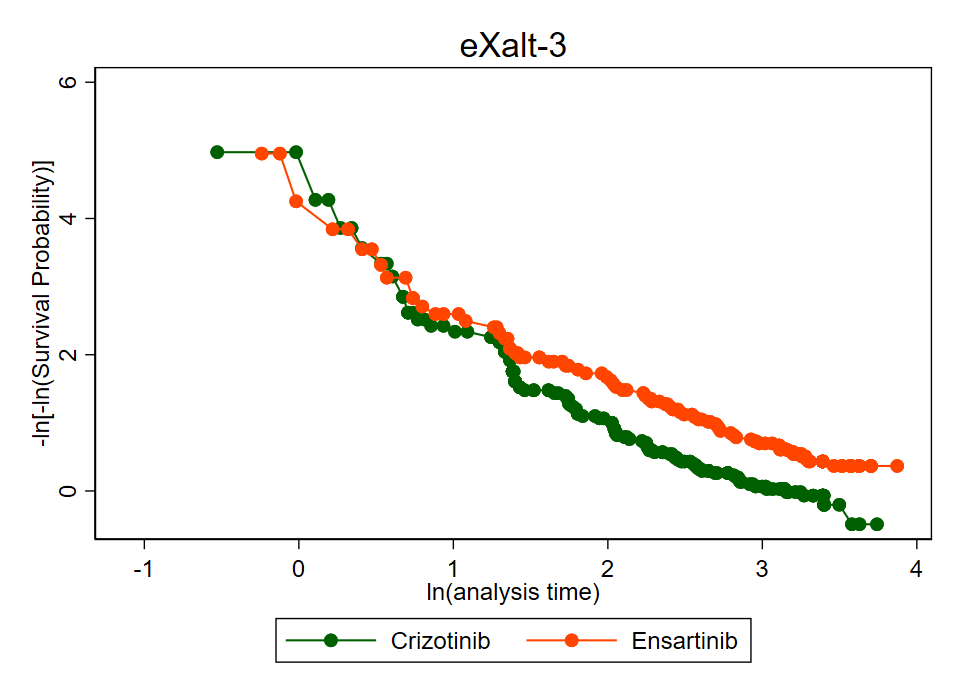


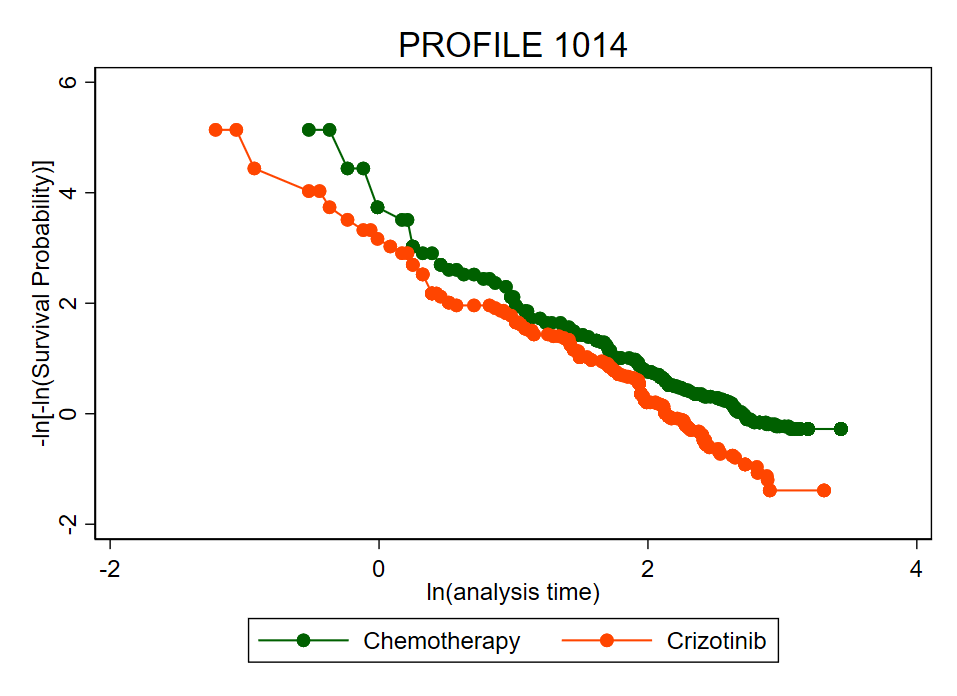


1. **Nonproportional hazards**

**1. Network: PFS for global patients**

| ALEX | CROWN |
| --- | --- |
| 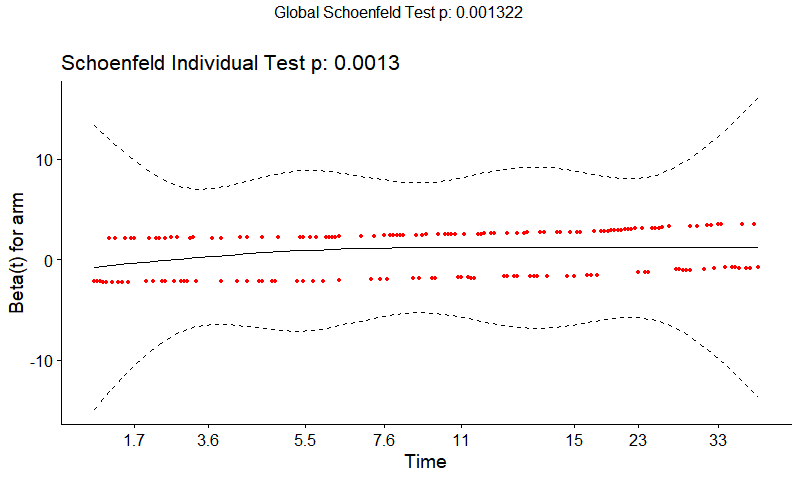 | 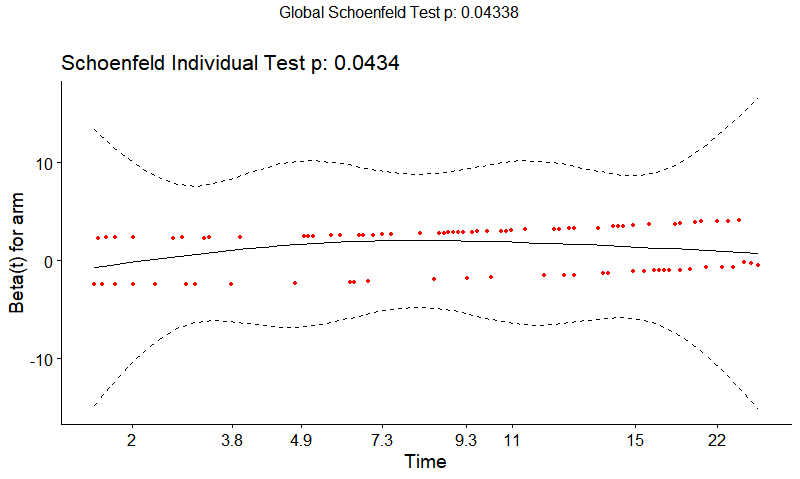 |
| Profile 1014 |  |
| 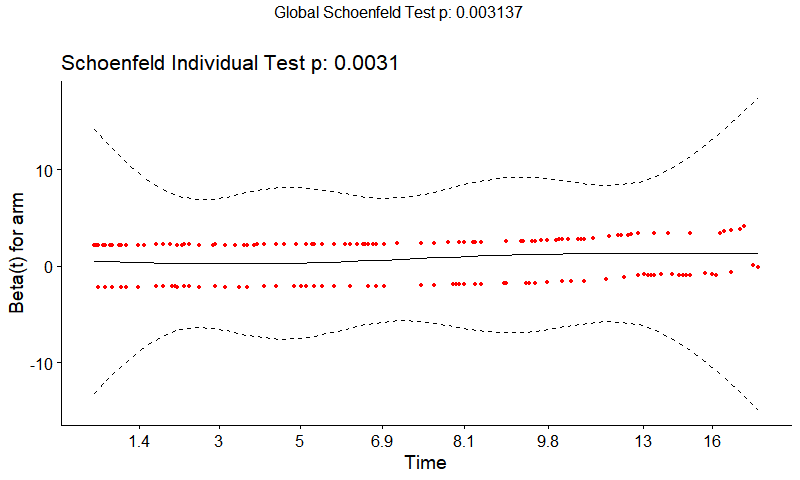 |  |

**2. Network: PFS for Asian patients**

| ALESIA | ASCEND-4 |
| --- | --- |
| 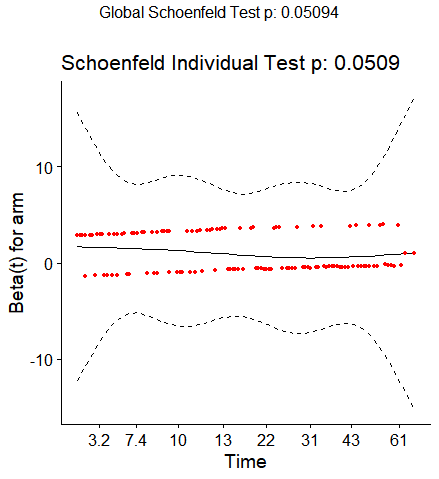 | 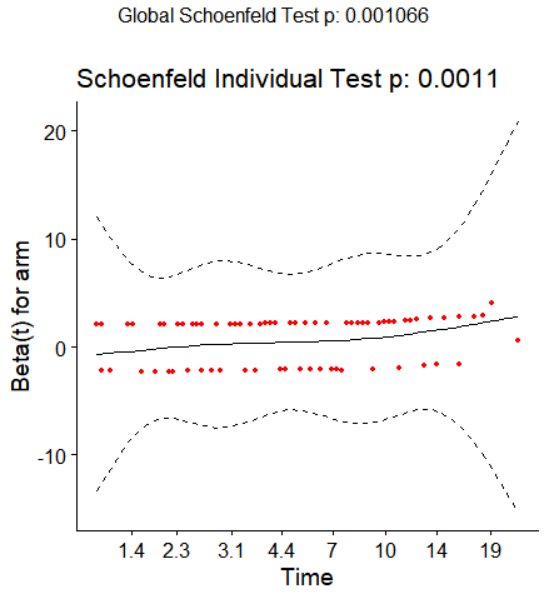 |
| Profile 1014 | Profile 1029 |
| 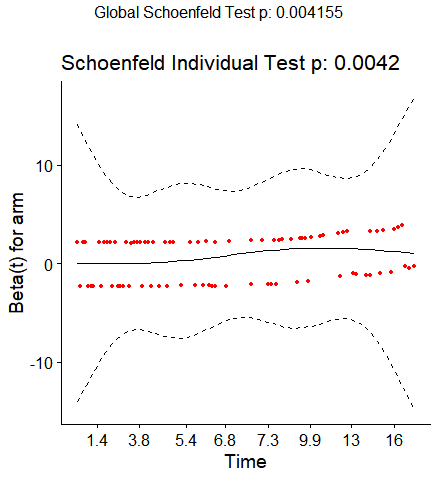 | 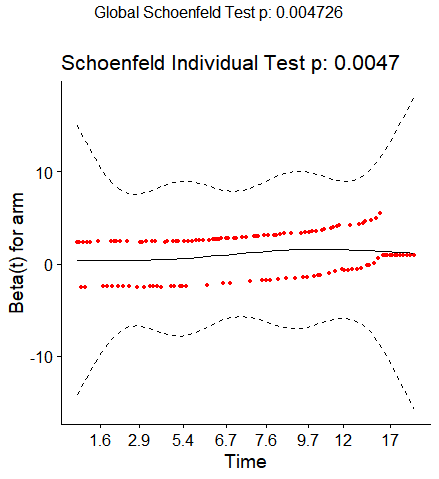 |

**3. Network: Second-line PFS for global patients**

ASCEND-5


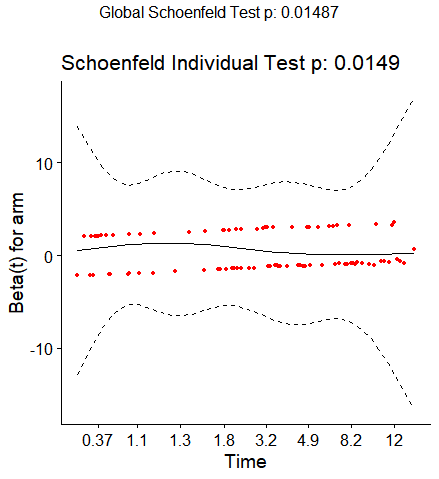


**4. Network: CNS PFS for patients with baseline brain metastasis**

| ALEX | ALTA-1L |
| --- | --- |
| 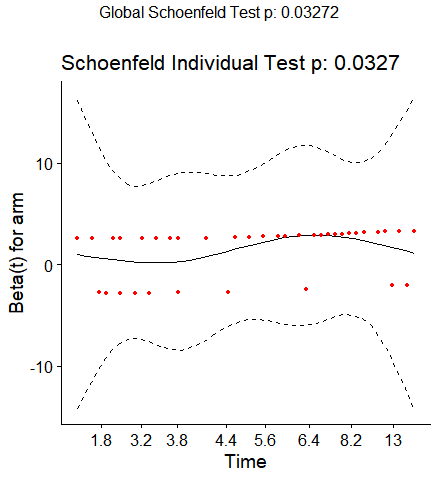 | 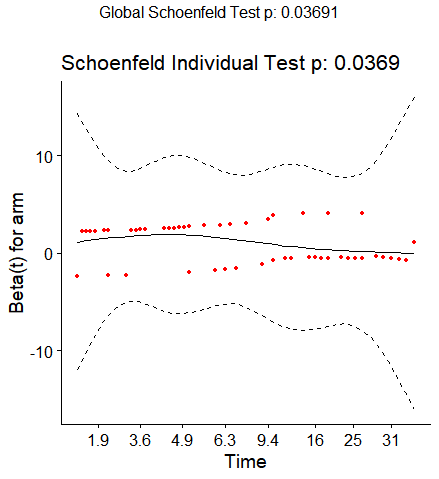 |

**5. Network: CNS PFS for patients without baseline brain metastasis**

eXalt3

**
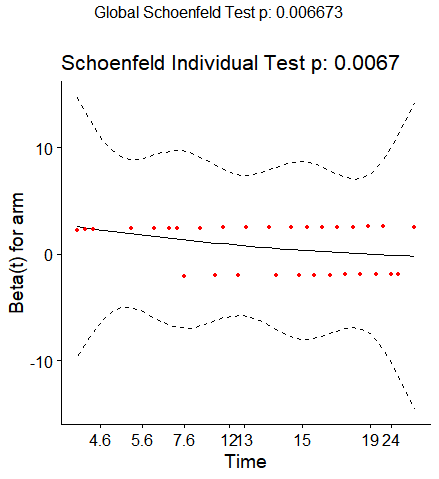
**

**6. Network: PFS for patients with baseline brain metastasis**

| ALEX | ALTA-1L |
| --- | --- |
| 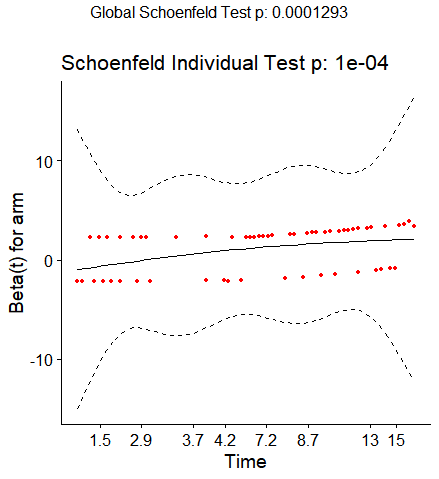 | 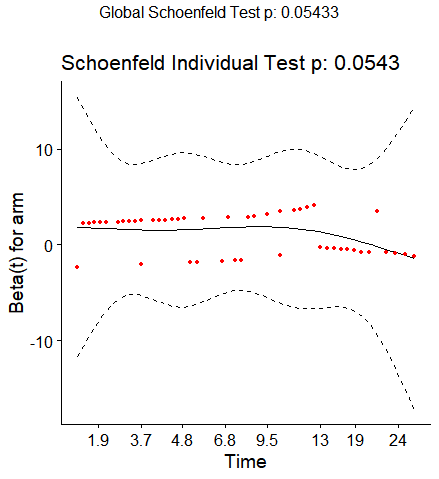 |

**7. Network: QLQ-C30**

ALTA-3


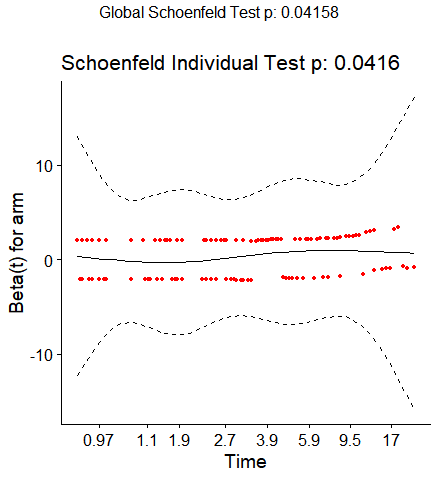


1. **Methodology quality of the included studies**

**
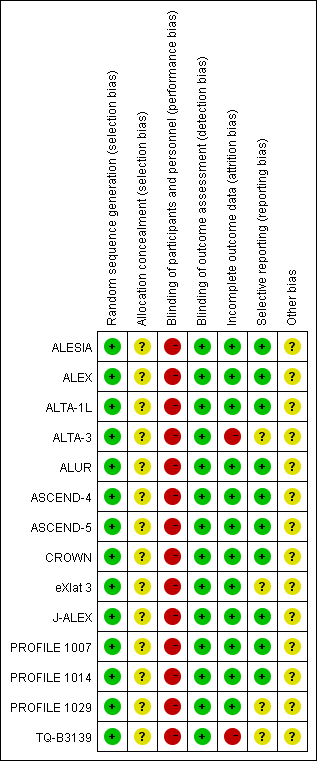
**

**
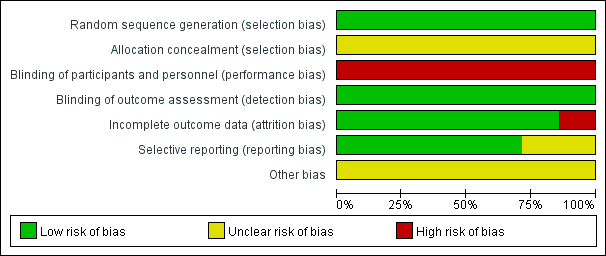
**

1. **Funnel plots**

1. Funnel plots in Network for for First-line Overall Survival


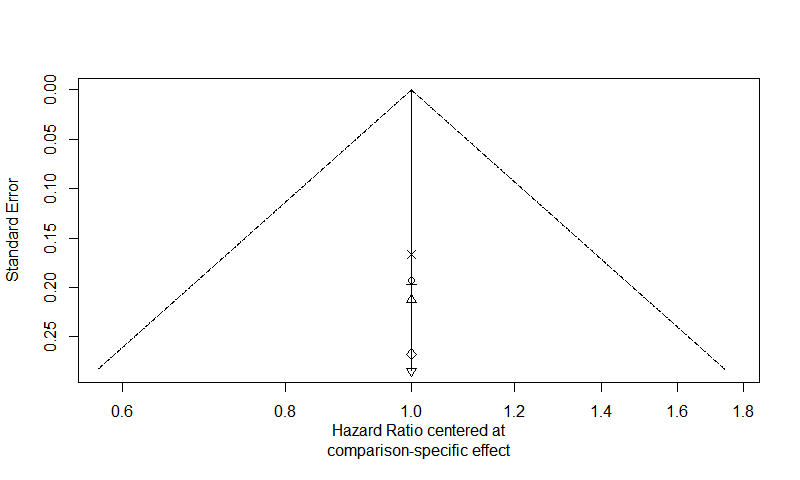


2. Funnel plots in Network for First-line Total Global Patients Based Systemic Progression-free Survival


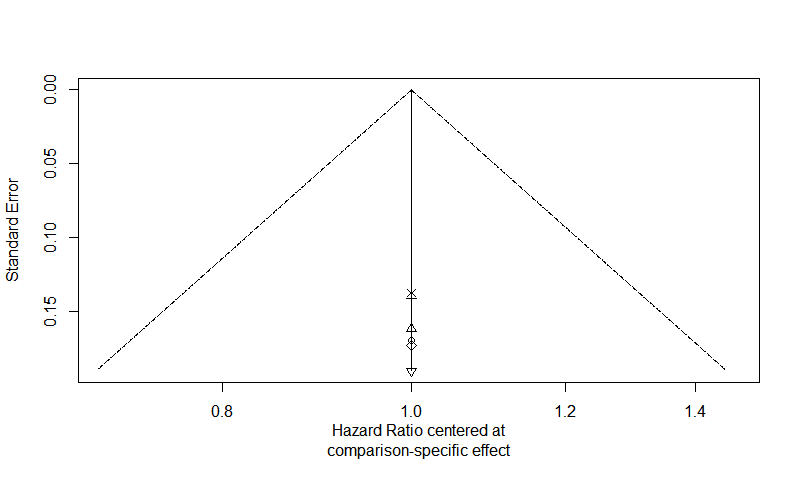


3. Funnel plots in Network for First-line Total Global Patients Central Nervous System Progression-free Survival


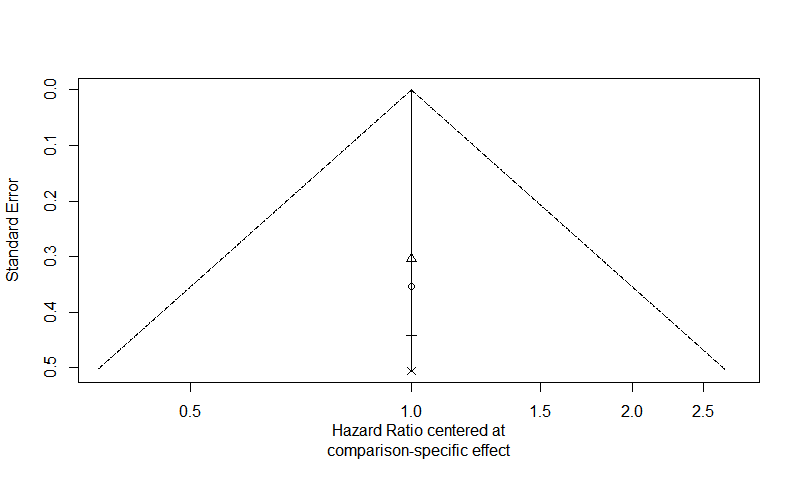


4. Funnel plots in Network for First-line Total Global Patients Non-Central Nervous System Progression-free Survival


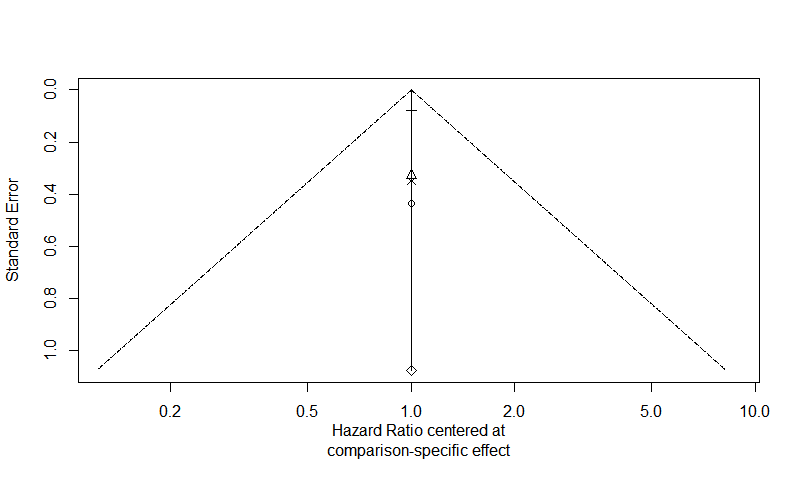


5. Funnel plots in Network for First-line Asian Patients Systemic Progression-free Survival


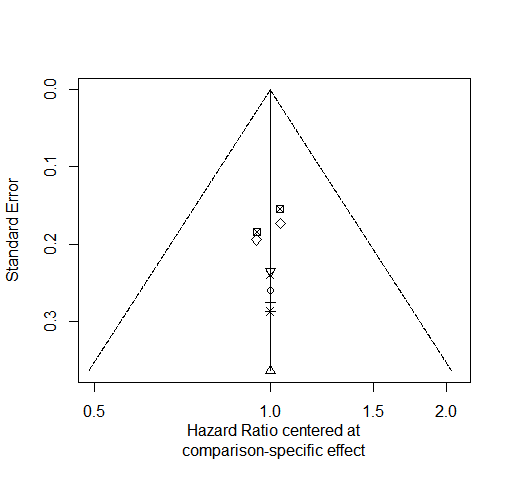


6. Funnel plots in Network for Second-line Patients Systemic Progression-free Survival


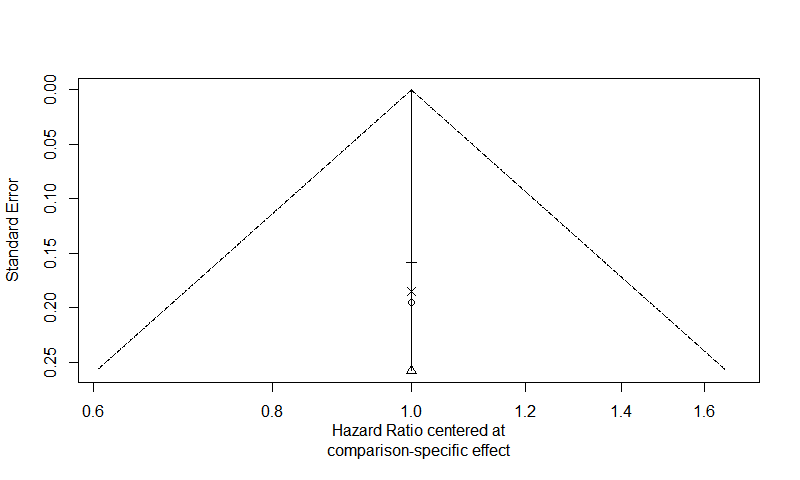


7. Funnel plots in Network for first-line Patients European Organisation for Research and Treatment of Cancer Quality of Life Questionnaire: lung cancer Module (EORTC QLQ-LC13)


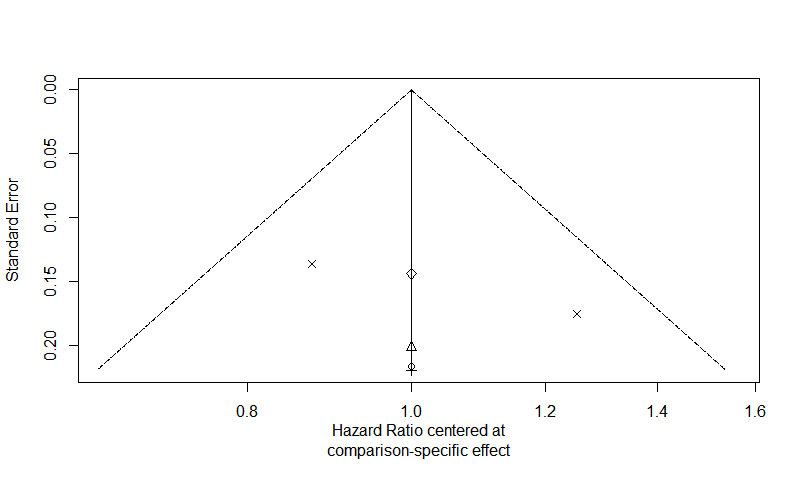


8. Funnel plots in Network for first-line Patients European Organisation for Research and Treatment of Cancer Quality of Life of Cancer Patients Questionnaire (EORTC) QLQ-C30 global quality of life


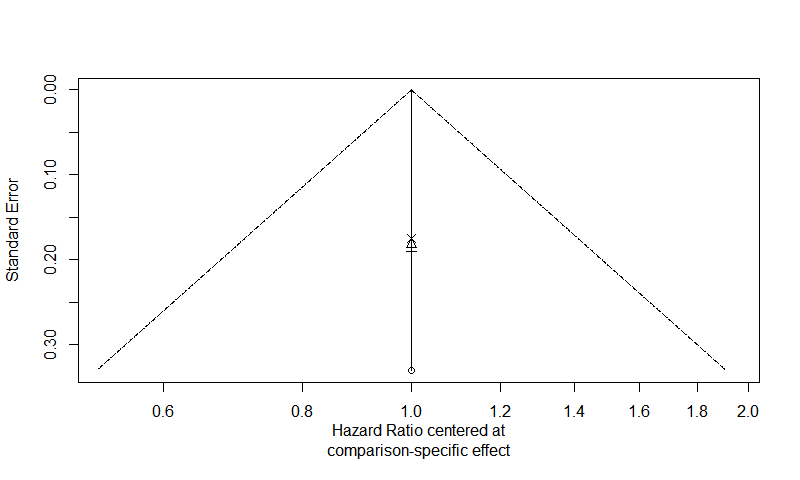

Supplement: Supplementary file 4 — Additional file 4. [file 12885_2024_11916_MOESM4_ESM.docx]
