# Supplementary material for: Identifying optimal ALK inhibitors in first- and second-line treatment of patients with advanced ALK-positive non-small-cell lung cancer: a systematic review and network meta-analysis
Source: BMC Cancer. 2024 Feb 8;24:186. doi: 10.1186/s12885-024-11916-4 (PMC10851546; doi:10.1186/s12885-024-11916-4)
Supplement: Supplementary file 5 — Additional file 5. [file 12885_2024_11916_MOESM5_ESM.docx]

**Additional file 5**

[eFigure 1 Study flow chart 2](#_Toc24204)

[eFigure 2A Network Plot 3](#_Toc15741)

[eFigure 2B Network Plot 4](#_Toc3212)

[eFigure 3 League table of RMST model 5](#_Toc107)

[eFigure 4 Subgroup and sensitivity analyses of OS 11](#_Toc29501)

[eFigure 5 Time-varying HR 12](#_Toc1144)

[eFigure 6 Ranking Plots 14](#_Toc21331)

[eFigure 7 Survival Curves based on RP model 16](#_Toc22605)

[eFigure 8 Summary Results of RMST 18](#_Toc18736)

[eFigure 9 Survival Curves based on FP models 19](#_Toc14788)

[eFigure 10 Forest plots for main subgroups based on Cox-PH model 20](#_Toc5695)

[eFigure 11 Forest plots for other subgroups based on Cox-PH model 21](#_Toc20055)

[eFigure 12 Summary Results of ORR 22](#_Toc29928)

[eFigure 13 League table of ORR 23](#_Toc11862)

[eFigure 14 League table of AE 26](#_Toc28058)

[eTable 1 Further baseline characteristics of included studies and patients 31](#_Toc21582)

[eTable 2 Grade 3+ adverse events (incidence over 5%) for treatments 33](#_Toc15651)

[eTable.3 Convergence and heterogeneity assessment 36](#_Toc19587)

[eTable 4 League table for Cox-PH model 38](#_Toc7864)

[eTable 5 EORTC QLQ-C30 Scores, Change From Baseline 44](#_Toc19789)

# eFigure 1 Study flow chart

Records identified through database search, n=1110

(Pubmed=475, EMbase=388, Cochrane library=95, clinical trials.gov=47, ESMO/ASCO/WCLC=105)

(n =1032)

Excluded for duplicates or blank records

(n =141)

Titles and abstracts screening

(n =969)

Reasons for excluded full-text articles

(n =151)

- Non-target population (n=86)

- No Kaplan-Meier Curves or target results (n =65)

Full-text articles screening

(n =165)

Reasons for excluded titles and abstracts (n=804)

- Single arm trial (n =80)

- No corresponding topics (n=584)

- Observational study or case reports (n =77)

- Secondary analysis or study protocol (n =63)

Total articles used for data extraction

(n =14)

**Identification**

**Screening**

**Eligibility**

**Inclusion**

# eFigure 2A Network Plot

(A. For OS And Systemic PFS of Global Patients and brain metastasis- or Non-brain metastasis Subgroup Systemic PFS of Global Patients; B. For intracranial PFS of Global Baseline Brain Metastasis Patients; C. For intracranial PFS of Global Baseline No Brain Metastasis Patients; D. For Systemic PFS of Asian Patients; E. For Systemic PFS of Previously Crizotinib-treated Patients; F. For QLQ-LC13 of Global Patients; G. For QLQ-C30 of Global Patients)


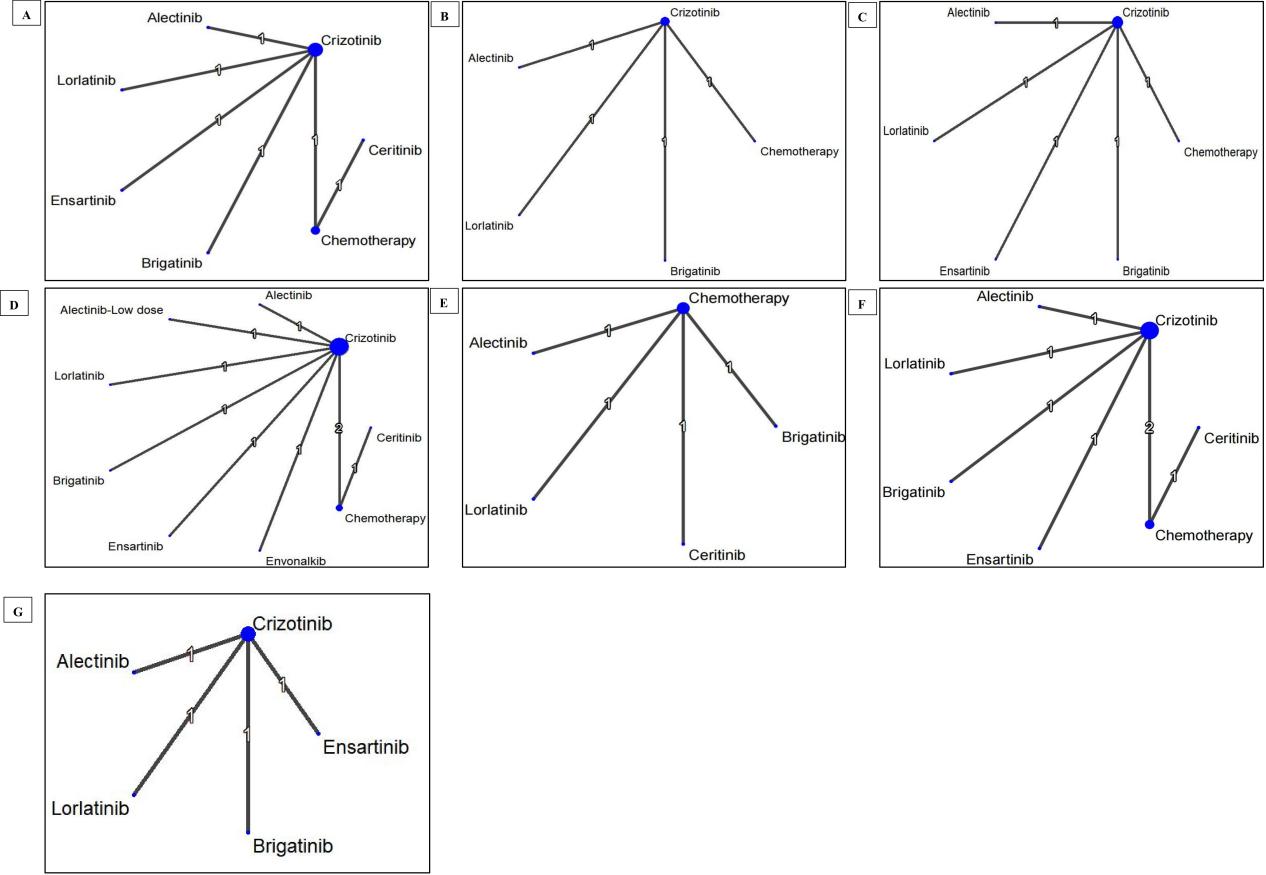


# eFigure 2B Network Plot

(A. Overall ORR for first-line treatments; B. intracranial ORR for first-line treatments; C. Overall ORR for second-line treatments; D. intracranial ORR for second-line treatments; E. Any-grade AE for first-line treatments; F. Any-grade AE for second-line treatments; G. Grade 3-4 AE for first-line treatments; H. Grade 3-4 AE for first-line treatments; I. Grade 5 AE for first-line treatments)


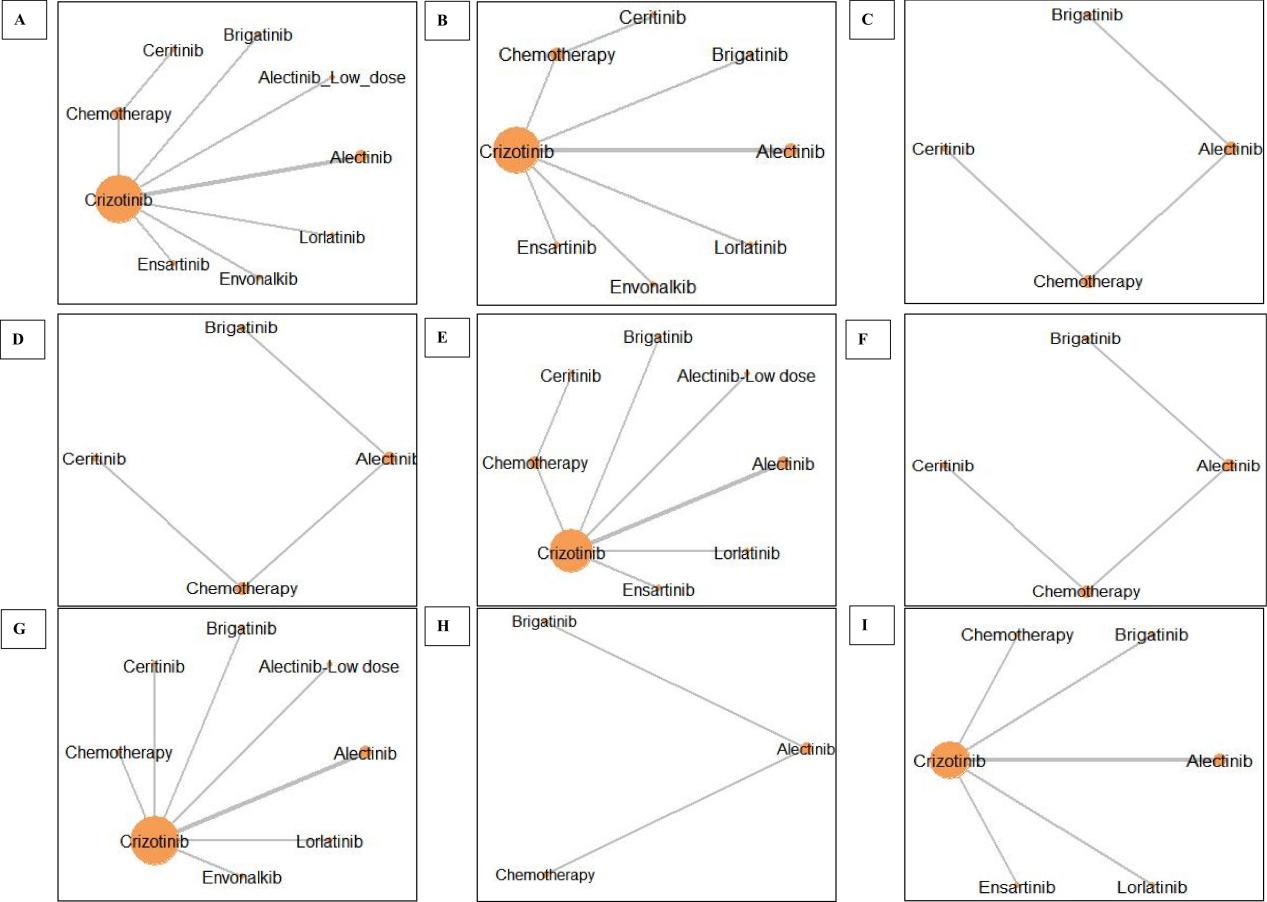


# eFigure 3 League table of RMST model

(A. OS of First-line Treatments; B. PFS of First-line Treatments; C. PFS of First-line Treatments on Asian Patients; D. PFS of Second-line Treatments; E. Intracranial PFS of First-line Treatments for Baseline Brain Metastasis Patients; F. Intracranial PFS of First-line Treatments for Baseline No Brain Metastasis Patients; G. PFS of First-line Treatments in brain metastasis subgroup; H. PFS of First-line Treatments in non-brain metastasis subgroup; I. QLQ-LC13 of First-line Treatments; J, QLQ-C30 for first-line treatments)

A. OS of First-line Treatments


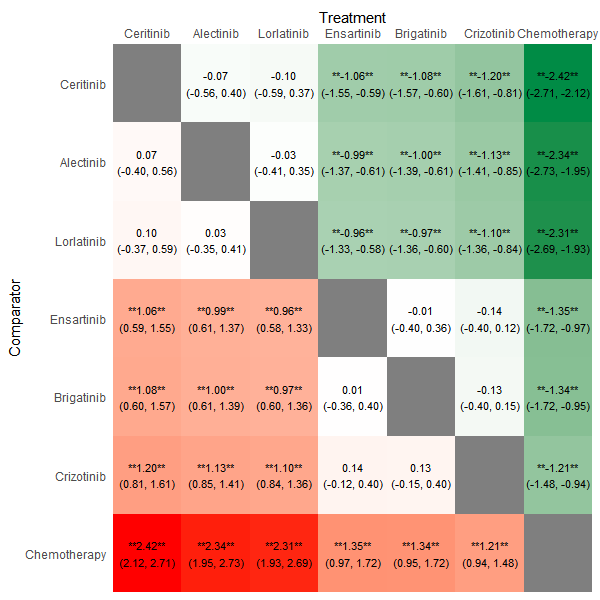


# B. PFS of First-line Treatments

#
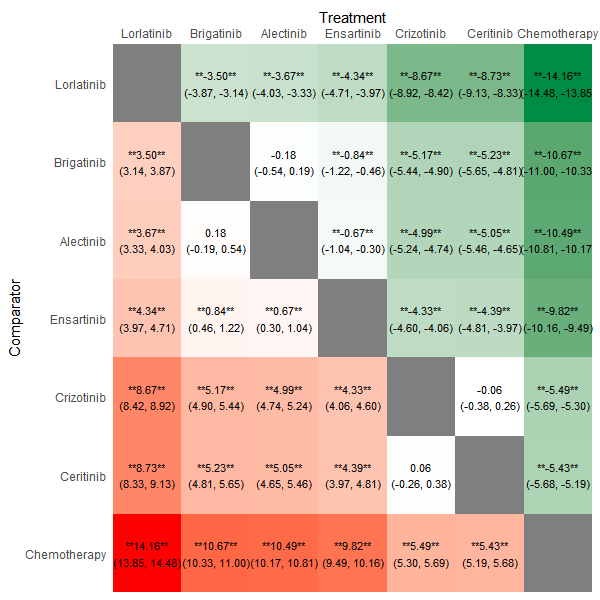


C. PFS of First-line Treatments on Asian Patients


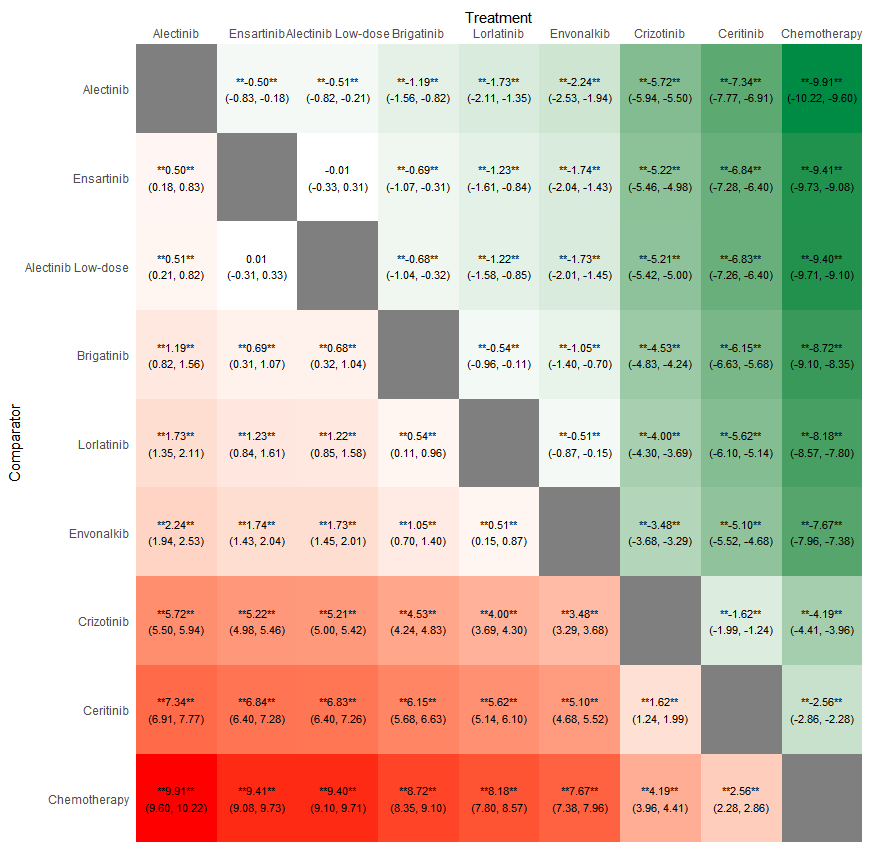


D. PFS of Second-line Treatments


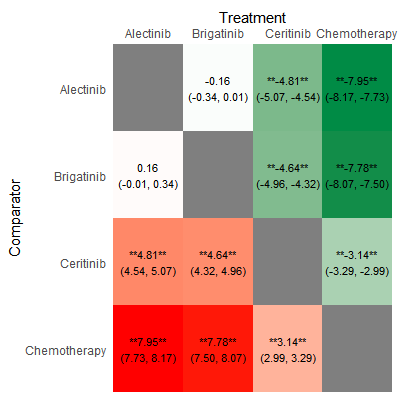


E. Intracranial PFS of First-line Treatments for Baseline Brain Metastasis Patients


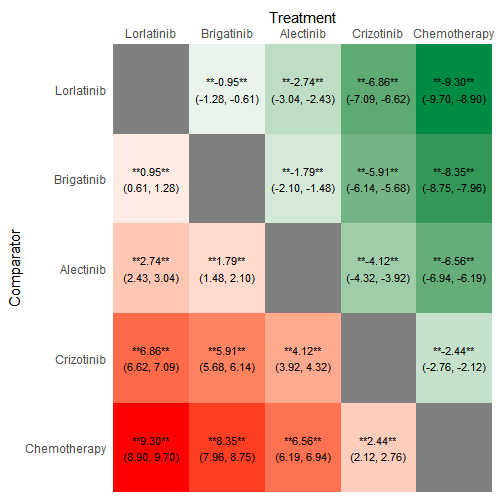


F. Intracranial PFS of First-line Treatments for Baseline No Brain Metastasis Patients


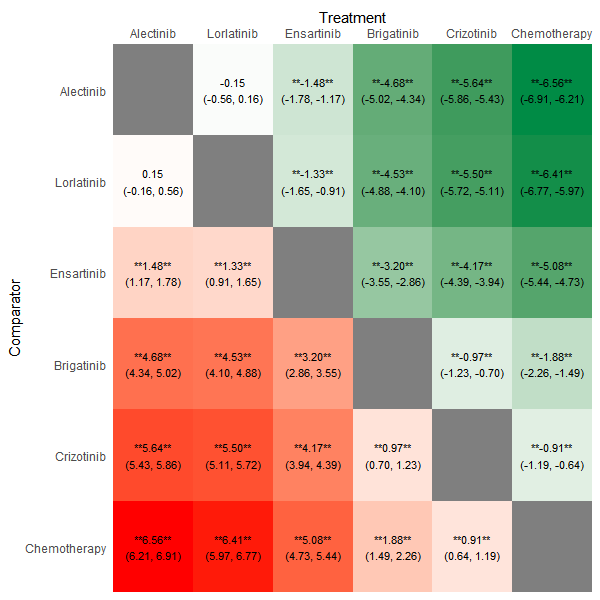


G. PFS of First-line Treatments in Brain Metastasis Subgroup


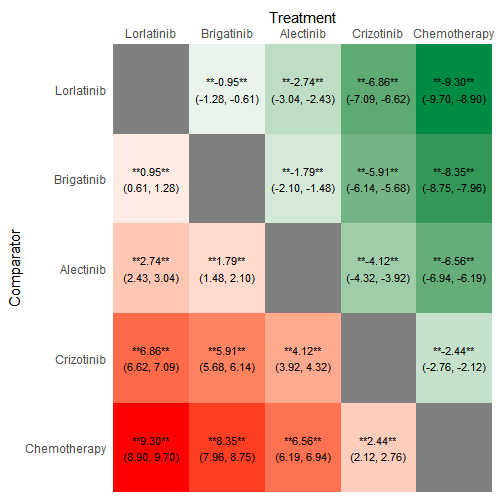


H. PFS of First-line Treatments in no brain metastasis subgroup


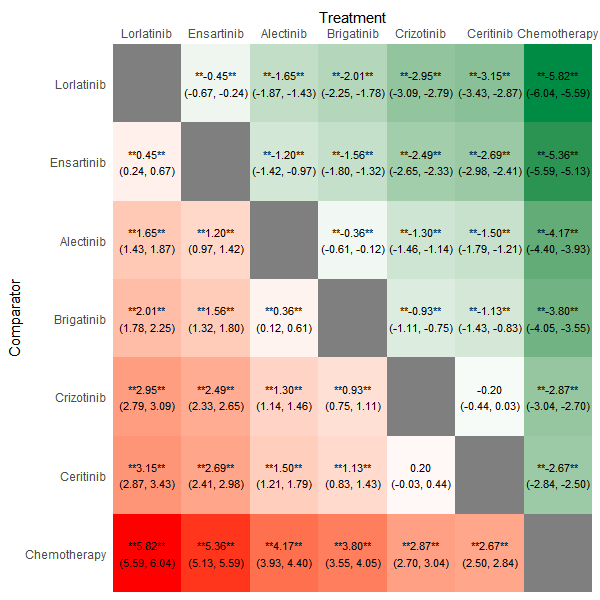


I. QLQ-LC13 of First-line Treatments


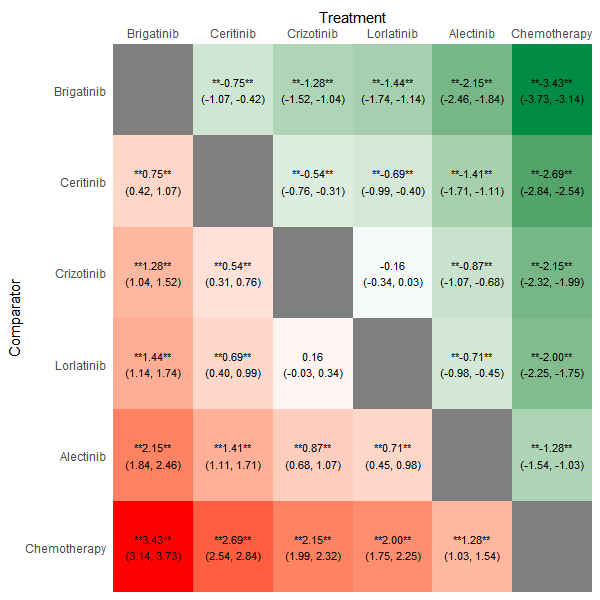


J. QLQ-C30 for first-line treatments


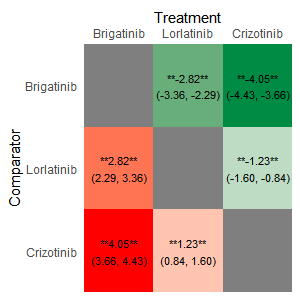


*significant difference

eFigure 4 Subgroup and sensitivity analyses of OS (A. with baseline brain metastasis; B. without baseline brain metastasis; C. results for trials that did not permit crossover; D. results for trials that did permit crossover)

| A | B |
| --- | --- |
| 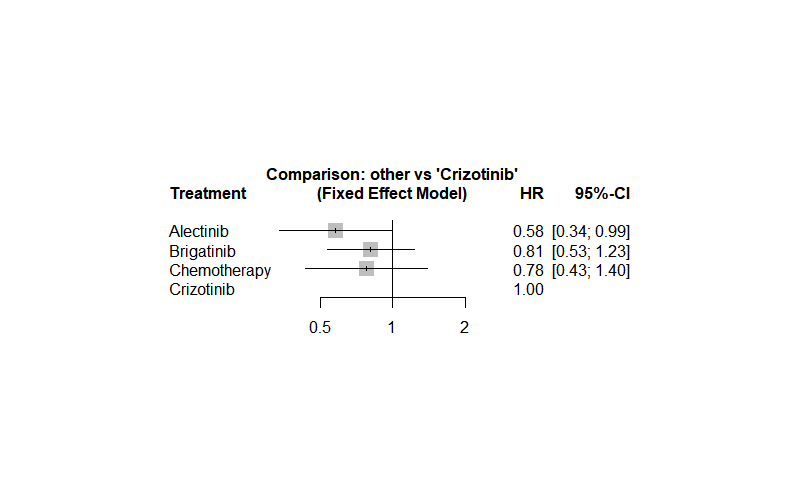 | 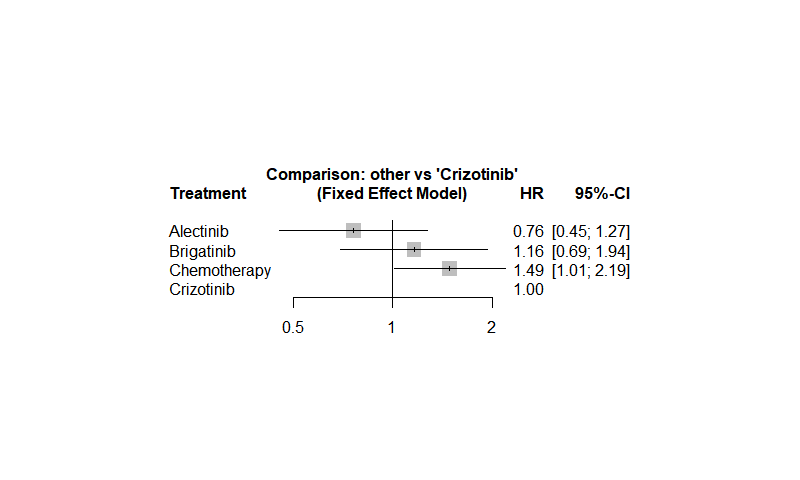 |
| C | D |
| 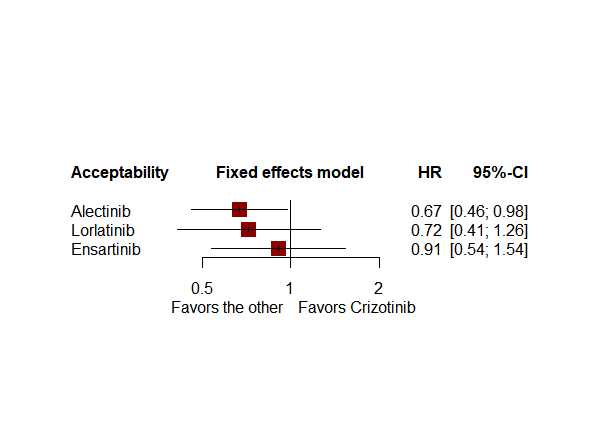 | 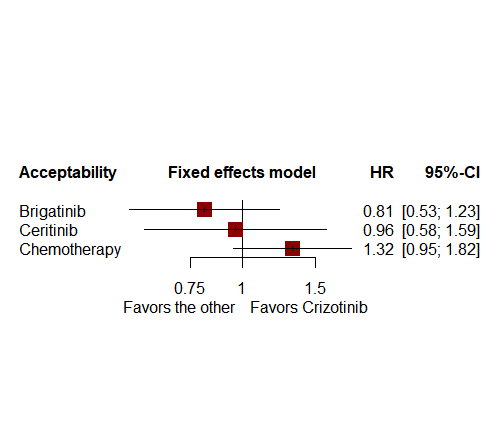 |

# eFigure 5 Time-varying HR

(A. OS of First-line Treatments; B. PFS of First-line Treatments; C. PFS of First-line Treatments on Asian Patients; D. PFS of Second-line Treatments; E. intracranial PFS of First-line Treatments for Baseline Brain Metastasis Patients; F. intracranial PFS of First-line Treatments for Baseline No Brain Metastasis Patients; G, PFS of First-line Treatments in brain metastasis subgroup; H PFS of First-line Treatments in non-brain metastasis subgroup; I, QLQ-LC13 of First-line Treatments; J, QLQ-C30 for first-line treatments)

| A | B |
| --- | --- |
| 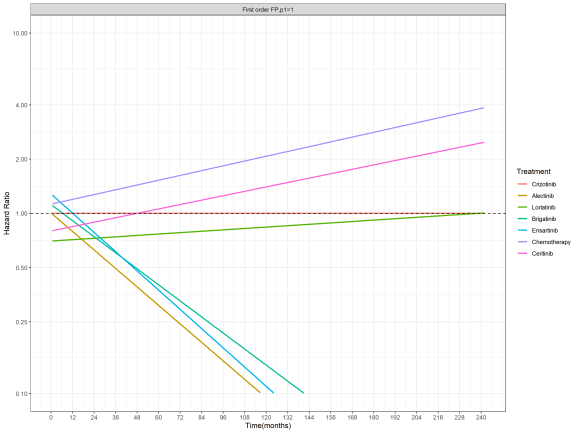 | 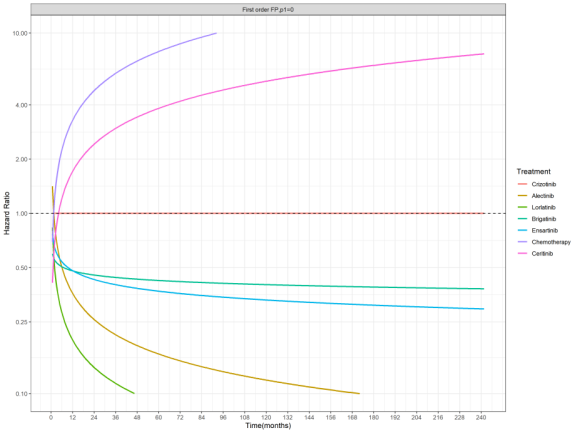 |
| C | D |
| 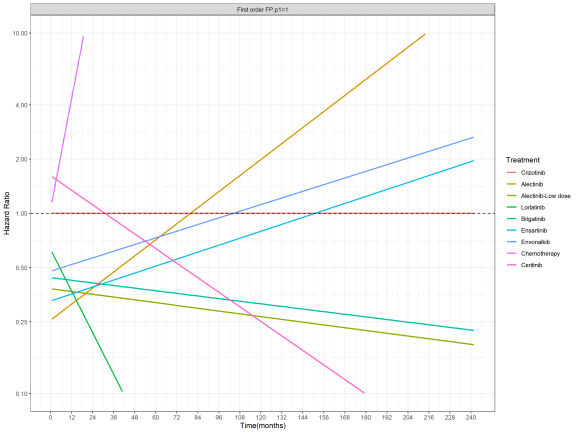 | 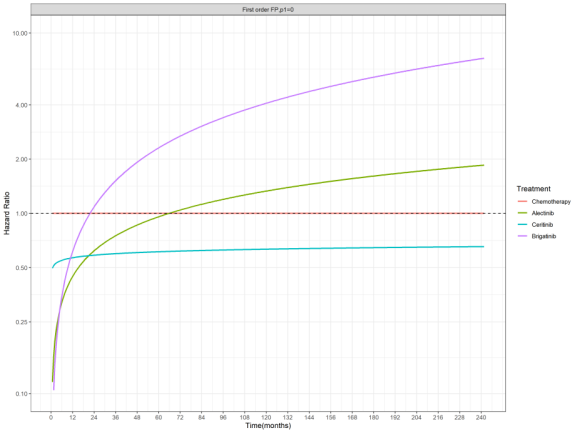 |
| E | F |
| 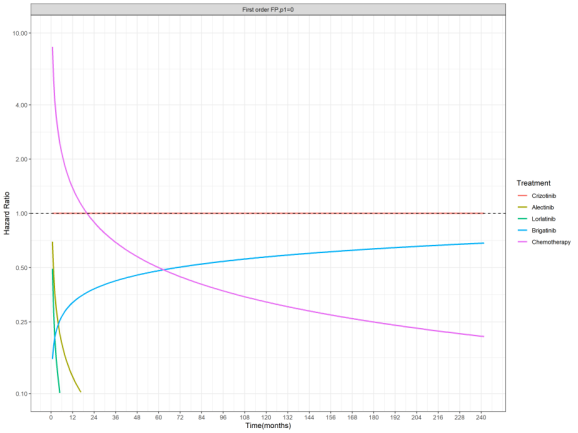 | 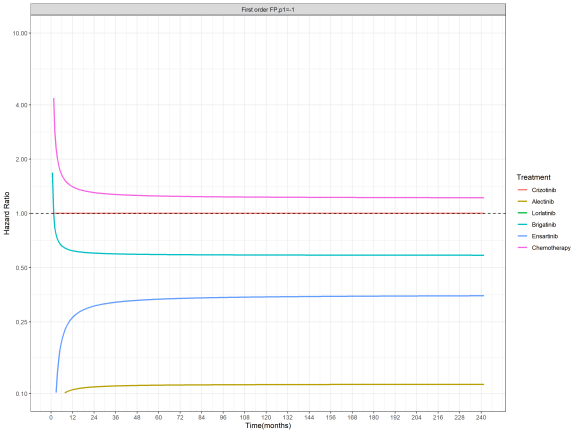 |
| G | H |
| 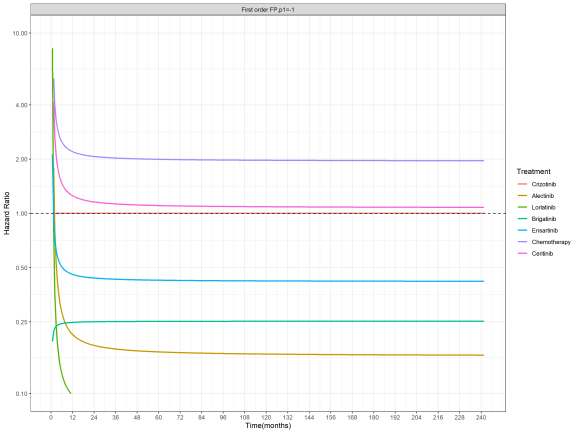 | 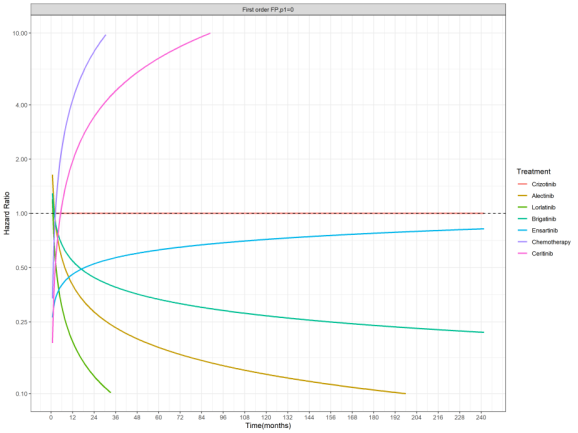 |
| I | J |
| 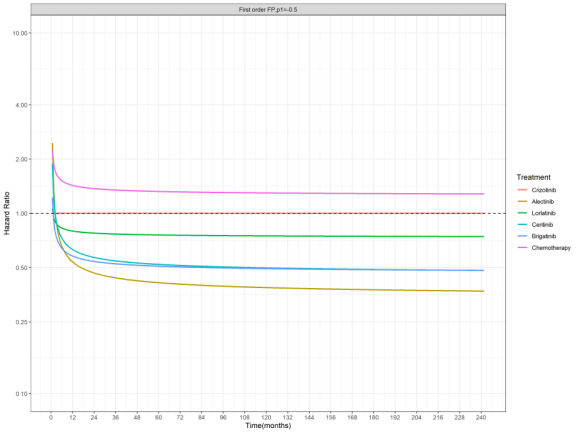 | 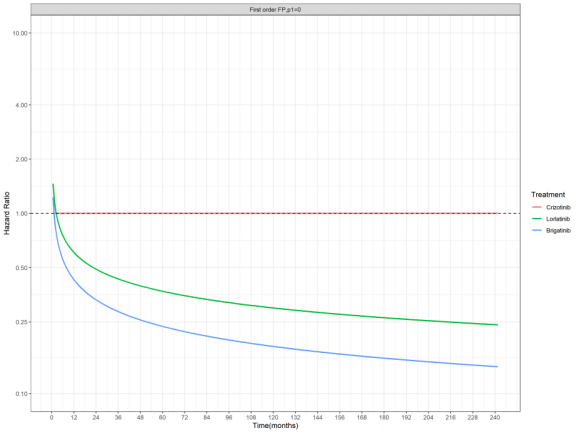 |

# eFigure 6 Ranking Plots

(A. PFS of First-line Treatments; B. PFS of First-line Treatments on Asian Patients; C. OS of First-line Treatments; D. intracranial PFS of First-line Treatments for Baseline Brain Metastasis Patients; E. intracranial PFS of First-line Treatments for Baseline No Brain Metastasis Patients; F, PFS of First-line Treatments in brain metastasis subgroup; G, PFS of First-line Treatments in non-brain metastasis subgroup; H. PFS of Second-line Treatments; I, QLQ-LC13 of First-line Treatments; J, QLQ-C30 for first-line treatments)

| A | B |
| --- | --- |
| 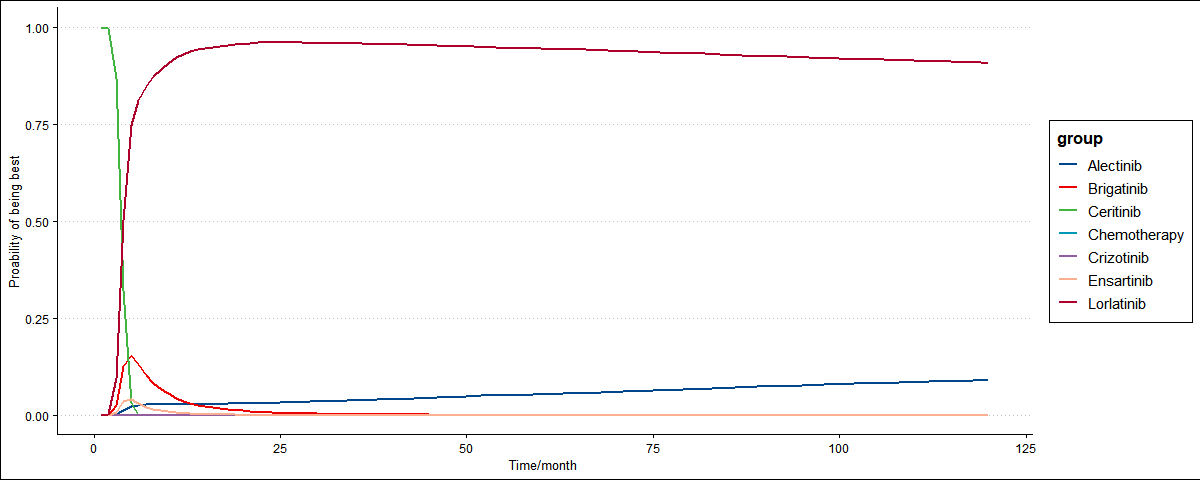 | 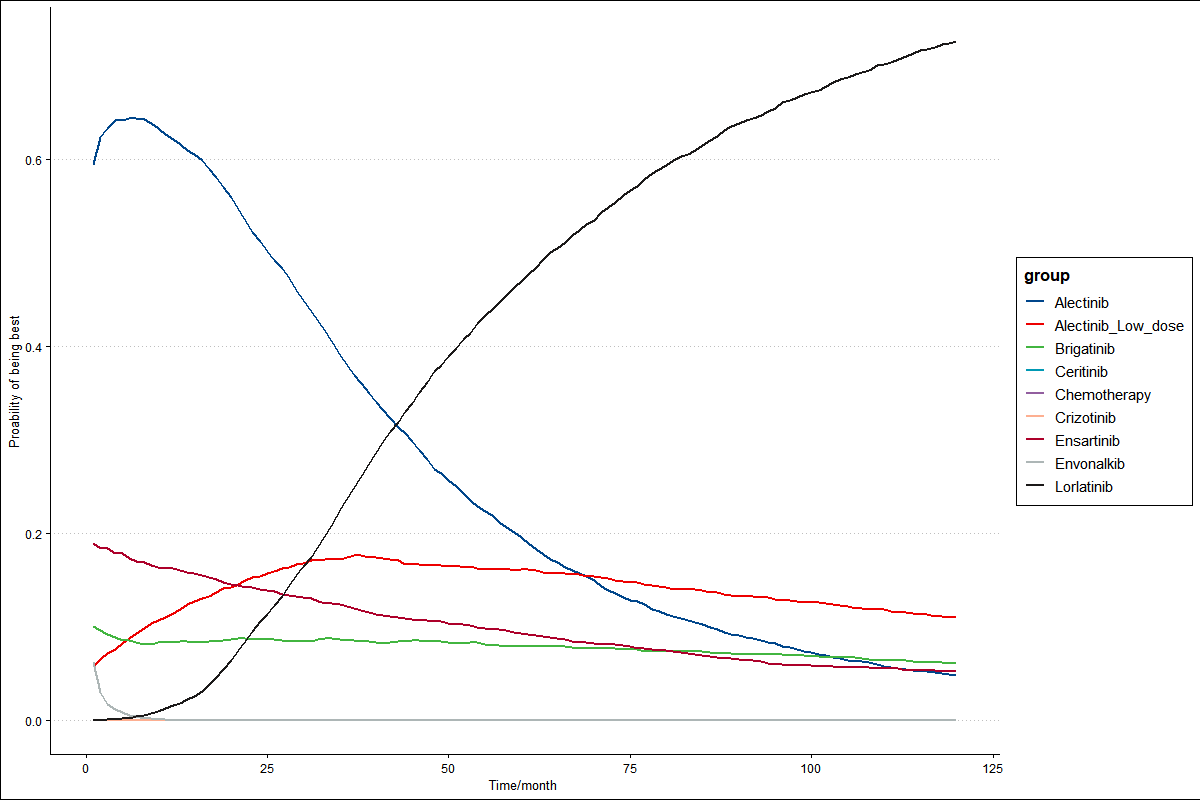 |
| C | D |
| 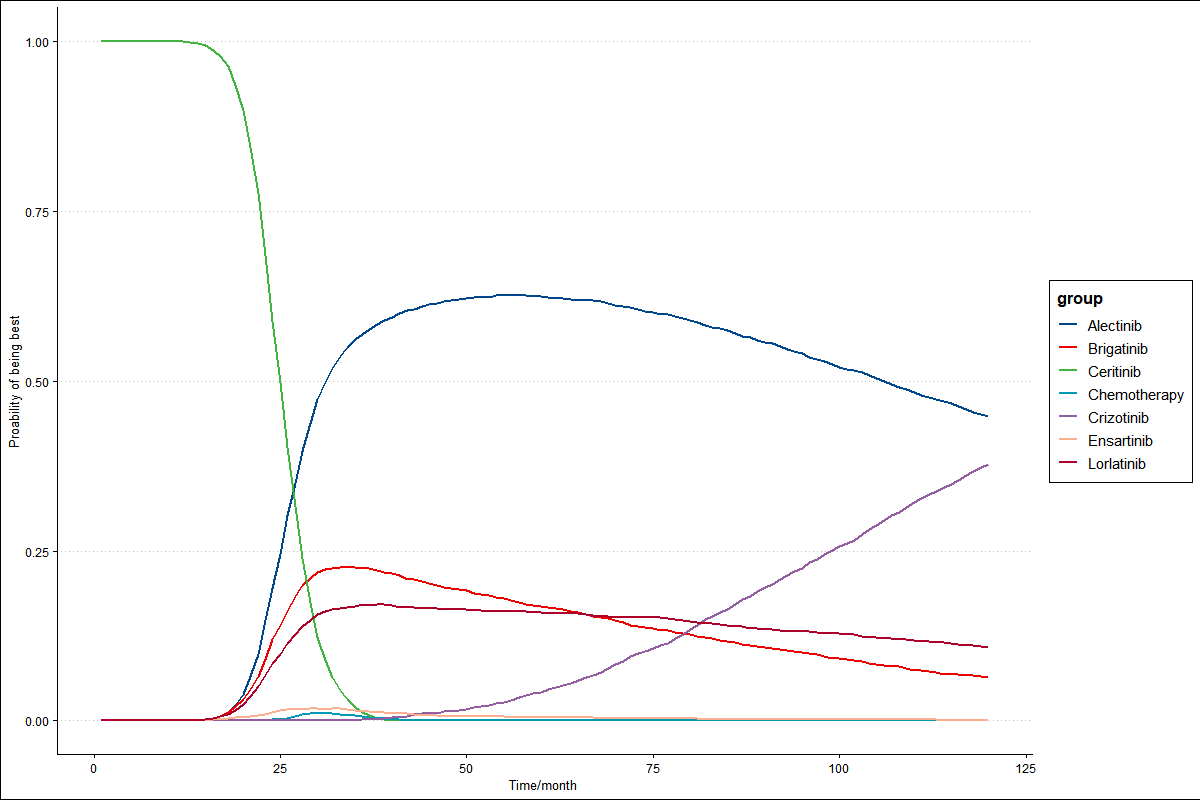 | 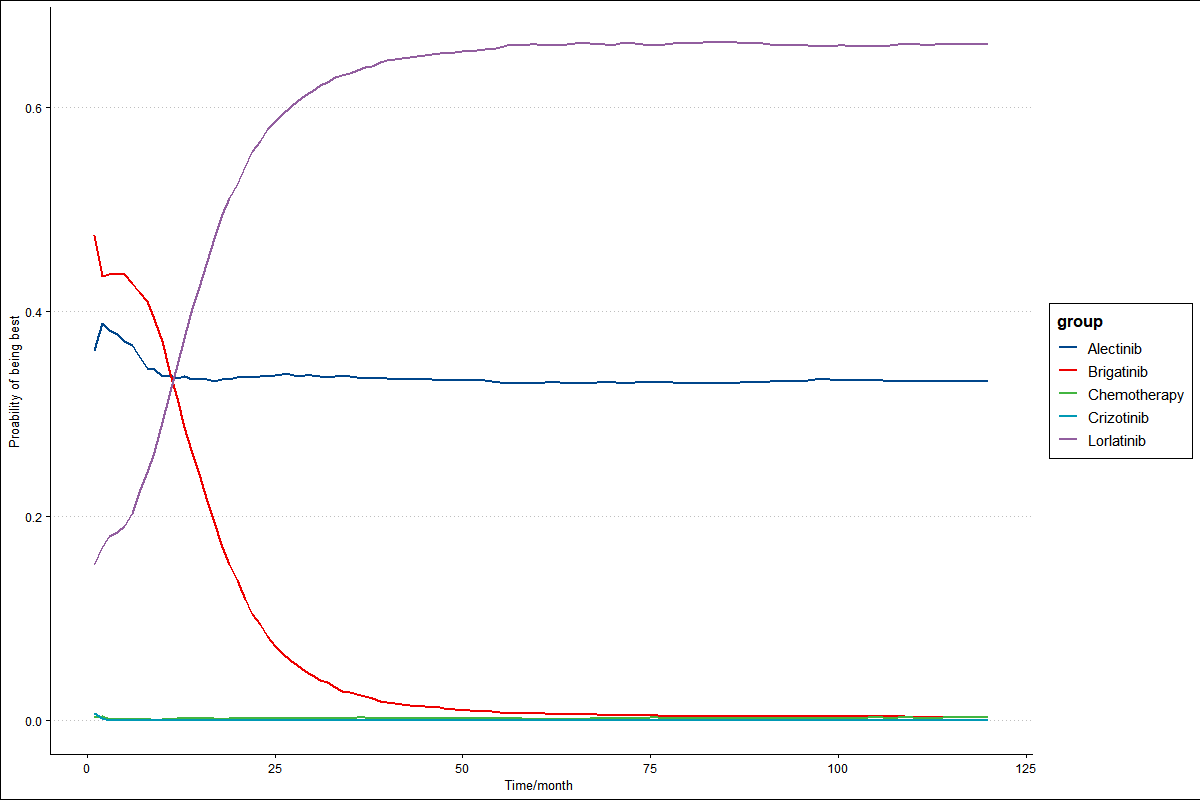 |
| E | F |
| 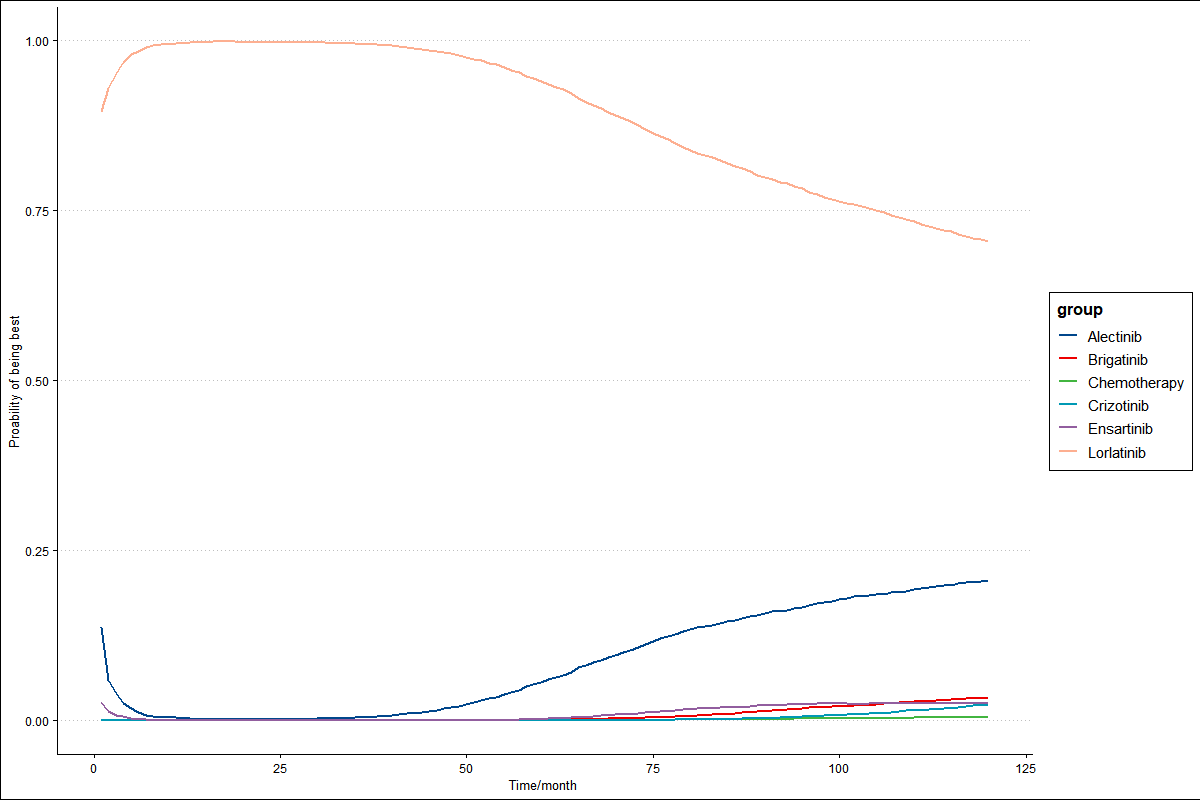 | 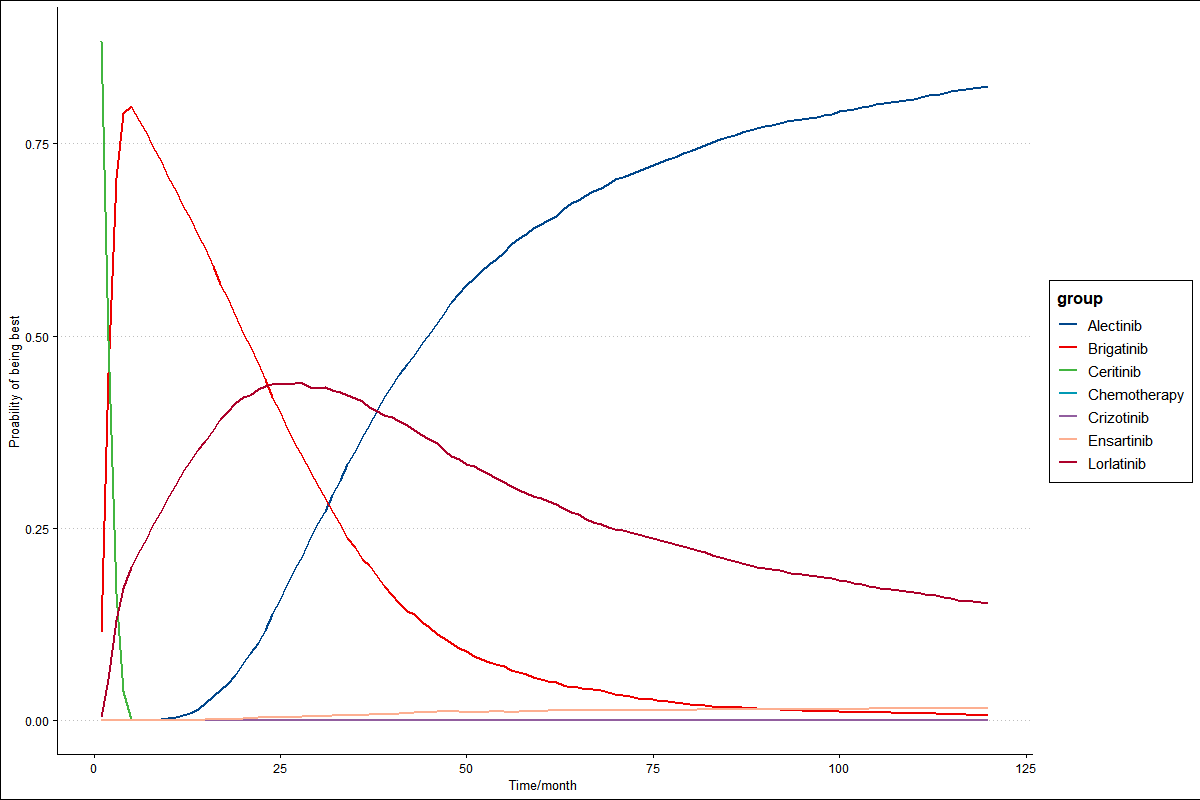 |
| G | H |
| 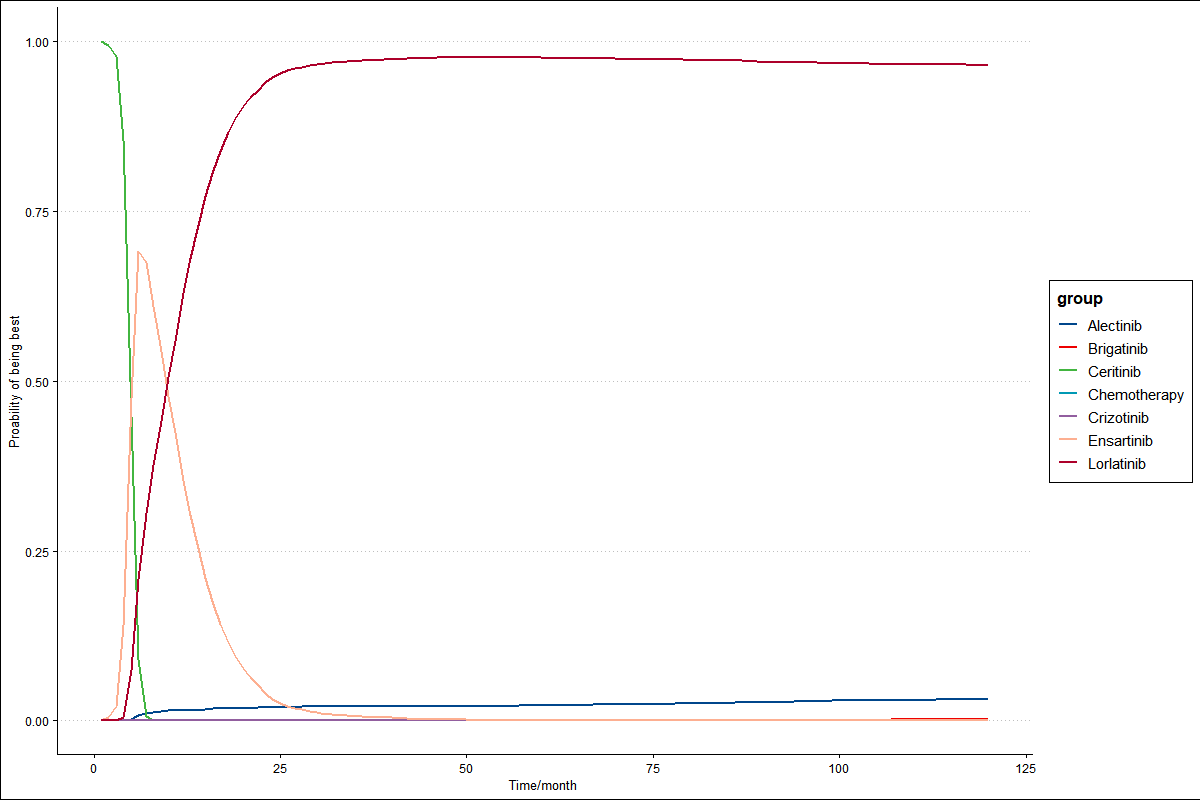 | 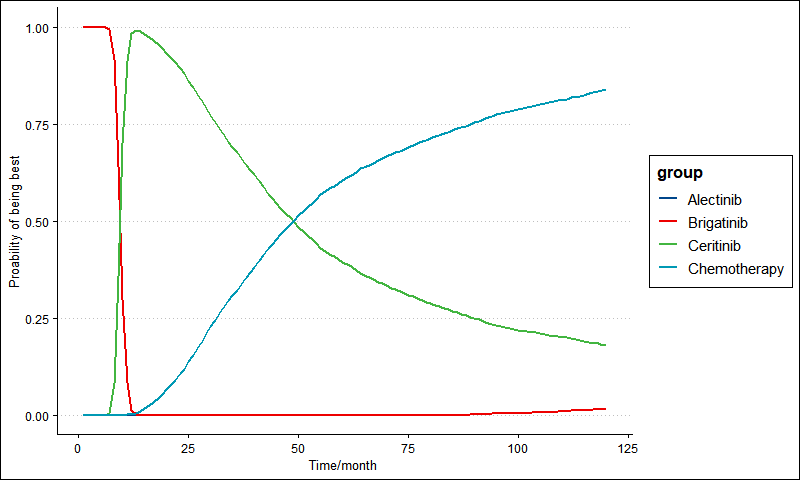 |
| I | J |
| 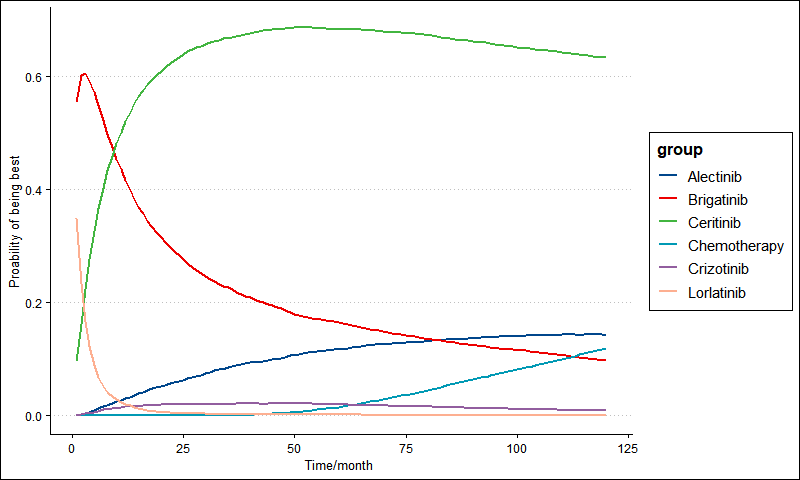 | 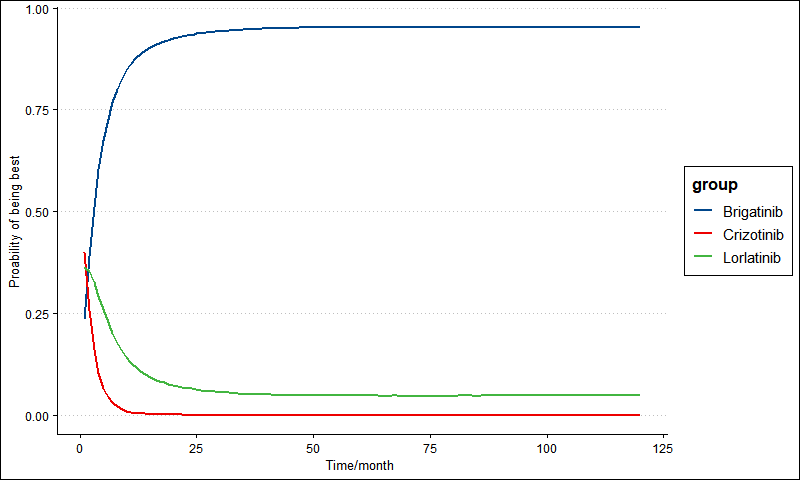 |

# eFigure 7 Survival Curves based on RP model

(A. PFS of First-line Treatments; B. PFS of First-line Treatments on Asian Patients; C. OS of First-line Treatments; D. intracranial PFS of First-line Treatments for Baseline Brain Metastasis Patients; E, PFS of First-line Treatments in brain metastasis subgroup; F. intracranial PFS of First-line Treatments for Baseline No Brain Metastasis Patients; G, PFS of First-line Treatments in non-brain metastasis subgroup; H. PFS of Second-line Treatments; I, QLQ-LC13 of First-line Treatments; J, QLQ-C30 for first-line treatments)

| A | B |
| --- | --- |
| 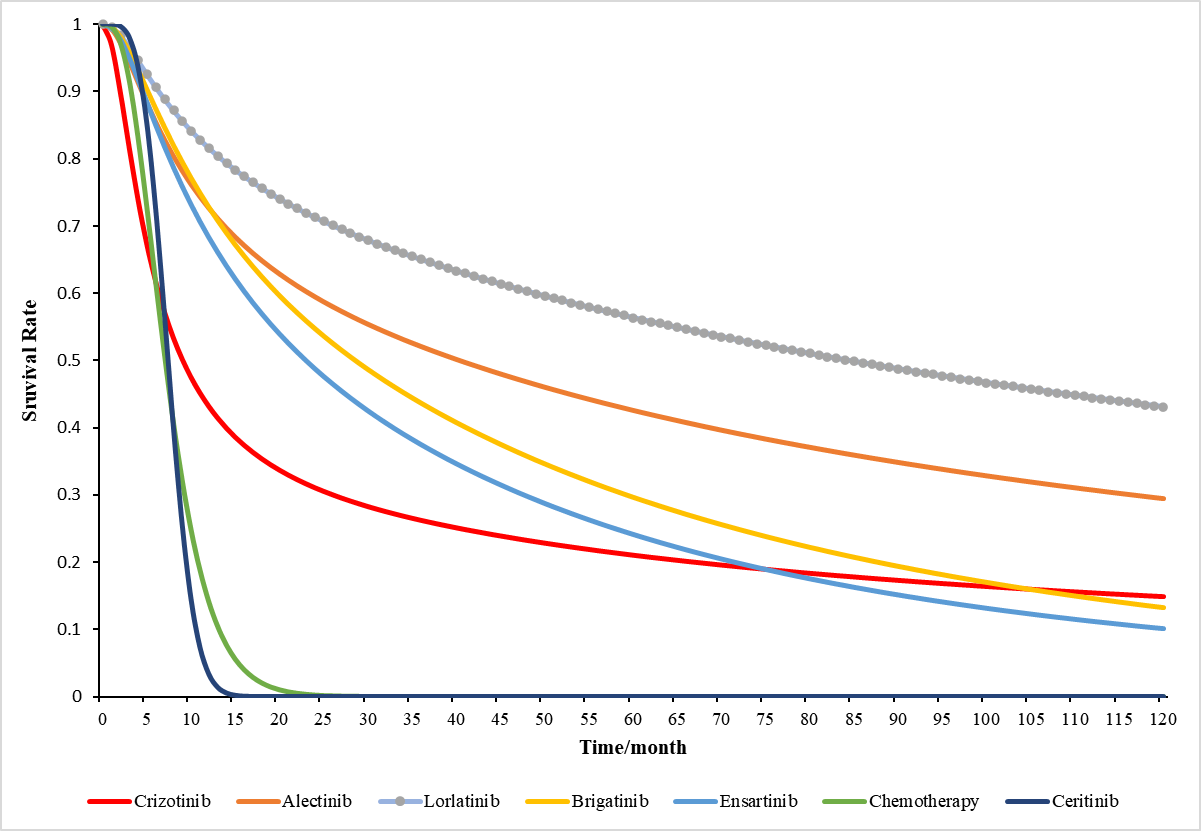 | 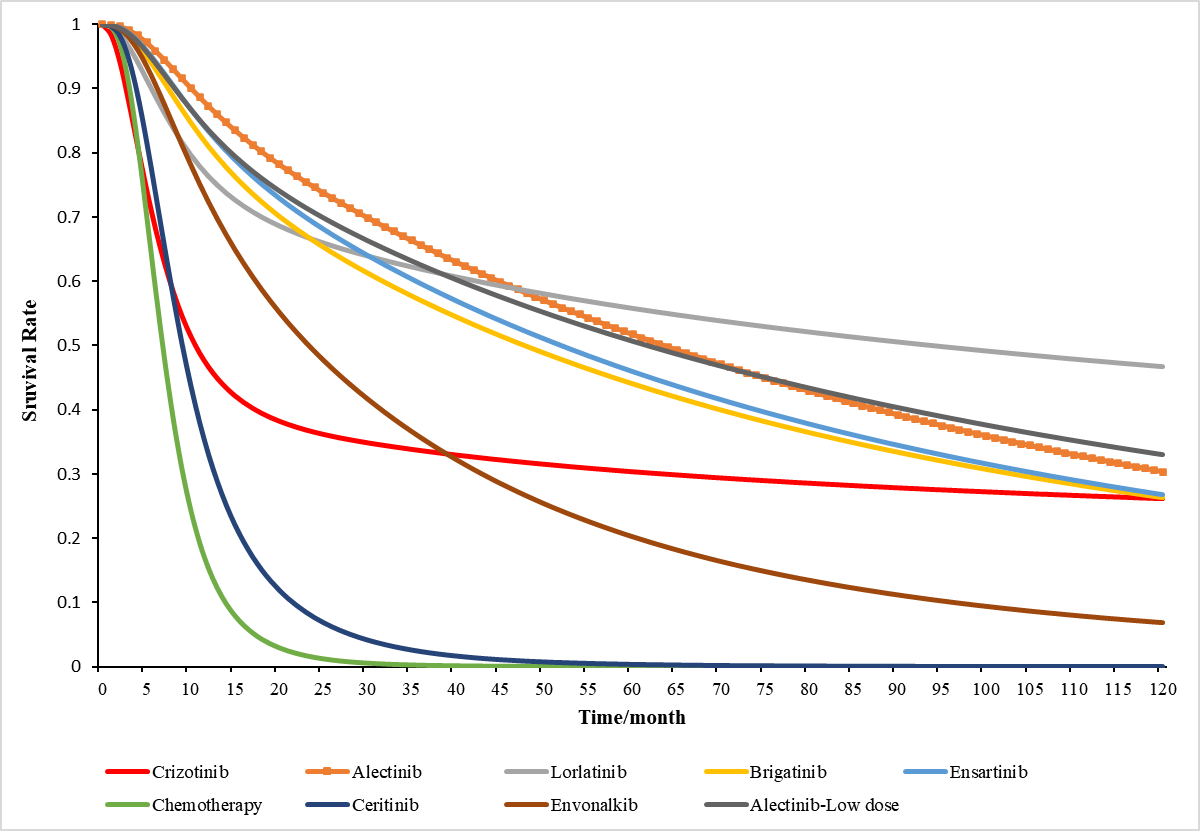 |
| C | D |
| 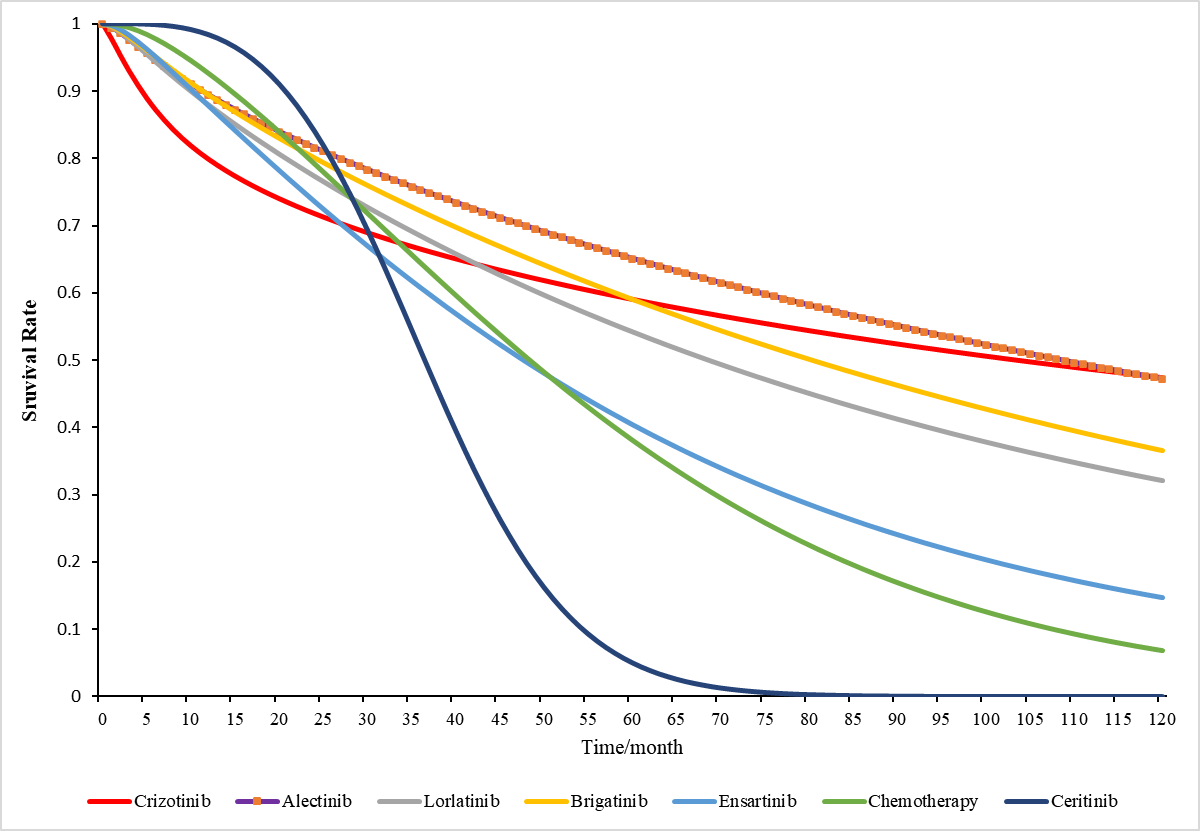 | 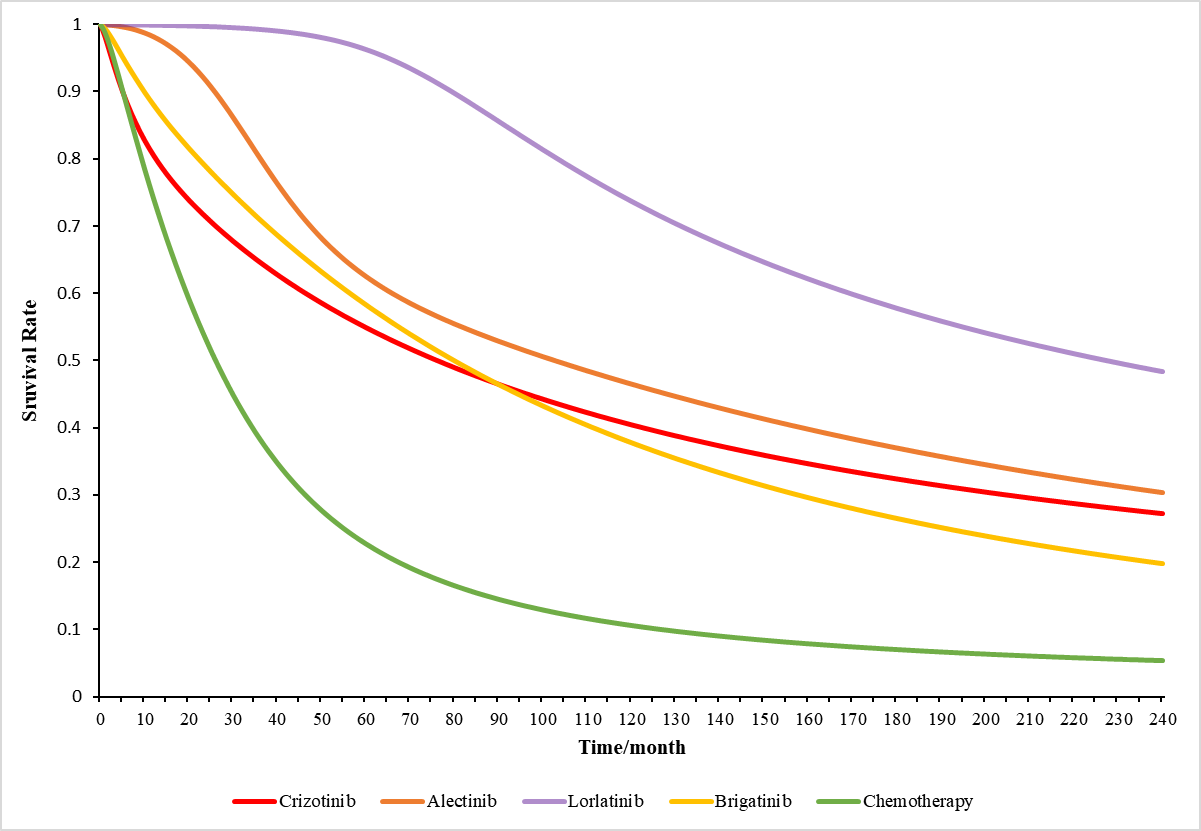 |
| E | F |
| 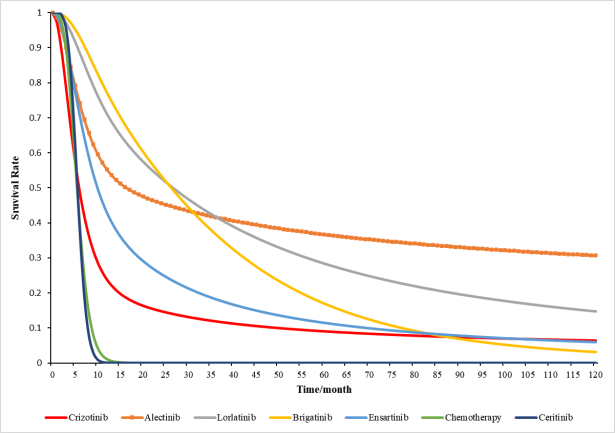 | 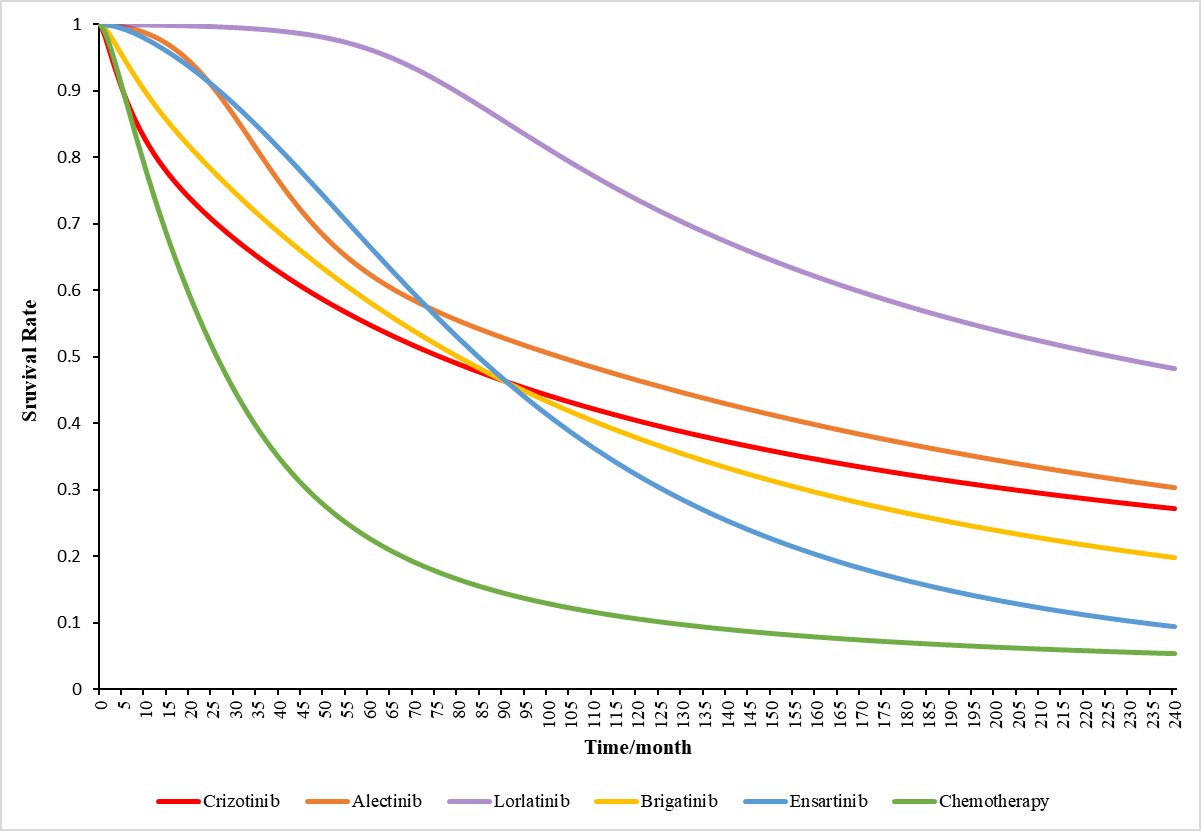 |
| G | H |
| 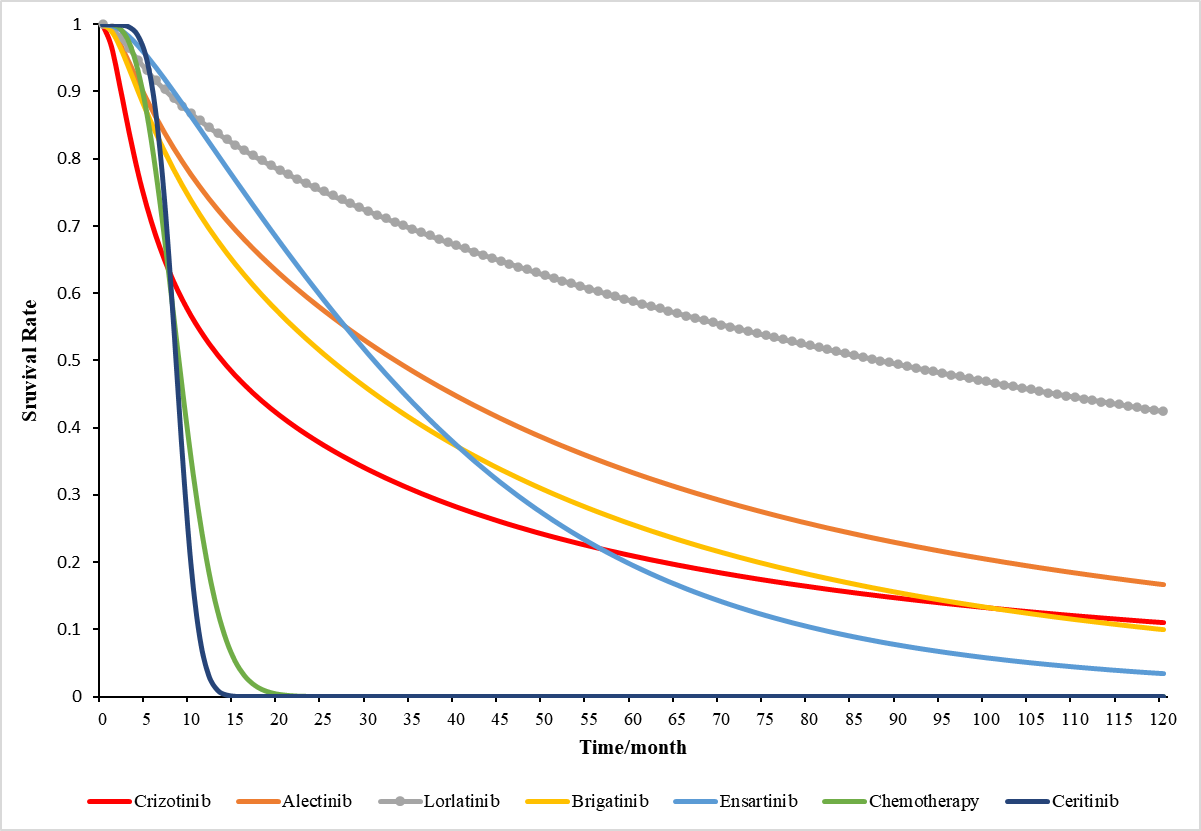 | 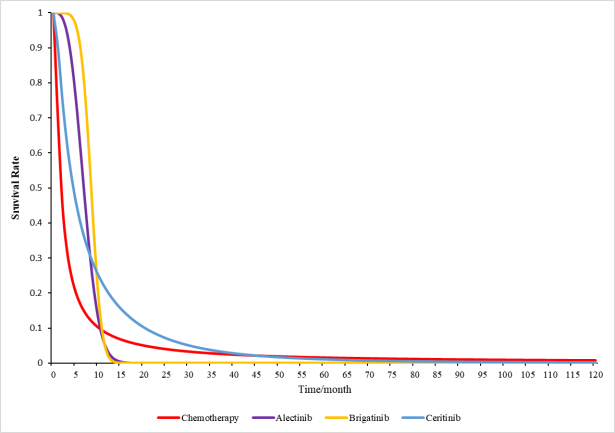 |
| I | J |
| 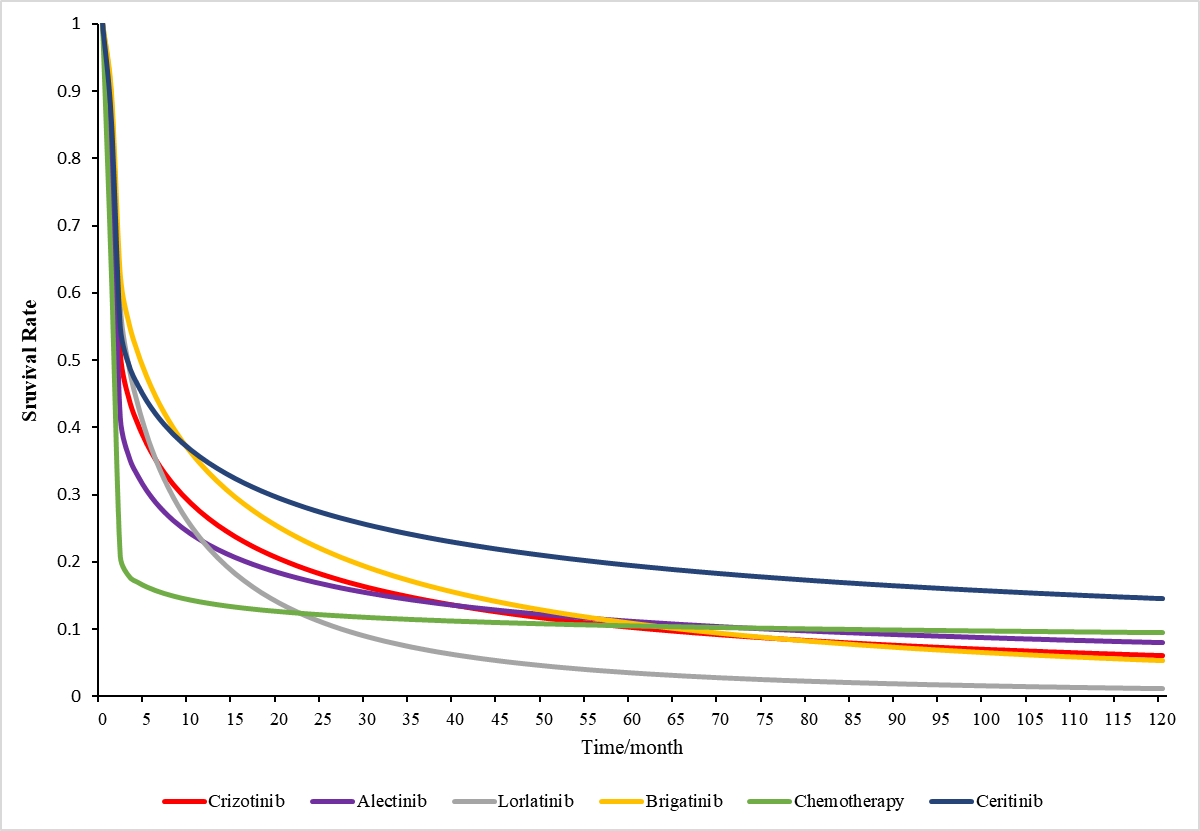 | 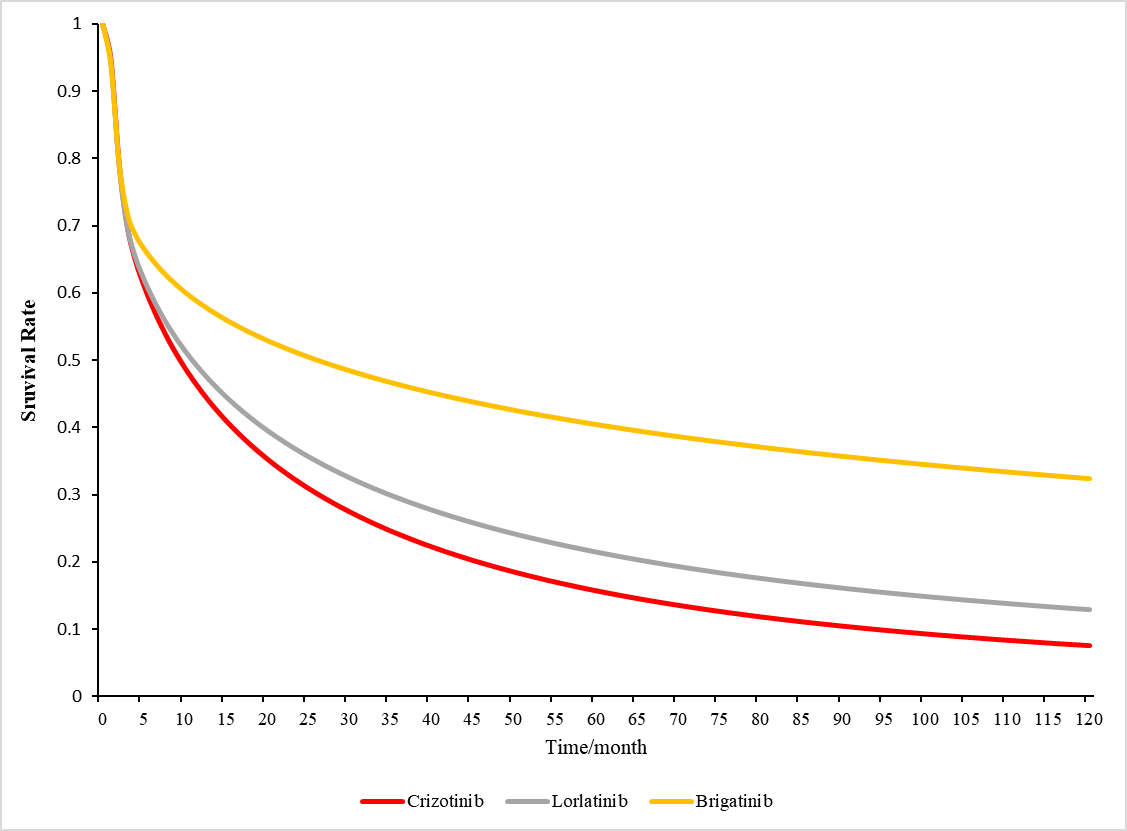 |

# eFigure 8 Summary Results of RMST

(1, PFS of First-line Treatments in brain metastasis subgroup; 2, PFS of First-line Treatments in non-brain metastasis subgroup; 3, QLQ-LC13 of First-line Treatments; 4, QLQ-C30 for first-line treatments)


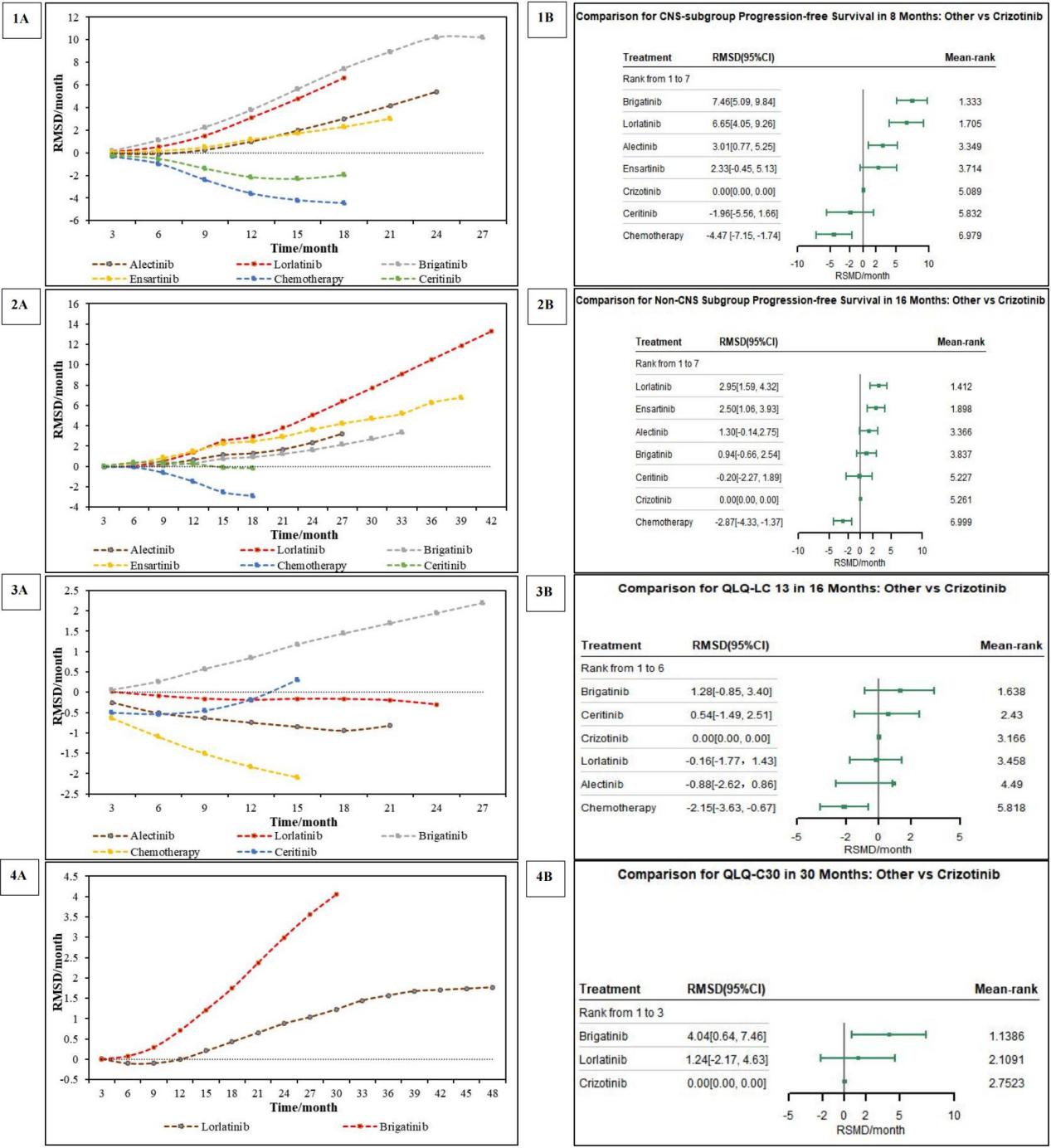


# eFigure 9 Survival Curves based on FP models

(A, PFS of First-line Treatments in brain metastasis subgroup; B, PFS of First-line Treatments in non-brain metastasis subgroup; C, QLQ-LC13 of First-line Treatments; D, QLQ-C30 for first-line treatments)

| A | B |
| --- | --- |
| 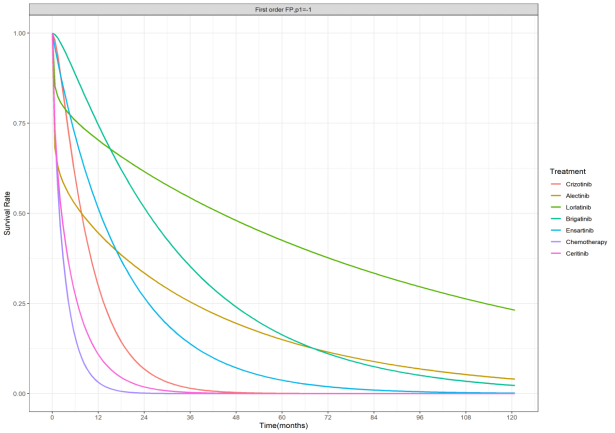 | 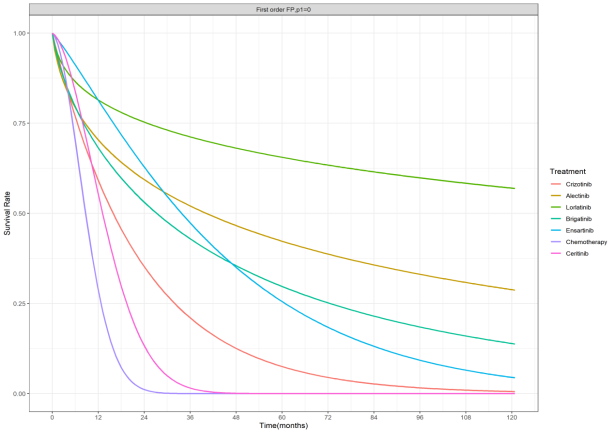 |
| C | D |
| 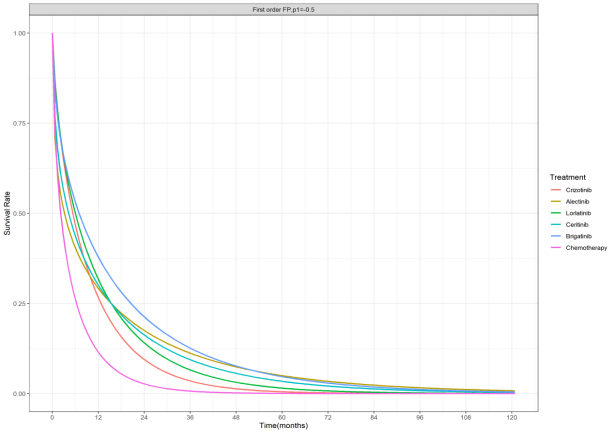 | 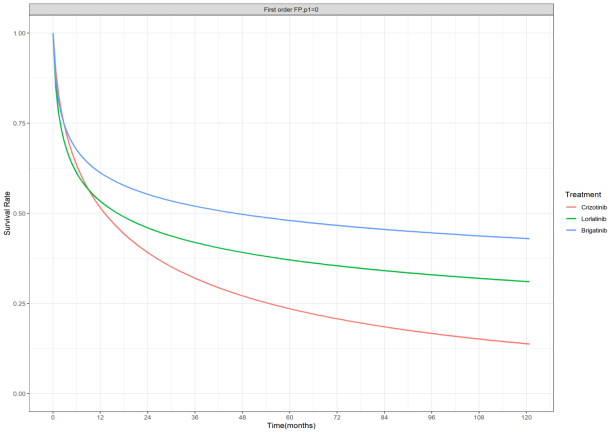 |

# eFigure 10 Forest plots for main subgroups based on Cox-PH model

(A, PFS of First-line Treatments in brain metastasis subgroup; B, PFS of First-line Treatments in non-brain metastasis subgroup; C, QLQ-LC13 of First-line Treatments; D, QLQ-C30 for first-line treatments)

| A | B |
| --- | --- |
| 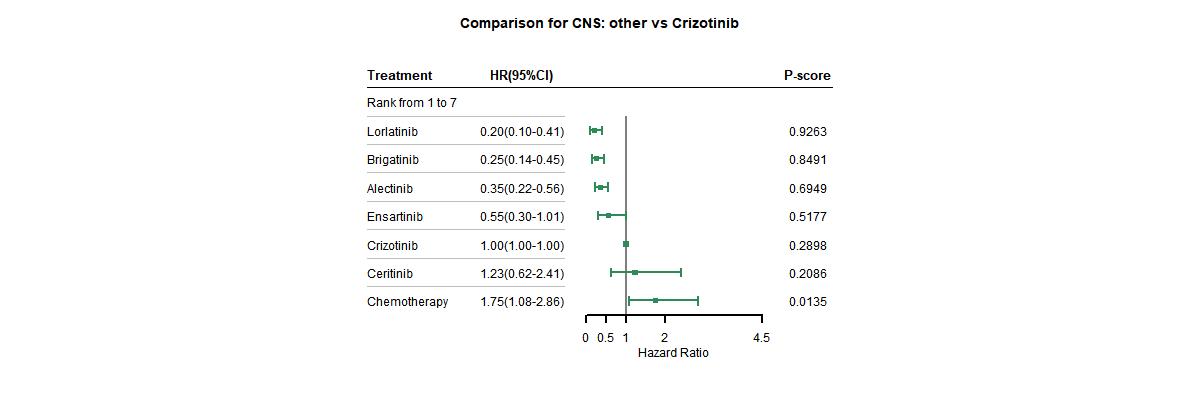 | 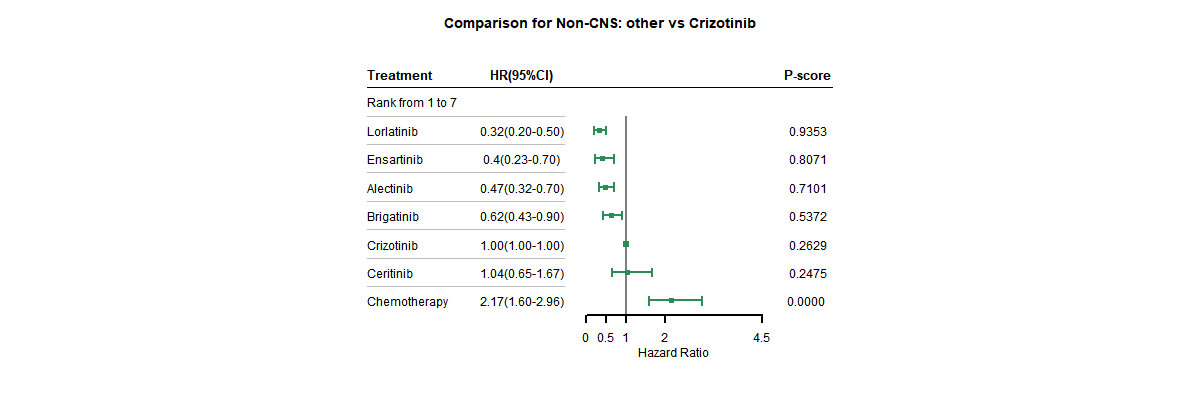 |
| C | D |
| 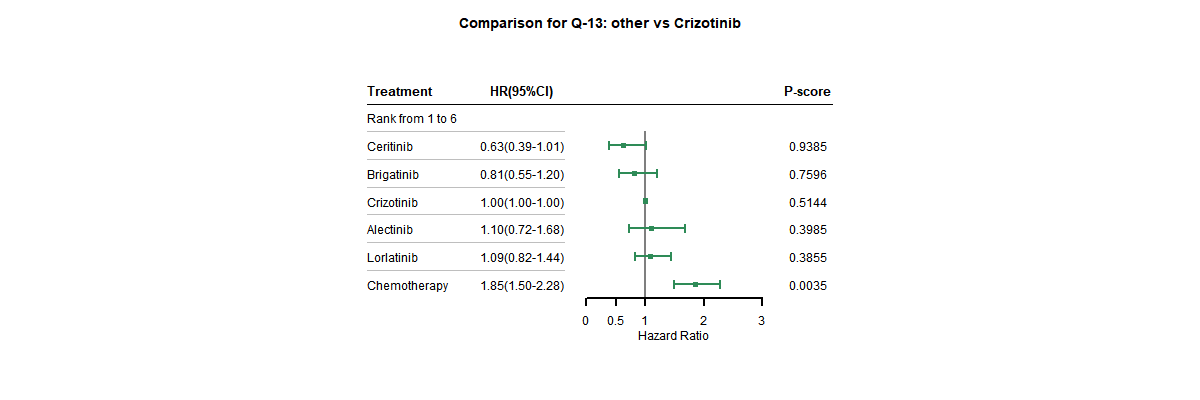 | 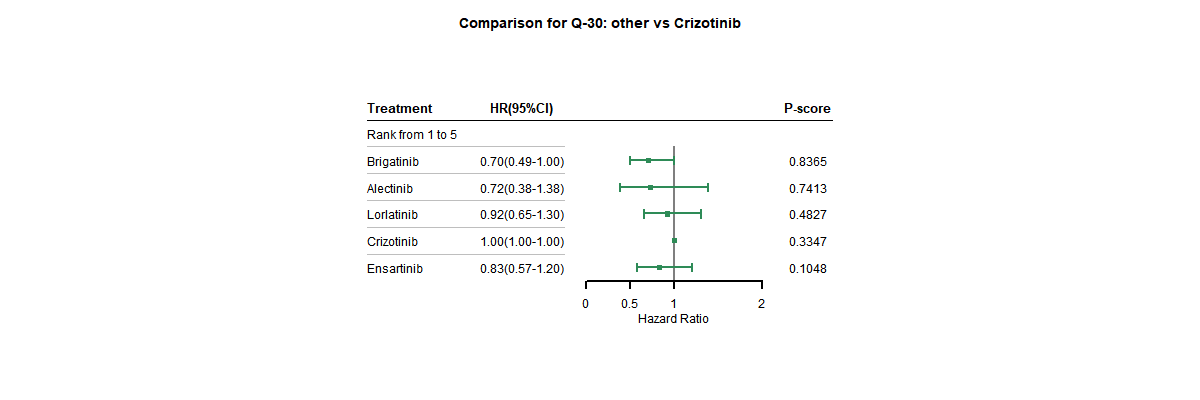 |

# eFigure 11 Forest plots for other subgroups based on Cox-PH model


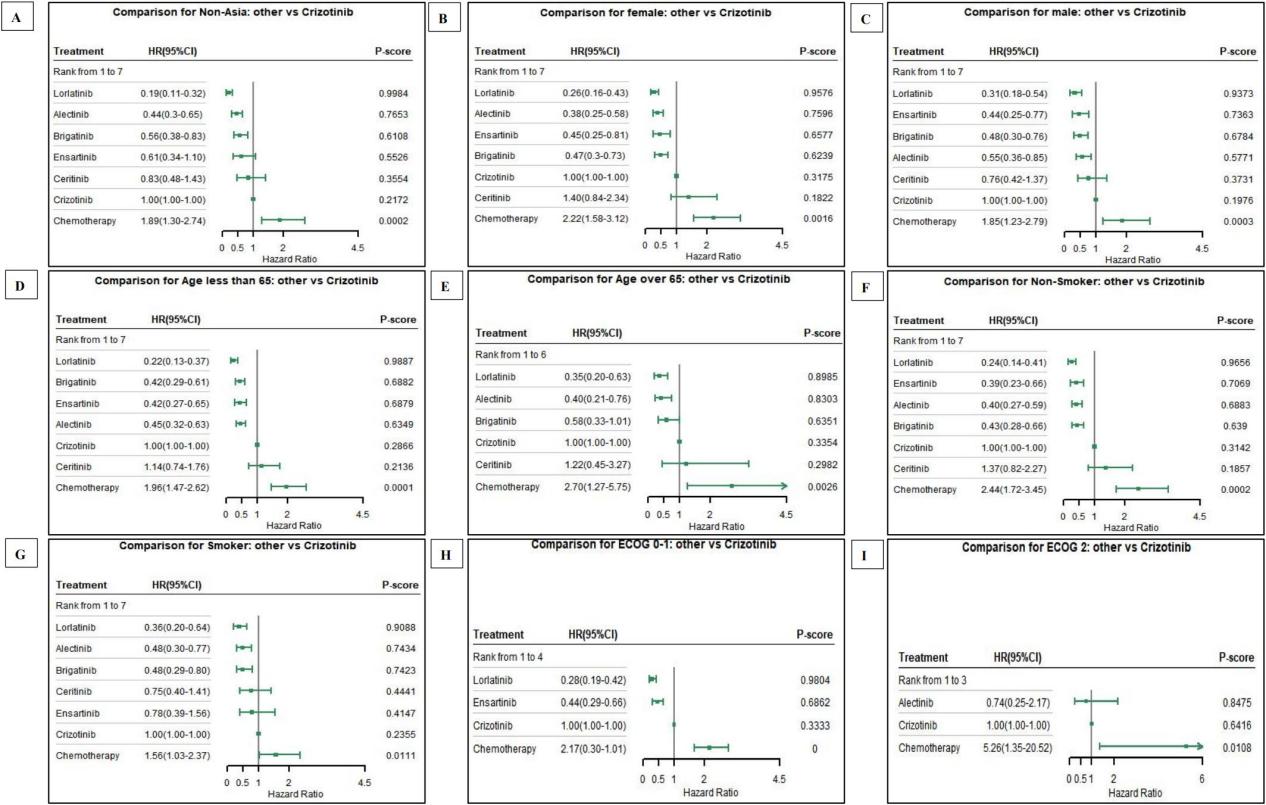


# eFigure 12 Summary Results of ORR

| 1A | 1B |
| --- | --- |
| 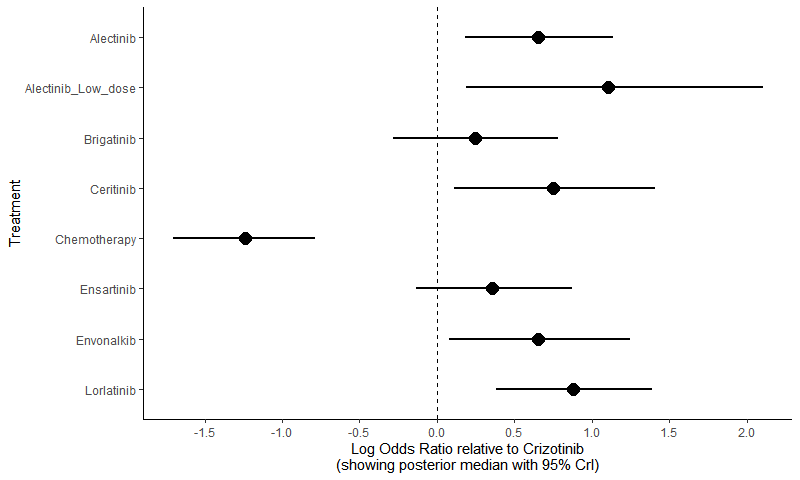 | 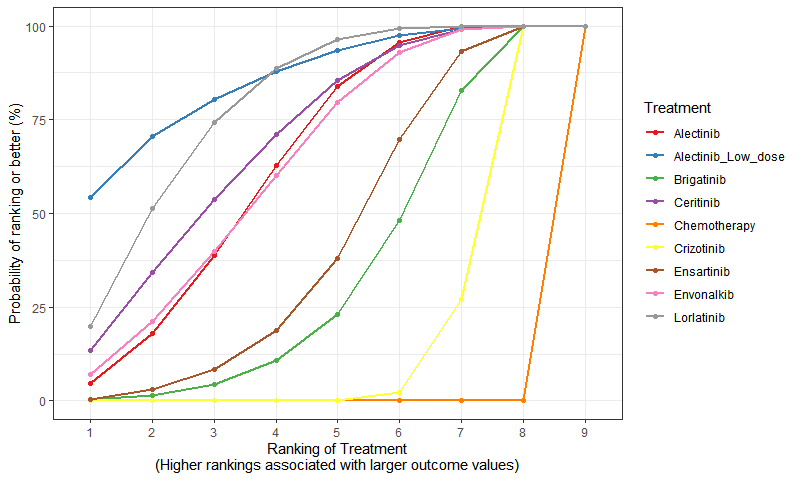 |
| 2A | 2B |
| 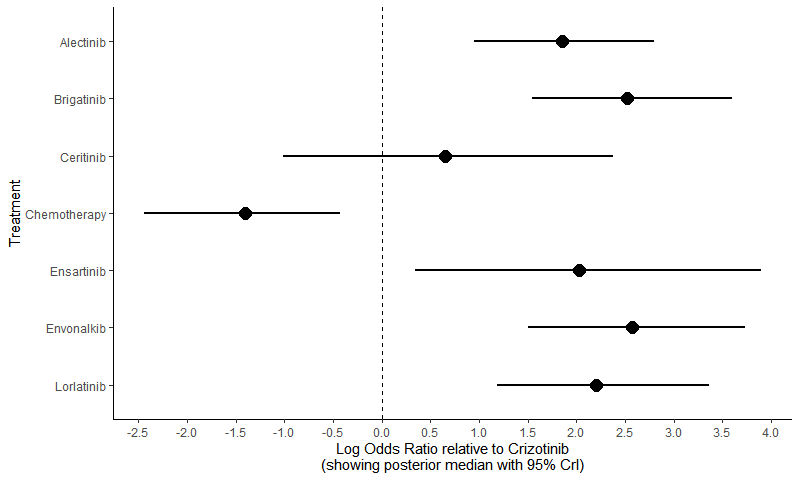 | 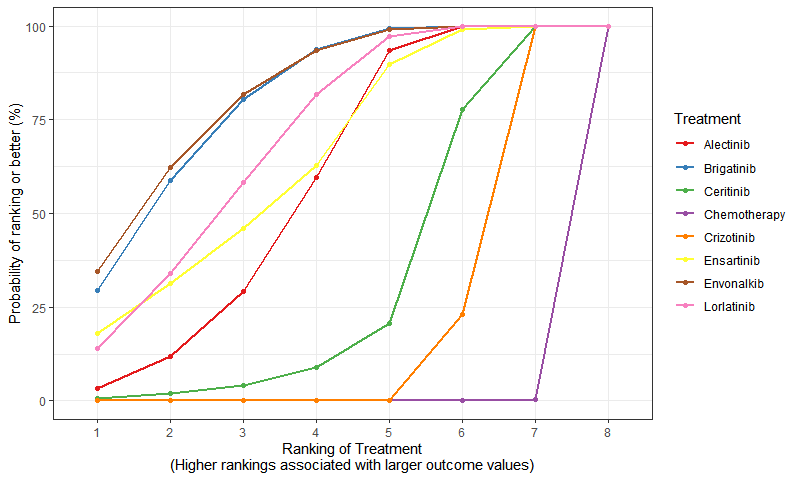 |
| 3A | 3B |
| 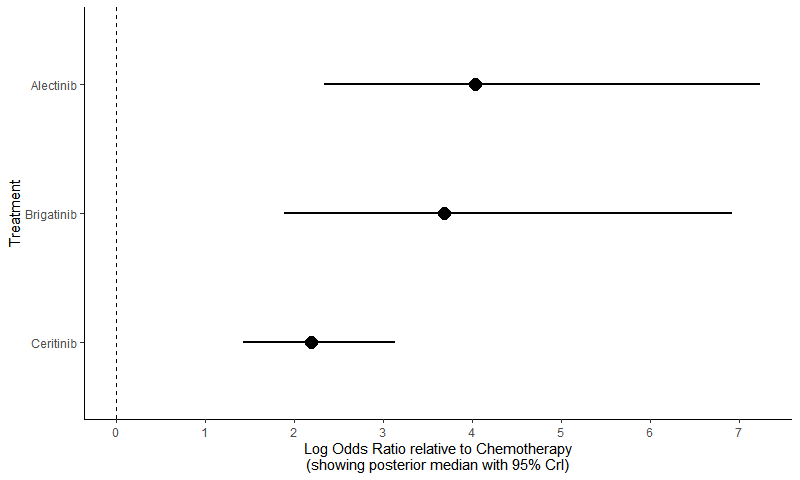 | 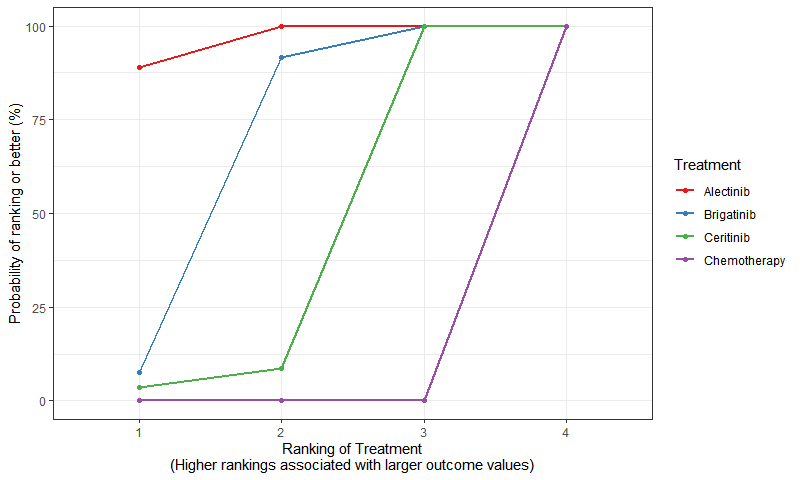 |
| 4A | 4B |
| 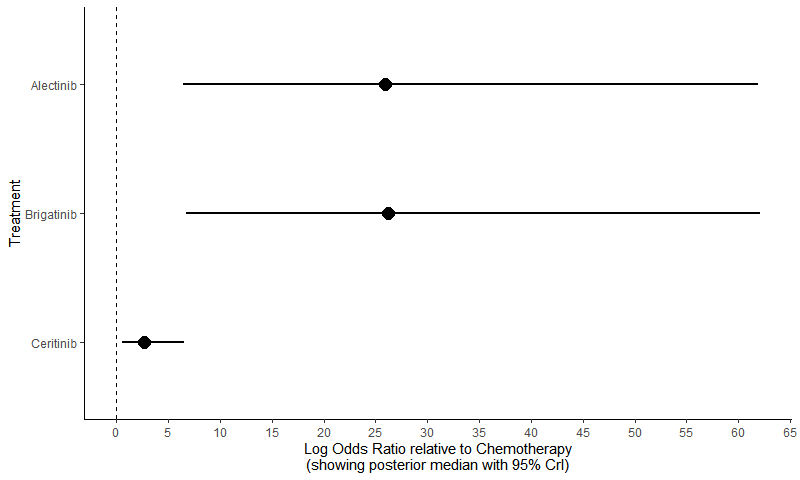 | 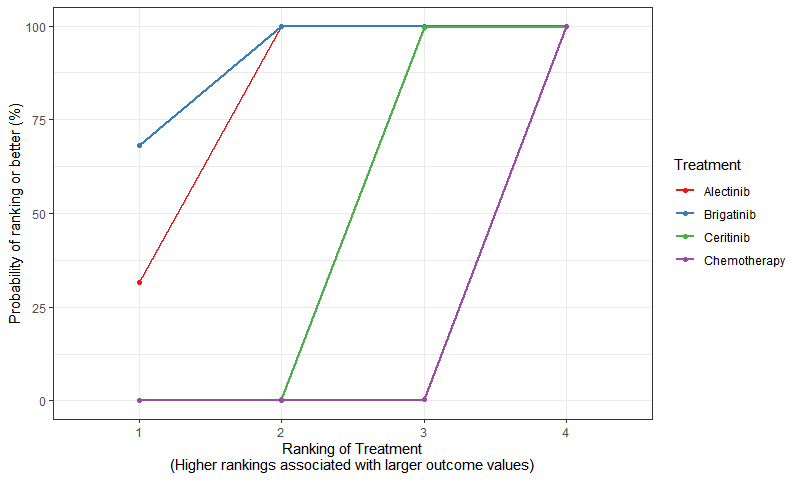 |

1A. Forest Plots for First-line Systemic ORR; 1B. Rank Plots for First-line Systemic ORR; 2A. Forest plots for First-line intracranial-ORR; 2B. Rank Plots for First-line intracranial-ORR; 3A. Forest Plots for Second-line Systemic ORR; 3B. Rank Plots for Second-line Systemic ORR; 4A. Forest Plots for Second-line intracranial-ORR; 4B. Rank Plots for Second-line intracranial-ORR

# eFigure 13 League table of ORR

(Reported by logarithm OR; A. First-line Systemic ORR; B. First-line intracranial-ORR; C. Second-line Systemic ORR; D. Second-line intracranial-ORR;)

| A | 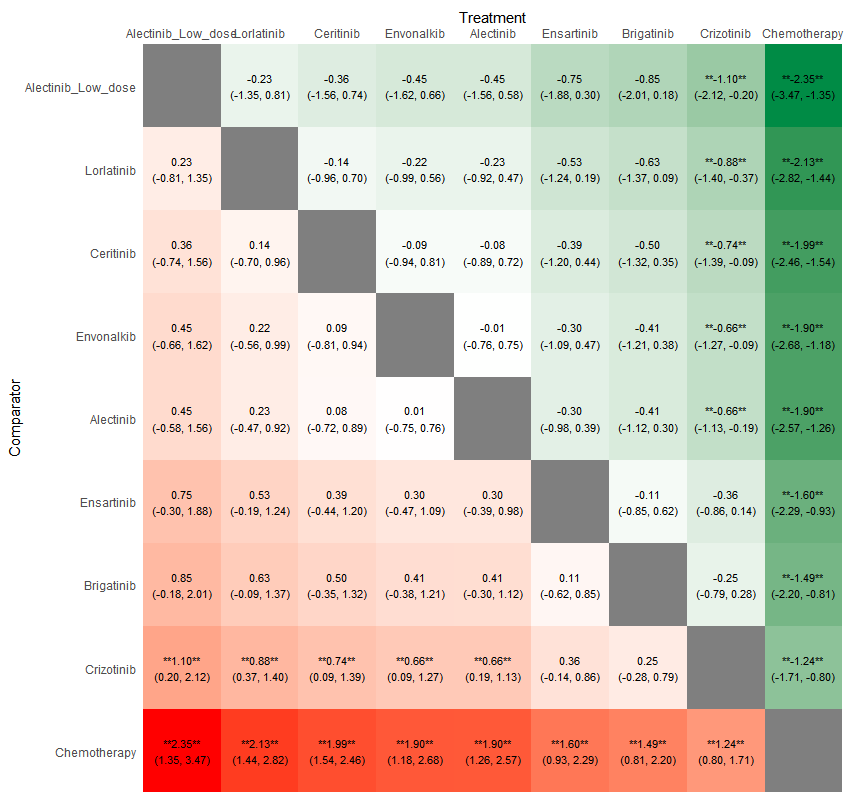 |
| --- | --- |
| B | 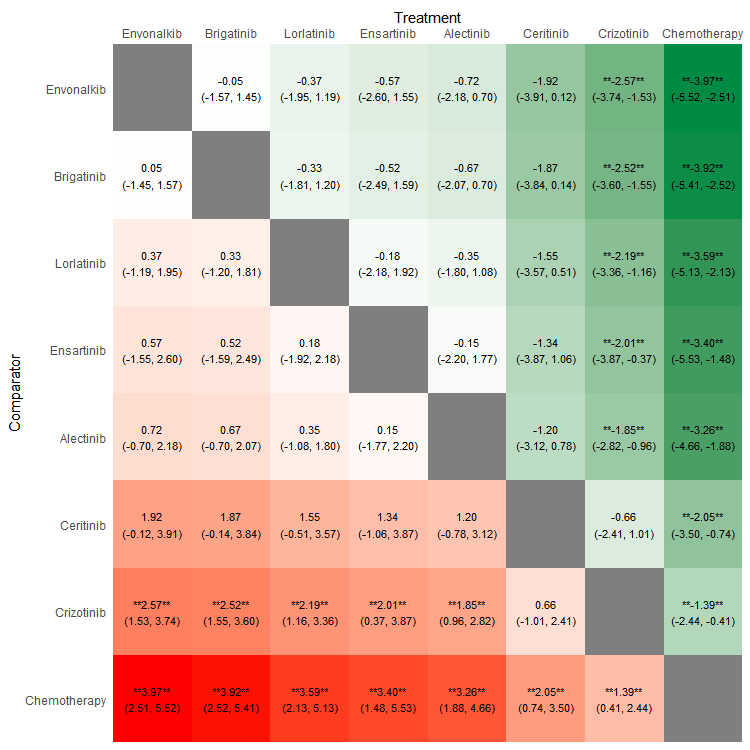 |
| C | 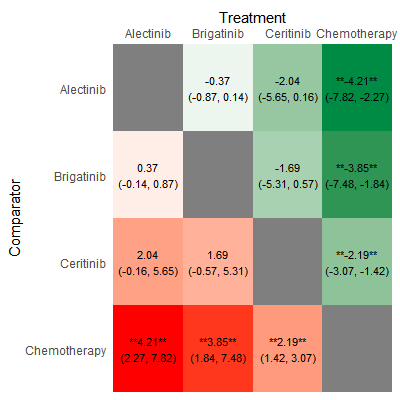 |
| D | 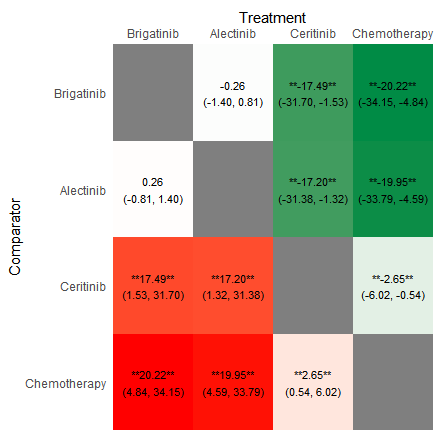 |

*significant difference

# eFigure 14 League table of AE

(Reported by logarithm OR; A. Any-grade AE for first-line treatment; B. Any-grade AE for second-line treatment; C. Grade 3-4 AE for first-line treatment; D. Grade 3-4 AE for second-line treatment; E. Grade 5 or fatal AE for first-line treatment;) *significant difference; AE, adverse event.

| 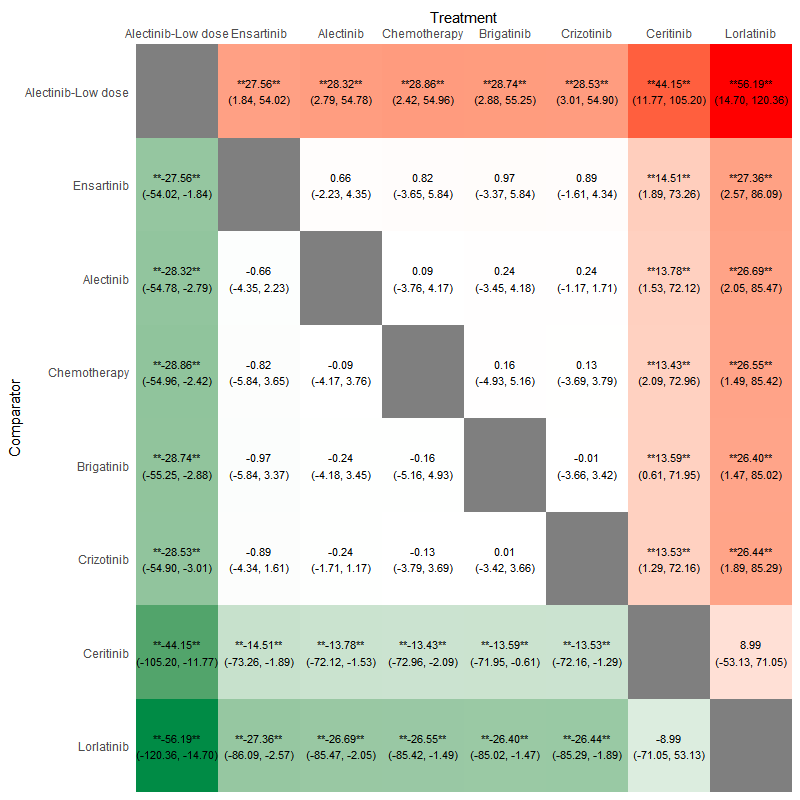 |
| --- |
| A |
| 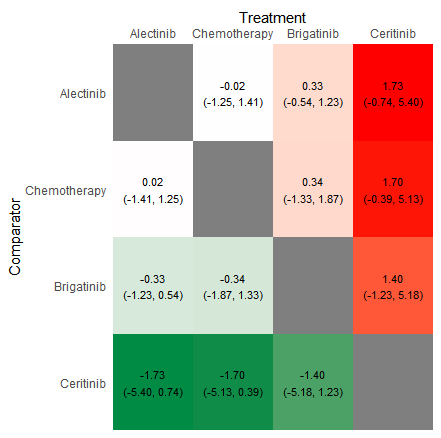 |
| B |
| 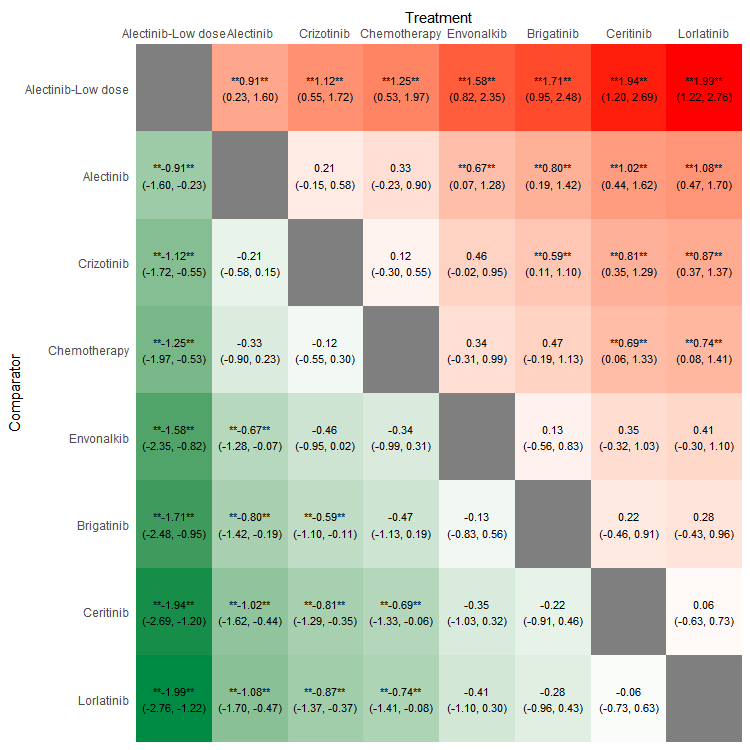 |
| C |
| 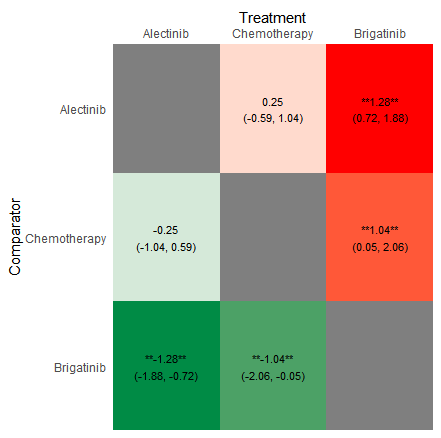 |
| D |
| 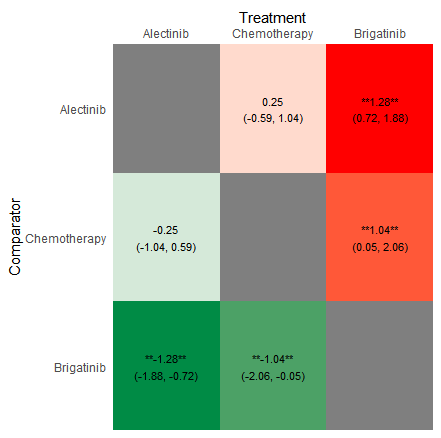 |
| E |

# eTable 1 Further baseline characteristics of included studies and patients

| Trial information | Intervention | Inclusion criteria | Age median (range) | Male percent (%) | ECOG performance status 0/1 percent (%) | Non Smoker percent (%) | Brain metastases at baseline percent (%) | Any-grade AE rate (%) | AE leading to discontinuation rate (%) | AE leading to drug-reduction rate (%) |
| --- | --- | --- | --- | --- | --- | --- | --- | --- | --- | --- |
| ALTA-1L | Brigatinib VS Crizotinib | Advanced ALK-positive NSCLC who had not previously received ALK inhibitors | 58 (27-86) VS 60(29-89) | 50VS 41 | 96 VS 96 | 61 VS 54 | 29 VS 23 | 100 VS 100 | 12 VS 9 | 29 vs 21 |
| PROFILE 1014 | Crizotinib VS Pemetrexed + Cisplatin/Carboplatin | Advanced ALK-positive nonsquamous NSCLC who had received no previous systemic treatment for advanced disease | 52 (22-76) VS 54 (19-78) | 40 VS 37 | 94 VS 95 | 62 VS 65 | 26 VS 27 | 99 VS 99 | 12 VS 14 | NA |
| CROWN | Lorlatinib VS Crizotinib | Advanced ALK-positive NSCLC who had received no previous systemic treatment | 61(51-69)VS 56(45-66) | 44 VS 38 | 98 VS 94 | 54 VS 64 | 26 VS 27 | 100 VS 99 | 7 VS 9 | 21 VS 15 |
| ALEX | Alectinib (600 mg twice daily)VS Crizotinib | Treatment-naive advanced ALK positive NSCLC | 56(25-88) VS 54(18-91) | 45 VS 42 | 93 VS 93 | 8 VS 3 | 42 VS 38 | 97 VS 97 | 13 VS 11 | 21 VS 16 |
| eXalt3 | Ensartinib VS Crizotinib | ALK-positive NSCLC | 54 (25-86) VS 53 (26-90) | 50 VS 52 | 95 VS 95 | 59 VS 64 | 33 VS 39 | 99 VS 100 | 9 VS 7 | 24 VS 20 |
| ASCEND-4 | Ceritinib VS Pemetrexed + Cisplatin/Carboplatin | untreated patients with stage IIIB/IV ALK-rearranged nonsquamous NSCLC | 55 (22-81) VS 54 (22-80) | 46 VS 39 | 94 VS 93 | 57 VS 65 | 31 VS 33 | 100 VS 97 | 5 VS 11 | NA |
| PROFILE 1029 | Crizotinib VS Pemetrexed + Cisplatin/Carboplatin | Previously untreated ALK-positive advanced NSCLC | 48 (24-67) VS 50 (23-69) | 48 VS 42 | 96 VS 96 | 75 VS 70 | 20 VS 31 | NA | 18 VS 4 | NA |
| J-ALEX | Low-dose alectinib (300 mg twice daily)VS Crizotinib | ALK inhibitor-naïve and chemotherapy-naïve, or had received one prior chemotherapy regimen patients with advanced ALK-positive NSCLC | 61(27-85) VS 60 (25-84) | 40 VS 39 | 98 VS 98 | 54 VS 59 | 14 VS 28 | 97 VS 100 | 9 VS 20 | NA |
| ALESIA | Alectinib (600 mg twice daily)VS Crizotinib | ALK inhibitor-naïve and chemotherapy-naïve NSCLC | 51 (43-59) VS 49 (41-59) | 51 VS 55 | 97 VS 98 | 67 VS 73 | 35 VS 37 | 100 VS 100 | 7 VS 10 | 24 VS 23 |
| TQ-B3139 | Envonalkib VS Crizotinib | ALK inhibitor-naïve and chemotherapy-naïve NSCLC | NA | NA | NA | NA | NA | NA | 5 VS 3 | NA |
| ALUR | Alectinib (600 mg twice daily) VS Pemetrexeor Docetaxel | Advanced/metastatic ALK-positive NSCLC patients previously treated with platinum-based doublet chemotherapy and crizotinib | 56 (21-82) VS 59 (37-80) | 57 VS 49 | 91 VS 85 | 49 VS 46 | 65 VS 74 | 90 VS 89 | 6 VS 9 | 4 VS 12 |
| ASCEND-5 | Ceritinib VS Pemetrexed or Docetaxel | ALK-rearranged advanced NSCLC who had received previous chemotherapy and crizotinib and had subsequent disease progression | 54(44-63)VS 54 (47-64) | 41VS 47 | 92 VS 96 | 62 VS 53 | 57 VS 59 | 99 VS 97 | 5 VS 7 | NA |
| ALTA-3 | Brigatinib VS Alectinib (600 mg twice daily) | Advanced/metastatic ALK-positive NSCLC patients previously treated with platinum-based doublet chemotherapy and crizotinib | 54 (22-83) VS 53 (28-82) | 46VS 45 | 100 VS 100 | 66 VS 69 | 64 VS 61 | NA | 5 VS 2 | 21 VS 11 |
| Profile 1007 | Crizotinib VS Pemetrexed or Docetaxel | Advanced/metastatic ALK-positive NSCLC patients previously treated with platinum-based doublet chemotherapy and crizotinib | 51 (22–81) VS 49 (24–85) | 43 VS 45 | 85 VS 92 | 62 VS 64 | 35 VS 34 | NA | 6 VS 10 | NA |

# eTable 2 Grade 3+ adverse events (incidence over 5%) for treatments

| Trial | Grade 3+ AE | Rate | Trial | Grade 3+ AE | Rate | Trial | Grade 3+ AE | Rate |
| --- | --- | --- | --- | --- | --- | --- | --- | --- |
| **Brigatinib** | | | **Chemotherapy** | | | **Crizotinib** | | |
| ALTA-1L | Blood creatine phosphokinase increased | 26% | PROFILE 1014 | Neutrophil count decreased | 15% | ALTA-1L | Lipase increased | 8% |
|  | Lipase increased | 15% |  | Anemia | 10% |  | Aspartate aminotransferase increased | 7% |
|  | Hypertension | 14% |  | Leukopenia | 5% |  | Gamma-glutamyltransferase increased | 7% |
|  | Amylase increased | 6% |  | Thrombocytopenia | 7% |  | Neutrophil count decreased | 7% |
|  | Pneumonia | 5% |  | Hypertriglyceridemia | 7% | PROFILE 1014 | Neutrophil count decreased | 15% |
|  | Alanine aminotransferase increased | 5% |  | Increased weight | 7% |  | Elevated transaminases | 14% |
| ALTA-3(second-line) | Blood creatine phosphokinase increased | 26% | ASCEND-4 | Neutrophil count decreased | 11% | CROWN | Alanine aminotransferase increased | 5% |
| **Ceritinib** | | |  | Anemia | 7% | ALEX | Alanine aminotransferase increased | 16% |
| ASCEND-4 | Blood alkaline phosphatase increased | 7% |  | Nausea | 5% |  | Aspartate aminotransferase increased | 11% |
|  | Alanine aminotransferase increased | 31% |  | Vomiting | 6% |  | Gamma-glutamyltransferase increased | 11% |
|  | Aspartate aminotransferase increased | 17% |  | Dyspnoea | 6% |  | Neutrophil count decreased | 5% |
|  | Gamma-glutamyltransferase increased | 17% | PROFILE 1029 | Neutrophil count decreased | 25% | eXalt3 | Alanine aminotransferase increased | 8% |
|  | Diarrhoea | 5% |  | Anemia | 13% | J-ALEX | Alanine aminotransferase increased | 13% |
|  | Vomiting | 5% |  | Leukopenia | 10% |  | Aspartate aminotransferase increased | 5% |
| ASCEND-5 | Blood alkaline phosphatase increased | 6% |  | Thrombocytopenia | 10% |  | Neutrophil count decreased | 14% |
|  | Alanine aminotransferase increased | 21% | ASCEND-5 (second-line) | Neutrophil count decreased | 15% |  | Electrocardiogram QT prolonged | 7% |
|  | Aspartate aminotransferase increased | 14% |  | Diarrhoea | 6% |  | Hepatic function abnormal | 6% |
|  | Gamma-glutamyltransferase increased | 21% |  | Asthenia | 6% | ALESIA | Alanine aminotransferase increased | 7% |
|  | Nausea | 8% |  | Dyspnoea | 6% |  | Neutrophil count decreased | 11% |
|  | Asthenia | 5% | ALUR (second-line) | Neutrophil count decreased | 12% |  | Decreased appetite | 5% |
|  | Fatigue | 5% |  | Anemia | 6% |  | Hyponatraemia | 5% |
|  | Vomiting | 8% |  | Fatigue | 9% | PROFILE 1029 | Elevated transaminases | 12 |
|  | Dyspnoea | 5% | Alectinib | | |  | Neutrophil count decreased | 16 |
| Alectinib Low-dose | | | ALEX | Alanine aminotransferase increased | 5% | Lorlatinib | | |
| J-ALEX | Blood creatine phosphokinase increased | 5% |  | Aspartate aminotransferase increased | 5% | CROWN | Hypertension | 10% |
|  | Interstitial lung disease | 5% |  | Gamma-glutamyltransferase increased | 5% |  | Hypercholesterolemia | 16% |
| Ensartinib | | |  | Anemia | 6% |  | Hypertriglyceridemia | 20% |
| eXalt3 | Rash | 11.20% | ALESIA | Blood creatine phosphokinase increased | 5% |  | Increased weight | 17% |
|  |  |  | ALUR (second-line) | Lipase increased | 7% |  |  |  |
|  |  |  |  | Hypertension | 6% |  |  |  |

# eTable.3 Convergence and heterogeneity assessment

| Network | Cochran’s Q | P-value | Network | I^2^ |
| --- | --- | --- | --- | --- |
| OS for first-line treatments on gloabl patients | 0 | NA | Any-grade adverse events for first-line treatments | 0 |
| PFS for first-line treatments on gloabl patients | 0 | NA | Any-grade adverse events for second-line treatments | 0 |
| PFS for first-line treatments on Asian patients | 1.045 | 0.79 | Grade 3-4 adverse events for first-line treatments | 0 |
| PFS for second-line treatments on gloabl patients previously given crizotinib | 0 | NA | Grade 3-4 adverse events for second-line treatments | 0 |
| Intracranial PFS for first-line treatments on global patients with baseline brain metastasis | 0 | NA | Grade 5 adverse events for first-line treatments | 0 |
| Intracranial PFS for first-line treatments on global patients without baseline brain metastasis | 0 | NA | First-line systemic ORR (Profile 1029 was excluded finally) | 79% (included Profile 1029); 36% (excluded Profile 1029) |
| PFS for first-line treatments on gloabl patients with baseline brain metastasis | 0 | NA | First-line systemic intracranial-ORR | 40% |
| PFS for first-line treatments on gloabl patients without baseline brain metastasis | 0 | NA | Second-line systemic ORR | 0 |
| PFS for first-line treatments on gloabl male patients | 0 | NA | Second-line systemic intracranial-ORR | 0 |
| PFS for first-line treatments on gloabl female patients | 0 | NA |  |  |
| PFS for first-line treatments on gloabl smoking patients | 0 | NA |  |  |
| PFS for first-line treatments on gloabl non-smoking patients | 0 | NA |  |  |
| PFS for first-line treatments on gloabl patients (age over 65) | 0 | NA |  |  |
| PFS for first-line treatments on gloabl patients (age less than 65) | 0 | NA |  |  |
| PFS for first-line treatments on non-Asian patients | 0 | NA |  |  |
| PFS for first-line treatments on gloabl patients (ECOG 0/1) | 0 | NA |  |  |
| PFS for first-line treatments on gloabl patients (ECOG 2) | 0 | NA |  |  |
| QLQ-LC13 for first-line treatments on gloabl patients | 0 | NA |  |  |
| QLQ-C30 for first-line treatments on gloabl patients | 0 | NA |  |  |

# eTable 4 League table for Cox-PH model

| For progression-free survival of first-line on Asian patients | | | | | | | | |
| --- | --- | --- | --- | --- | --- | --- | --- | --- |
| Alectinib |  |  |  |  |  |  |  |  |
| 1.08 (0.50 ~ 2.34) | Alectinib-Low dose |  |  |  |  |  |  |  |
| 1.05 (0.57 ~ 1.94) | 0.97 (0.40 ~ 2.38) | Brigatinib |  |  |  |  |  |  |
| 0.23 (0.13 ~ 0.43) | 0.22 (0.09 ~ 0.53) | 0.22 (0.10 ~ 0.48) | Ceritinib |  |  |  |  |  |
| 0.15 (0.10 ~ 0.23) | 0.14 (0.07 ~ 0.30) | 0.15 (0.08 ~ 0.27) | 0.66 (0.41 ~ 1.06) | Chemotherapy |  |  |  |  |
| 0.37 (0.27 ~ 0.49) | 0.34 (0.17 ~ 0.69) | 0.35 (0.20 ~ 0.60) | 1.57 (0.92 ~ 2.69) | 2.39 (1.85 ~ 3.08) | Crizotinib |  |  |  |
| 0.99 (0.58 ~ 1.72) | 0.92 (0.39 ~ 2.15) | 0.95 (0.46 ~ 1.93) | 4.25 (2.10 ~ 8.63) | 6.45 (3.80 ~10.93) | 2.70 (1.70 ~ 4.29) | Ensartinib |  |  |
| 0.84 (0.58 ~ 1.22) | 0.78 (0.37 ~ 1.65) | 0.80 (0.45 ~ 1.45) | 3.61 (2.02 ~ 6.47) | 5.47 (3.88 ~ 7.72) | 2.29 (1.82 ~ 2.89) | 0.85 (0.51 ~ 1.42) | Envonalkib |  |
| 0.92 (0.49 ~ 1.73) | 0.85 (0.34 ~ 2.11) | 0.87 (0.40 ~ 1.91) | 3.94 (1.81 ~ 8.56) | 5.96 (3.21 ~11.06) | 2.50 (1.42 ~ 4.39) | 0.93 (0.45 ~ 1.92) | 1.09 (0.59 ~ 2.01) | Lorlatinib |
| For overall survival of first-line on global patients | | | | | | |  |  |
| Alectinib |  |  |  |  |  |  |  |  |
| 0.83 (0.47 ~1.45) | Brigatinib |  |  |  |  |  |  |  |
| 0.70 (0.37 ~1.31) | 0.84 (0.44 ~1.62) | Ceritinib |  |  |  |  |  |  |
| 0.51 (0.31 ~0.84) | 0.62 (0.36 ~1.05) | 0.73 (0.50 ~1.07) | Chemotherapy |  |  |  |  |  |
| 0.67 (0.46 ~0.98) | 0.81 (0.53 ~1.23) | 0.96 (0.58 ~1.59) | 1.32 (0.95 ~1.82) | Crizotinib |  |  |  |  |
| 0.74 (0.39 ~1.40) | 0.89 (0.46 ~1.74) | 1.06 (0.51 ~2.19) | 1.45 (0.78 ~2.68) | 1.10 (0.65 ~1.86) | Ensartinib |  |  |  |
| 0.93 (0.47 ~1.82) | 1.13 (0.56 ~2.26) | 1.33 (0.63 ~2.83) | 1.83 (0.96 ~3.49) | 1.39 (0.80 ~2.43) | 1.26 (0.59 ~2.72) | Lorlatinib |  |  |
| For progression-free survival of first-line on global patients | | | | | | |  |  |
| Alectinib |  |  |  |  |  |  |  |  |
| 0.90 (0.58 ~ 1.38) | Brigatinib |  |  |  |  |  |  |  |
| 0.35 (0.22 ~ 0.57) | 0.39 (0.24 ~ 0.65) | Ceritinib |  |  |  |  |  |  |
| 0.19 (0.13 ~ 0.29) | 0.22 (0.14 ~ 0.33) | 0.55 (0.42 ~ 0.73) | Chemotherapy |  |  |  |  |  |
| 0.43 (0.32 ~ 0.58) | 0.48 (0.35 ~ 0.66) | 1.22 (0.83 ~ 1.80) | 2.22 (1.70 ~ 2.91) | Crizotinib |  |  |  |  |
| 0.86 (0.55 ~ 1.35) | 0.96 (0.60 ~ 1.53) | 2.44 (1.46 ~ 4.09) | 4.44 (2.88 ~ 6.86) | 2.00 (1.42 ~ 2.81) | Ensartinib |  |  |  |
| 1.59 (0.99 ~ 2.57) | 1.78 (1.09 ~ 2.90) | 4.53 (2.65 ~ 7.74) | 8.23 (5.19 ~13.04) | 3.70 (2.55 ~ 5.38) | 1.85 (1.12 ~ 3.07) | Lorlatinib |  |  |
| For progression-free survival of second-line on global patients | | | | |  |  |  |  |
| Alectinib |  |  |  |  |  |  |  |  |
| 1.03 (0.70 ~1.51) | Brigatinib |  |  |  |  |  |  |  |
| **0.41 (0.23 ~0.74)** | 0.40 (0.20 ~0.80) | Ceritinib |  |  |  |  |  |  |
| **0.20 (0.12 ~0.33)** | 0.19 (0.10 ~0.37) | 0.49 (0.36 ~0.67) | Chemotherapy |  |  |  |  |  |
| For progression-free survival of first-line on non-Asia patients | | | | | | |  |  |
| Alectinib |  |  |  |  |  |  |  |  |
| 0.79 (0.45 ~ 1.37) | Brigatinib |  |  |  |  |  |  |  |
| 0.53 (0.27 ~ 1.04) | 0.67 (0.34 ~ 1.32) | Ceritinib |  |  |  |  |  |  |
| 0.23 (0.14 ~ 0.40) | 0.30 (0.17 ~ 0.51) | 0.44 (0.30 ~ 0.65) | Chemotherapy |  |  |  |  |  |
| 0.44 (0.30 ~ 0.65) | 0.56 (0.38 ~ 0.83) | 0.83 (0.48 ~ 1.43) | 1.89 (1.30 ~ 2.74) | Crizotinib |  |  |  |  |
| 0.72 (0.35 ~ 1.47) | 0.92 (0.45 ~ 1.87) | 1.36 (0.61 ~ 3.04) | 3.09 (1.54 ~ 6.23) | 1.64 (0.91 ~ 2.96) | Ensartinib |  |  |  |
| 2.32 (1.19 ~ 4.50) | 2.95 (1.52 ~ 5.73) | 4.37 (2.04 ~ 9.36) | 9.93 (5.18 ~19.05) | 5.26 (3.09 ~ 8.98) | 3.21 (1.45 ~ 7.12) | Lorlatinib |  |  |
| For intracranial progression-free survival of first-line on global patients with baseline brain metastasis | | | | |  |  |  |  |
| Alectinib |  |  |  |  |  |  |  |  |
| 0.58 (0.23 ~ 1.45) | Brigatinib |  |  |  |  |  |  |  |
| 0.08 (0.03 ~ 0.25) | 0.14 (0.05 ~ 0.40) | Chemotherapy |  |  |  |  |  |  |
| 0.18 (0.09 ~ 0.36) | 0.31 (0.17 ~ 0.56) | 2.22 (0.94 ~ 5.27) | Crizotinib |  |  |  |  |  |
| 1.80 (0.54 ~ 6.03) | 3.10 (0.98 ~ 9.85) | 22 (5.97 ~82.7) | 10.00 (3.72 ~26.91) | Lorlatinib |  |  |  |  |
| For intracranial progression-free survival of first-line on global patients without baseline brain metastasis | | | | | |  |  |  |
| Alectinib |  |  |  |  |  |  |  |  |
| 0.18 (0.06 ~ 0.52) | Brigatinib |  |  |  |  |  |  |  |
| 0.10 (0.04 ~ 0.23) | 0.54 (0.28 ~ 1.04) | Chemotherapy |  |  |  |  |  |  |
| 0.14 (0.06 ~ 0.33) | 0.78 (0.41 ~ 1.48) | 1.45 (1.24 ~ 1.69) | Crizotinib |  |  |  |  |  |
| 0.44 (0.15 ~ 1.31) | 2.44 (0.95 ~ 6.23) | 4.53 (2.24 ~ 9.14) | 3.12 (1.57 ~ 6.20) | Ensartinib |  |  |  |  |
| 7.00 (0.72 ~ 68.12) | 39.00 (4.30 ~354) | 72.46 (8.74 ~601) | 50.00 (6.06 ~412) | 16.00 (1.74 ~147) | Lorlatinib |  |  |  |
| For progression-free survival of first-line on global patients with baseline brain metastasis | | | | | | |  |  |
| Alectinib |  |  |  |  |  |  |  |  |
| 1.40 (0.66 ~ 2.98) | Brigatinib |  |  |  |  |  |  |  |
| 0.29 (0.13 ~ 0.65) | 0.20 (0.08 ~ 0.50) | Ceritinib |  |  |  |  |  |  |
| 0.20 (0.10 ~ 0.39) | 0.14 (0.07 ~ 0.31) | 0.70 (0.44 ~ 1.12) | Chemotherapy |  |  |  |  |  |
| 0.35 (0.22 ~ 0.56) | 0.25 (0.14 ~ 0.45) | 1.23 (0.62 ~ 2.41) | 1.75 (1.08 ~ 2.86) | Crizotinib |  |  |  |  |
| 0.64 (0.30 ~ 1.37) | 0.45 (0.19 ~ 1.06) | 2.23 (0.90 ~ 5.54) | 3.19 (1.46 ~ 6.95) | 1.82 (0.99 ~ 3.34) | Ensartinib |  |  |  |
| 1.75 (0.74 ~ 4.16) | 1.25 (0.49 ~ 3.20) | 6.14 (2.27 ~16.60) | 8.77 (3.65 ~21.10) | 5.00 (2.41 ~10.37) | 2.75 (1.06 ~ 7.10) | Lorlatinib |  |  |
| For progression-free survival of first-line on global patients without baseline brain metastasis | | | | | | |  |  |
| Alectinib |  |  |  |  |  |  |  |  |
| 0.76 (0.44 ~ 1.31) | Brigatinib |  |  |  |  |  |  |  |
| 0.45 (0.24 ~ 0.83) | 0.59 (0.33 ~ 1.08) | Ceritinib |  |  |  |  |  |  |
| 0.22 (0.13 ~ 0.36) | 0.29 (0.18 ~ 0.46) | 0.48 (0.34 ~ 0.68) | Chemotherapy |  |  |  |  |  |
| 0.47 (0.32 ~ 0.70) | 0.62 (0.43 ~ 0.90) | 1.04 (0.65 ~ 1.67) | 2.17 (1.60 ~ 2.96) | Crizotinib |  |  |  |  |
| 1.17 (0.59 ~ 2.33) | 1.55 (0.79 ~ 3.03) | 2.61 (1.26 ~ 5.40) | 5.43 (2.88 ~10.27) | 2.50 (1.43 ~ 4.36) | Ensartinib |  |  |  |
| 1.47 (0.81 ~ 2.68) | 1.94 (1.08 ~ 3.47) | 3.26 (1.70 ~ 6.24) | 6.79 (3.94 ~11.70) | 3.12 (2.00 ~ 4.89) | 1.25 (0.61 ~ 2.55) | Lorlatinib |  |  |
| For progression-free survival of first-line on global male patients | | | | | | |  |  |
| Alectinib |  |  |  |  |  |  |  |  |
| 1.15 (0.61 ~ 2.16) | Brigatinib |  |  |  |  |  |  |  |
| 0.72 (0.35 ~ 1.51) | 0.63 (0.30 ~ 1.34) | Ceritinib |  |  |  |  |  |  |
| 0.30 (0.16 ~ 0.54) | 0.26 (0.14 ~ 0.48) | 0.41 (0.27 ~ 0.63) | Chemotherapy |  |  |  |  |  |
| 0.55 (0.36 ~ 0.85) | 0.48 (0.30 ~ 0.76) | 0.76 (0.42 ~ 1.37) | 1.85 (1.23 ~ 2.79) | Crizotinib |  |  |  |  |
| 1.25 (0.61 ~ 2.55) | 1.09 (0.53 ~ 2.25) | 1.73 (0.76 ~ 3.90) | 4.21 (2.10 ~ 8.45) | 2.27 (1.30 ~ 3.99) | Ensartinib |  |  |  |
| 1.77 (0.88 ~ 3.58) | 1.55 (0.76 ~ 3.17) | 2.45 (1.09 ~ 5.49) | 5.97 (3.01 ~11.87) | 3.23 (1.86 ~ 5.59) | 1.42 (0.65 ~ 3.12) | Lorlatinib |  |  |
| For progression-free survival of first-line on global female patients | | | | | | |  |  |
| Alectinib |  |  |  |  |  |  |  |  |
| 0.81 (0.44 ~ 1.49) | Brigatinib |  |  |  |  |  |  |  |
| 0.27 (0.14 ~ 0.53) | 0.34 (0.17 ~ 0.66) | Ceritinib |  |  |  |  |  |  |
| 0.17 (0.10 ~ 0.29) | 0.21 (0.12 ~ 0.37) | 0.63 (0.43 ~ 0.93) | Chemotherapy |  |  |  |  |  |
| 0.38 (0.25 ~ 0.58) | 0.47 (0.30 ~ 0.73) | 1.40 (0.84 ~ 2.34) | 2.22 (1.58 ~ 3.12) | Crizotinib |  |  |  |  |
| 0.84 (0.41 ~ 1.74) | 1.04 (0.50 ~ 2.18) | 3.11 (1.43 ~ 6.79) | 4.94 (2.51 ~ 9.73) | 2.22 (1.23 ~ 4.00) | Ensartinib |  |  |  |
| 1.46 (0.76 ~ 2.82) | 1.81 (0.92 ~ 3.54) | 5.38 (2.62 ~11.07) | 8.55 (4.65 ~15.71) | 3.85 (2.32 ~ 6.38) | 1.73 (0.80 ~ 3.76) | Lorlatinib |  |  |
| For progression-free survival of first-line on global smoking patients | | | | | | |  |  |
| Alectinib |  |  |  |  |  |  |  |  |
| 1.00 (0.50 ~2.00) | Brigatinib |  |  |  |  |  |  |  |
| 0.64 (0.29 ~1.41) | 0.64 (0.28 ~1.44) | Ceritinib |  |  |  |  |  |  |
| 0.31 (0.16 ~0.58) | 0.31 (0.16 ~0.59) | 0.48 (0.30 ~0.77) | Chemotherapy |  |  |  |  |  |
| 0.48 (0.30 ~0.77) | 0.48 (0.29 ~0.80) | 0.75 (0.40 ~1.41) | 1.56 (1.03 ~2.37) | Crizotinib |  |  |  |  |
| 0.62 (0.27 ~1.43) | 0.62 (0.26 ~1.46) | 0.96 (0.38 ~2.46) | 2.00 (0.89 ~4.51) | 1.28 (0.64 ~2.57) | Ensartinib |  |  |  |
| 1.33 (0.63 ~2.80) | 1.33 (0.62 ~2.87) | 2.08 (0.89 ~4.89) | 4.34 (2.13 ~8.83) | 2.78 (1.57 ~4.93) | 2.17 (0.88 ~5.34) | Lorlatinib |  |  |
| For progression-free survival of first-line on global non-smoking patients | | | | | | |  |  |
| Alectinib |  |  |  |  |  |  |  |  |
| 0.93 (0.52 ~ 1.65) | Brigatinib |  |  |  |  |  |  |  |
| 0.29 (0.15 ~ 0.56) | 0.31 (0.16 ~ 0.61) | Ceritinib |  |  |  |  |  |  |
| 0.16 (0.10 ~ 0.28) | 0.18 (0.10 ~ 0.30) | 0.56 (0.39 ~ 0.81) | Chemotherapy |  |  |  |  |  |
| 0.40 (0.27 ~ 0.59) | 0.43 (0.28 ~ 0.66) | 1.37 (0.82 ~ 2.27) | 2.44 (1.72 ~ 3.45) | Crizotinib |  |  |  |  |
| 1.03 (0.54 ~ 1.96) | 1.10 (0.56 ~ 2.15) | 3.50 (1.69 ~ 7.25) | 6.25 (3.35 ~11.68) | 2.56 (1.53 ~ 4.31) | Ensartinib |  |  |  |
| 1.67 (0.87 ~ 3.21) | 1.79 (0.91 ~ 3.51) | 5.69 (2.74 ~11.82) | 10.16 (5.42 ~19.06) | 4.17 (2.47 ~ 7.04) | 1.62 (0.78 ~ 3.40) | Lorlatinib |  |  |
| For progression-free survival of first-line on global patients aging less than 65 | | | | | | |  |  |
| PFS- AGE< 65 |  |  |  |  |  |  |  |  |
| Alectinib |  |  |  |  |  |  |  |  |
| 1.07 (0.64 ~ 1.78) | Brigatinib |  |  |  |  |  |  |  |
| 0.40 (0.23 ~ 0.69) | 0.37 (0.21 ~ 0.66) | Ceritinib |  |  |  |  |  |  |
| 0.23 (0.15 ~ 0.36) | 0.21 (0.13 ~ 0.35) | 0.58 (0.42 ~ 0.80) | Chemotherapy |  |  |  |  |  |
| 0.45 (0.32 ~ 0.63) | 0.42 (0.29 ~ 0.61) | 1.14 (0.74 ~ 1.76) | 1.96 (1.47 ~ 2.62) | Crizotinib |  |  |  |  |
| 1.07 (0.62 ~ 1.85) | 1.00 (0.56 ~ 1.78) | 2.71 (1.47 ~ 4.99) | 4.67 (2.77 ~ 7.86) | 2.38 (1.55 ~ 3.67) | Ensartinib |  |  |  |
| 2.05 (1.10 ~ 3.81) | 1.91 (1.00 ~ 3.64) | 5.17 (2.62 ~10.20) | 8.91 (4.90 ~16.21) | 4.55 (2.69 ~ 7.67) | 1.91 (0.97 ~ 3.76) | Lorlatinib |  |  |
| For progression-free survival of first-line on global patients aging over 65 | | | | | |  |  |  |
| Alectinib |  |  |  |  |  |  |  |  |
| 0.69 (0.30 ~ 1.61) | Brigatinib |  |  |  |  |  |  |  |
| 0.33 (0.10 ~ 1.07) | 0.48 (0.15 ~ 1.49) | Ceritinib |  |  |  |  |  |  |
| 0.15 (0.06 ~ 0.40) | 0.21 (0.08 ~ 0.55) | 0.45 (0.24 ~ 0.85) | Chemotherapy |  |  |  |  |  |
| 0.40 (0.21 ~ 0.76) | 0.58 (0.33 ~ 1.01) | 1.22 (0.45 ~ 3.27) | 2.70 (1.27 ~ 5.75) | Crizotinib |  |  |  |  |
| 1.14 (0.48 ~ 2.71) | 1.66 (0.74 ~ 3.71) | 3.47 (1.10 ~10.94) | 7.72 (2.98 ~20.03) | 2.86 (1.60 ~ 5.11) | Lorlatinib |  |  |  |
| For progression-free survival of first-line on global patients with ECOG 0 or 1 | | | |  |  |  |  |  |
| Chemotherapy |  |  |  |  |  |  |  |  |
| 2.17 (1.68 ~ 2.81) | Crizotinib |  |  |  |  |  |  |  |
| 4.94 (3.06 ~ 7.98) | 2.27 (1.52 ~ 3.40) | Ensartinib |  |  |  |  |  |  |
| 7.76 (4.84 ~12.46) | 3.57 (2.40 ~ 5.31) | 1.57 (0.89 ~ 2.77) | Lorlatinib |  |  |  |  |  |
| For progression-free survival of first-line on global patients with ECOG 2 | | | |  |  |  |  |  |
| Alectinib |  |  |  |  |  |  |  |  |
| 0.14 (0.02 ~ 0.80) | Chemotherapy |  |  |  |  |  |  |  |
| 0.74 (0.25 ~ 2.17) | 5.26 (1.35 ~20.52) | Crizotinib | / |  |  |  |  |  |
| For QLQ-LC13 of first-line on global patients | | | | | |  |  |  |
| Alectinib |  |  |  |  |  |  |  |  |
| 1.36 (0.76 ~2.42) | Brigatinib |  |  |  |  |  |  |  |
| 1.75 (0.92 ~3.32) | 1.29 (0.69 ~2.40) | Ceritinib |  |  |  |  |  |  |
| 0.60 (0.37 ~0.96) | 0.44 (0.28 ~0.69) | 0.34 (0.22 ~0.52) | Chemotherapy |  |  |  |  |  |
| 1.10 (0.72 ~1.68) | 0.81 (0.55 ~1.20) | 0.63 (0.39 ~1.01) | 1.85 (1.50 ~2.28) | Crizotinib |  |  |  |  |
| 1.01 (0.61 ~1.68) | 0.74 (0.46 ~1.21) | 0.58 (0.33 ~1.00) | 1.69 (1.19 ~2.41) | 0.92 (0.69 ~1.22) | Lorlatinib |  |  |  |
| For QLQ-C30 of first-line on global patients | | | | |  |  |  |  |
| Alectinib |  |  |  |  |  |  |  |  |
| 1.03 (0.49 ~2.16) | Brigatinib |  |  |  |  |  |  |  |
| 0.72 (0.38 ~1.38) | 0.70 (0.49 ~1.00) | Crizotinib |  |  |  |  |  |  |
| 0.87 (0.41 ~1.83) | 0.84 (0.50 ~1.41) | 1.20 (0.83 ~1.75) | Ensartinib |  |  |  |  |  |
| 0.78 (0.38 ~1.63) | 0.76 (0.46 ~1.25) | 1.09 (0.77 ~1.53) | 0.90 (0.54 ~1.50) | Lorlatinib |  |  |  |  |

# eTable 5 EORTC QLQ-C30 Scores, Change From Baseline^*^

| study | Global quality of life | Fatigue | Nausea and vomiting | Pain | Dyspnea | Insomnia | Appetite loss | Constipation | Diarrhea |
| --- | --- | --- | --- | --- | --- | --- | --- | --- | --- |
| ALTA-1L,brigatinib VS crizotinib | 3.1 (-0.8 to 7.0) | -4.3(-8.3 to -0.4) | -2.3 (-4.3 to -0.2) | -0.5 (-4.5 to 3.4) | 0.2 (-3.9 to 4.4) | -1.7 (-6 to 2.6) | -5.5 (-9.3 to -1.8) | -10.6 (-14.7 to -6.5) | -1 (-5 to 3.1) |
| ASCEND-4,ceritinib VS chemotherapy | 4.52 (2.04-6.90) | -4.93(-7.68 to -2.03) | 3.46 (1.32 to 5.75) | -2.64 (-5.24 to -0.2) | -7.22 (-9.97 to -4.47) | -4.63 (-7.53 to -1.73) | -1.12 (-4.32 to 1.93) | -4.32 (-6.46 to -2.19) | 22.07 (19.47 to 24.81) |
| CROWN, lorlatinib VS crizotinib | 4.65(1.14,8.16) | -5.67(-9.42 to -1.92) | -7.86 (-9.86 to -5.86) | 1.16(-2.49 to 4.82) | 1.72 (-1.98 to 5.43) | -7.95 (-11.25 to -4.64) | -9.21 (-11.8 to -6.62) | -4.93 (-9.07 to -0.97) | -12.03 (-15.49 to -8.58) |
| Profile 1014,crizotinib VS pemetrexed | 13.72 | -8.38(-13.65 to -3.11) | 1.47 (-1.69 to 4.63) | -9.59 (-14.09 to -5.08) | -10.68(-16.31 to -5.06) | -6.16 (-10.87 to -1.45) | -4.13 (-8.83 to 0.57) | 7.55 (1.68 to 13.42) | 10.04 (4.72 to 15.35) |
| Profile 1014,crizotinib VS docetaxel |  | -22.26(-30.75 to -13.76) | -5.56 (-12.41 to 1.28) | -25.64 (-33.66 to -17.62) | -22.16(-31.39 to -12.92) | -20.01 (-28.42 to -11.6) | -11.45 (-20.02 to -2.89) | 10.05 (-0.6 to 20.69) | 15.24 (5.55 to 24.92) |

* Data were presented as the mean difference (95% confidence interval) between the mean changes from baseline for the specific indicator between two treatments.
