# Supplementary material for: Identifying optimal ALK inhibitors in first- and second-line treatment of patients with advanced ALK-positive non-small-cell lung cancer: a systematic review and network meta-analysis
Source: BMC Cancer. 2024 Feb 8;24:186. doi: 10.1186/s12885-024-11916-4 (PMC10851546; doi:10.1186/s12885-024-11916-4)
Supplement: Supplementary file 6 — Additional file 6. [file 12885_2024_11916_MOESM6_ESM.docx]

**Additional file 6**

[Table 1 Network of OS and PFS for first-line treatments on gloabl patients 2](#_Toc20308)

[Table 2 Network of PFS for first-line treatments on Asian patients and second-line treatments on gloabl patients previously given crizotinib 4](#_Toc13050)

[Table 3 Network of CNS PFS for first-line treatments on global patients with or without baseline brain metastasis 6](#_Toc7395)

[Table 4 Network of PFS for first-line treatments on global patients with or without baseline brain metastasis 8](#_Toc25611)

[Table 5 Network of patient report outcomes 10](#_Toc30110)

# Table 1 Network of OS and PFS for first-line treatments on gloabl patients

| Study | Arm 1 | Arm 2 | Network of OS for first-line treatments on gloabl patients | | | | Network of PFS for first-line treatments on gloabl patients | | | |
| --- | --- | --- | --- | --- | --- | --- | --- | --- | --- | --- |
|  |  |  | RMSD [95% CI] (mean ranking in the NMA)* | Life-years gain in 5 years (ranking in the NMA)^#^ | Life-years gain in 10 years (ranking in the NMA)^#^ | HR [95% CI] (P-score in the NMA)^†^ | RMSD [95% CI] (mean ranking in the NMA)^*^ | Progressin-free life-years gain in 5 years (ranking in the NMA)^#^ | Progressin-free life-years gain in 10 years (ranking in the NMA)^#^ | HR [95% CI] (P-score in the NMA)^†^ |
| ALEX^[1, 2]^ | Alectinib | Crizotinib | 1.13[-1.32-3.61] (2.856) / 0(4.641) | 0.43(1) / 0(6) | 1.17(1) / 0(6) | 0.67[0.46-0.98] (0.833/ 0.342) | 4.99[2.77-7.23] (2.891) / 0(5.484) | 1.22(2) / 0(5) | 2.93(2) / 0(5) | 0.43[0.32-0.58] (0.744/ 0.308) |
| CROWN^[3, 4]^ | Lorlatinib | Crizotinib | 1.11[-1.25-3.48] (2.867) / RE | 0.42(2) / RE | 0.82(2) / RE | 0.72[0.41-1.26] (0.732/ RE) | 8.67[6.44-10.92] (1.034) / RE | 1.98(1) / RE | 4.74(1) / RE | 0.27[0.19-0.39] (0.992/ RE) |
| ALTA-1L^[5]^ | Brigatinib | Crizotinib | 0.14[-2.34-2.62] (4.286) / RE | 0.25(3) / RE | 0.80(3) / RE | 0.81[0.53-1.23] (0.626/ RE) | 5.18[2.74-7.63] (2.752) / RE | 1.08(3) / RE | 1.97(4) / RE | 0.48[0.35-0.66] (0.648/ RE) |
| eXalt3^[6]^ | Ensartinib | Crizotinib | 0.15[-2.21-2.53] (4.279) / RE | 0.14(5) / RE | 0.62(4) / RE | 0.91[0.54-1.54] (0.482/ RE) | 4.34[1.94-6.76] (3.339) / RE | 1.04(4) / RE | 2.02(3) / RE | 0.50[0.36-0.70] (0.616/ RE) |
| PROFILE 1014^[7, 8]^ | Chemotherapy | Crizotinib | -1.21[-3.59-1.21] (6.186)/ RE | -0.32(7)/ RE | -0.78(7)/ RE | 1.32[0.95-1.82] (0.050/ RE) | -5.49[-7.28~-3.68] (7.000)/ RE | -0.90(7)/ RE | -1.07(7)/ RE | 2.22[1.70-2.91] (0.000/ RE) |
| ASCEND-4^[9]^ | Ceritinib | Chemotherapy | 1.20[-2.30-4.73] (2.886) / RE | 0.18(4)/ RE | 0.25(5)/ RE | 0.96[0.58-1.59] (0.833/ RE) | -0.06[-2.88-2.77] (5.500) / RE | -0.46(6)/ RE | -0.63(6)/ RE | 1.22[0.83-1.80] (0.192/ RE) |
| PROFILE 1029 | Crizotinib | Chemotherapy | Not included | Not included | Not included | Not included | Not included | Not included | Not included | Not included |
| J-ALEX | Low-dose alectinib | Crizotinib | Not included | Not included | Not included | Not included | Not included | Not included | Not included | Not included |
| ALESIA | Alectinib | Crizotinib | Not included | Not included | Not included | Not included | Not included | Not included | Not included | Not included |
| TQ-B3139 | Envonalkib | Crizotinib | Not included | Not included | Not included | Not included | Not included | Not included | Not included | Not included |
| ALUR | Alectinib | Chemotherapy | Not included | Not included | Not included | Not included | Not included | Not included | Not included | Not included |
| ASCEND-5 | Ceritinib | Chemotherapy | Not included | Not included | Not included | Not included | Not included | Not included | Not included | Not included |
| ALTA-3 | Brigatinib | Alectinib | Not included | Not included | Not included | Not included | Not included | Not included | Not included | Not included |
| Profile 1007 | Crizotinib | Chemotherapy | Not included | Not included | Not included | Not included | Not included | Not included | Not included | Not included |
| Sarah-2022 | Lorlatinib | Chemotherapy | Not included | Not included | Not included | Not included | Not included | Not included | Not included | Not included |

*, compared to crizotinib, month; #, compared to crizotinib, predicted by FP model, year；†, compared to crizotinib using Cox-PH model;

# Table 2 Network of PFS for first-line treatments on Asian patients and second-line treatments on gloabl patients previously given crizotinib

| Study | Arm 1 | Arm 2 | Network of PFS for first-line treatments on Asian patients | | | | Network of PFS for second-line treatments on gloabl patients previously given crizotinib | | | |
| --- | --- | --- | --- | --- | --- | --- | --- | --- | --- | --- |
|  |  |  | RMSD [95% CI] (mean ranking in the NMA)^*^ | Progressin-free life-years gain in 5 years (ranking in the NMA)^#^ | Progressin-free life-years gain in 10 years (ranking in the NMA)^#^ | HR [95% CI] (P-score in the NMA)^†^ | RMSD [95% CI] (mean ranking in the NMA)^*^ | Progressin-free life-years gain in 5 years (ranking in the NMA)^#^ | Progressin-free life-years gain in 10 years (ranking in the NMA)^#^ | HR [95% CI] (P-score in the NMA)^†^ |
| ALEX$^[2]^ | Alectinib | Crizotinib | Not included | Not included | Not included | 0.37[0.27-0.49] (0.723/ 0.244) | Not included | Not included | Not included | Not included |
| CROWN^&[10]^ | Lorlatinib | Crizotinib | 4.00[1.23-6.77] (4.347) / 0(7.169) | 1.32(4) / 0(7) | 3.18(3) / 0(7) | 0.40[0.23-0.70] (0.644/ RE) | Not included | Not included | Not included | Not included |
| ALTA-1L^&[11]^ | Brigatinib | Crizotinib | 4.53[1.85-7.19] (3.716) / RE | 1.26(5) / RE | 2.80(5) / RE | 0.35[0.20-0.60] (0.750/ RE) | Not included | Not included | Not included | Not included |
| eXalt3^&[6]^ | Ensartinib | Crizotinib | 5.21[3.08-7.38] (2.842) / RE | 1.46(2) / RE | 3.16(4) / RE | 0.37[0.23-0.59] (0.708/ RE) | Not included | Not included | Not included | Not included |
| PROFILE 1014^&[12]^ | Chemotherapy | Crizotinib | -4.19[-6.26~ -2.14] (8.972)/ RE | -1.47(9)/ RE | -2.89(9)/ RE | 2.39[1.85-3.08] (0.005/ RE) | Not included | Not included | Not included | Not included |
| ASCEND-4^&[13]^ | Ceritinib | Chemotherapy | -1.62[-4.98-1.75] (7.843) / RE | -0.53(8) / RE | -0.91(8) / RE | 1.57[0.92-2.69] (0.126/ RE) | Not included | Not included | Not included | Not included |
| PROFILE 1029^[14]^ | Crizotinib | Chemotherapy | RE/ RE | RE/ RE | RE/ RE | RE/ RE | Not included | Not included | Not included | Not included |
| J-ALEX^[15]^ | Low-dose alectinib | Crizotinib | 5.21[3.38-7.05] (2.810) / RE | 1.43(3) / RE | 3.19(2)/ RE | 0.34[0.17-0.69] (0.757/RE) | Not included | Not included | Not included | Not included |
| ALESIA^[16, 17]^ | Alectinib | Crizotinib | 5.73[3.70-7.74] (2.177) / RE | 1.58(1) / RE | 3.32(1) / RE | RE/ RE | Not included | Not included | Not included | Not included |
| TQ-B3139^[18]^ | Envonalkib | Crizotinib | 3.48[1.81-5.19] (5.124) / RE | 1.00(6) / RE | 2.09(6) / RE | 0.44[0.35-0.55] (0.542/RE) | Not included | Not included | Not included | Not included |
| ALUR^[19]^ | Alectinib | Chemotherapy | Not included | Not included | Not included | Not included | 7.96[6.02-9.91] (1.425) / 0(4) | 1.07(1)/ 0(4) | 1.12(1)/ 0(4) | 0.20[0.12-0.33] (0.764/0) |
| ASCEND-5^[20]^ | Ceritinib | Chemotherapy | Not included | Not included | Not included | Not included | 3.14[1.80-4.47] (2.999) / RE | 0.49(3)/ RE | 0.55(3)/ RE | 0.49[0.36-0.67] (0.252/RE) |
| ALTA-3^[16]^ | Brigatinib | Alectinib | Not included | Not included | Not included | Not included | 7.81[5.30-10.32] (1.576) / RE | 0.84(2)/ 0(4) | 0.84(2)/ 0(4) | 0.19[0.10-0.37] (0.797/RE) |
| Profile 1007^[21]^ | Crizotinib | Chemotherapy | Not included | Not included | Not included | Not included | Not included | Not included | Not included | Not included |
| Sarah-2022^[22]^ | Lorlatinib | Chemotherapy | Not included | Not included | Not included | Not included | Not included | Not included | Not included | 0.22[0.15-0.32] (0.687/RE) |

*, compared to crizotinib for first-line and chemotherapy for the second-line, month; #, compared to crizotinib for first-line and chemotherapy for the second-line, predicted by FP model, year; †, compared to crizotinib using Cox-PH model; &, Asian-subgroup data was used for Asia specific NMA; $, Asian-subgroup survival curves was unavailable.

# Table 3 Network of CNS PFS for first-line treatments on global patients with or without baseline brain metastasis

| Study | Arm 1 | Arm 2 | Network of CNS PFS for first-line treatments on global patients with baseline brain metastasis | | | | Network of CNS PFS for first-line treatments on global patients without baseline brain metastasis | | | |
| --- | --- | --- | --- | --- | --- | --- | --- | --- | --- | --- |
|  |  |  | RMSD [95% CI] (mean ranking in the NMA)^*^ | Progressin-free life-years gain in 5 years (ranking in the NMA)^#^ | Progressin-free life-years gain in 10 years (ranking in the NMA)^#^ | HR [95% CI] (P-score in the NMA)^†^ | RMSD [95% CI] (mean ranking in the NMA)^*^ | Progressin-free life-years gain in 10 years (ranking in the NMA)# | Progressin-free life-years gain in 10 years (ranking in the NMA)^#^ | HR [95% CI] (P-score in the NMA)^†^ |
| ALEX^&[23]^ | Alectinib | Crizotinib | 4.12[2.29-5.91] (2.874) / 0(4.046) | 2.48(2) / 0(4) | 5.16(2) / 0(4) | 0.18[0.09-0.36] (0.762/ 0.241) | 5.63[3.73-7.55] (1.575) / 0(5.025) | 1.43(2) / 0(5) | 4.40(2) / 0(5) | 0.20[0.12-0.33] (0.795/0.245) |
| CROWN^&[10]^ | Lorlatinib | Crizotinib | 6.86[4.74-8.95] (1.290) / RE | 3.21(1) / RE | 7.09(1) / RE | 0.10[0.04-0.27] (0.951/ RE) | 5.33[3.31-7.23] (1.794) / RE | 1.61(1) / RE | 5.11(1) / RE | 0.02[0.12-0.33] (0.989/RE) |
| ALTA-1L^&[24]^ | Brigatinib | Crizotinib | 5.91[3.84-8.00] (1.836) / RE | 1.52(3) / RE | 1.84(3) / RE | 0.31[0.17-0.56] (0.537/ RE) | 0.97[-1.41-3.33] (3.837) / RE | 0.46(4) / RE | 1.33(4) / RE | 0.78[0.41-1.48] (0.355/RE) |
| eXalt3^&[6]^ | Ensartinib | Crizotinib | Not included | Not included | Not included | Not included | 4.16[2.10-6.17] (2.659) / RE | 1.13(3) / RE | 3.12(3) / RE | 0.32[0.16-0.63] (0.609/RE) |
| PROFILE 1014^&[25]^ | Chemotherapy | Crizotinib | -2.44[-5.30-0.40] (4.954)/ RE | -0.70(4)/ RE | -0.71(4)/ RE | 2.22[0.94-5.27] (0.009/ RE) | -0.91[-3.40-1.56] (5.620)/ RE | -0.94(6)/ RE | -1.55(6)/ RE | 1.45[1.24-1.69] (0.007/RE) |
| ASCEND-4^&^ | Ceritinib | Chemotherapy | Not included | Not included | Not included | Not included | Not included | Not included | Not included | Not included |
| PROFILE 1029 | Crizotinib | Chemotherapy | Not included | Not included | Not included | Not included | Not included | Not included | Not included | Not included |
| J-ALEX | Low-dose alectinib | Crizotinib | Not included | Not included | Not included | Not included | Not included | Not included | Not included | Not included |
| ALESIA | Alectinib | Crizotinib | Not included | Not included | Not included | Not included | Not included | Not included | Not included | Not included |
| TQ-B3139 | Envonalkib | Crizotinib | Not included | Not included | Not included | Not included | Not included | Not included | Not included | Not included |
| ALUR | Alectinib | Chemotherapy | Not included | Not included | Not included | Not included | Not included | Not included | Not included | Not included |
| ASCEND-5 | Ceritinib | Chemotherapy | Not included | Not included | Not included | Not included | Not included | Not included | Not included | Not included |
| ALTA-3 | Brigatinib | Alectinib | Not included | Not included | Not included | Not included | Not included | Not included | Not included | Not included |
| Profile 1007 | Crizotinib | Chemotherapy | Not included | Not included | Not included | Not included | Not included | Not included | Not included | Not included |
| Sarah-2022 | Lorlatinib | Chemotherapy | Not included | Not included | Not included | Not included | Not included | Not included | Not included | Not included |

*, compared to crizotinib, month; #, compared to crizotinib, predicted by FP model, year；†, compared to crizotinib using Cox-PH model; &, CNS-subgroup data was used for CNS specific NMA.

# Table 4 Network of PFS for first-line treatments on global patients with or without baseline brain metastasis

| Study | Arm 1 | Arm 2 | Network of PFS for first-line treatments on gloabl patients with baseline brain metastasis | | | | Network of PFS for first-line treatments on gloabl patients without baseline brain metastasis | | | |
| --- | --- | --- | --- | --- | --- | --- | --- | --- | --- | --- |
|  |  |  | RMSD [95% CI] (mean ranking in the NMA)^*^ | Life-years gain in 5 years (ranking in the NMA)^#^ | Life-years gain in 10 years (ranking in the NMA)^#^ | HR [95% CI] (P-score in the NMA)^†^ | RMSD [95% CI] (mean ranking in the NMA)^*^ | Progressin-free life-years gain in 5 years (ranking in the NMA)^#^ | Progressin-free life-years gain in 10 years (ranking in the NMA)^#^ | HR [95% CI] (P-score in the NMA)^†^ |
| ALEX^&[26]^ | Alectinib | Crizotinib | 3.01[0.77-5.25] (3.349) / 0(4.641) | 0.79(3) / 0(5) | 1.21(3) / 0(5) | 0.35[0.22-0.56] (0.695/ 0.290) | 1.30[-0.14-2.75] (3.366) / 0(5.261) | 1.15(2) / 0(5) | 2.76(2) / 0(5) | 0.47[0.32-0.70] (0.710/ 0.263) |
| CROWN^&[27]^ | Lorlatinib | Crizotinib | 6.65[4.05-9.26] (1.705) / RE | 2.12(1) / RE | 3.73(1) / RE | 0.20[0.10-0.41] (0.926/ RE) | 2.95[1.59-4.32] (1.412) / RE | 1.94(1) / RE | 4.87(1) / RE | 0.32[0.20-0.50] (0.935/ RE) |
| ALTA-1L^&[28]^ | Brigatinib | Crizotinib | 7.46[5.09-9.84] (1.333) / RE | 1.59(2) / RE | 1.96(2) / RE | 0.25[0.14-0.45] (0.849/ RE) | 0.94[-0.66-2.54] (3.837) / RE | 0.81(3) / RE | 1.71(3) / RE | 0.62[0.43-0.90] (0.537/ RE) |
| eXalt3^&[6]^ | Ensartinib | Crizotinib | 2.33[-0.45-5.13] (3.714) / RE | 0.62(4) / RE | 0.68(4) / RE | 0.55[0.30-1.01] (0.518/ RE) | 4.34[1.94-6.76] (3.339) / RE | 1.10(4) / RE | 1.59(4) / RE | 0.40[0.23-0.70] (0.807/ RE) |
| PROFILE 1014^&[7, 8]^ | Chemotherapy | Crizotinib | -4.47[-7.15~-1.74] (6.186)/ RE | -0.57(7)/ RE | -0.57(7)/ RE | 1.75[1.08-2.86] (0.014/ RE) | -2.87[-4.33~-1.37] (6.999)/ RE | -0.99(7)/ RE | -1.13(7)/ RE | 2.17[1.60-2.96] (0.000/ RE) |
| ASCEND-4^&[9]^ | Ceritinib | Chemotherapy | -1.96[-5.56, 1.66] (2.886) / RE | -0.43(6)/ RE | -0.43(6)/ RE | 1.23[0.62-2.41] (0.209/ RE) | -0.20[-2.27-1.89] (5.227) / RE | -0.58(6)/ RE | -0.72(6)/ RE | 1.04[0.65-1.67] (0.248/ RE) |
| PROFILE 1029 | Crizotinib | Chemotherapy | Not included | Not included | Not included | Not included | Not included | Not included | Not included | Not included |
| J-ALEX | Low-dose alectinib | Crizotinib | Not included | Not included | Not included | Not included | Not included | Not included | Not included | Not included |
| ALESIA | Alectinib | Crizotinib | Not included | Not included | Not included | Not included | Not included | Not included | Not included | Not included |
| TQ-B3139 | Envonalkib | Crizotinib | Not included | Not included | Not included | Not included | Not included | Not included | Not included | Not included |
| ALUR | Alectinib | Chemotherapy | Not included | Not included | Not included | Not included | Not included | Not included | Not included | Not included |
| ASCEND-5 | Ceritinib | Chemotherapy | Not included | Not included | Not included | Not included | Not included | Not included | Not included | Not included |
| ALTA-3 | Brigatinib | Alectinib | Not included | Not included | Not included | Not included | Not included | Not included | Not included | Not included |
| Profile 1007 | Crizotinib | Chemotherapy | Not included | Not included | Not included | Not included | Not included | Not included | Not included | Not included |
| Sarah-2022 | Lorlatinib | Chemotherapy | Not included | Not included | Not included | Not included | Not included | Not included | Not included | Not included |

*, compared to crizotinib, month; #, compared to crizotinib, predicted by FP model, year；†, compared to crizotinib using Cox-PH model.

# Table 5 Network of patient report outcomes

| Study | Arm 1 | Arm 2 | Network of QLQ-LC13 for first-line treatments on gloabl patients | | | | Network of QLQ-C30 for first-line treatments on gloabl patients | | | |
| --- | --- | --- | --- | --- | --- | --- | --- | --- | --- | --- |
|  |  |  | RMSD [95% CI] (mean ranking in the NMA)* | Life-years gain in 5 years (ranking in the NMA)# | Life-years gain in 10 years (ranking in the NMA)# | HR [95% CI] (P-score in the NMA)† | RMSD [95% CI] (mean ranking in the NMA)* | Life-years gain in 5 years (ranking in the NMA)# | Life-years gain in 10 years (ranking in the NMA)# | HR [95% CI] (P-score in the NMA)† |
| ALEX^[29]^ | Alectinib | Crizotinib | -0.88[-2.62-0.86] (4.490) / 0(3.166) | 0.20(2) / 0(5) | 0.31(2) / 0(5) | 1.10[0.72-1.68] (0.399/ 0.514) | Not included | Not included | Not included | 0.72[0.38-1.38] (0.741/ 0.335) |
| CROWN^[30]^ | Lorlatinib | Crizotinib | -0.16[-1.77-1.43] (3.458) / RE | 0.15(4) / RE | 0.17(4) / RE | 1.09[0.82-1.44] (0.386/ RE) | 1.24[-2.17-4.63] (2.109) / 0(2.752) | 0.34(2) / 0(3) | 1.13(2) / 0(3) | 0.92[0.65-1.30] (0.483/ RE) |
| ALTA-1L^[31]^ | Brigatinib | Crizotinib | 1.28[-0.85-3.40] (1.638) / RE | 0.41(1) / RE | 0.49(1) / RE | 0.81[0.55-1.20] (0.760/ RE) | 4.04[0.64-7.46] (1.139) / RE | 0.79(1) / RE | 2.16(1) / RE | 0.70[0.49-1.00] (0.837/ RE) |
| eXalt3^[6]^ | Ensartinib | Crizotinib | Not included | Not included | Not included | Not included | Not included | Not included | Not included | 0.83[0.57-1.20] (0.105/ RE) |
| PROFILE 1014^[7, 8]^ | Chemotherapy | Crizotinib | -2.15[-3.63~-0.67] (6.186)/ RE | -0.36(6)/ RE | -0.37(6)/ RE | 1.85[1.50-2.28] (0.004/ RE) | Not included | Not included | Not included | Not included |
| ASCEND-4^[9]^ | Ceritinib | Chemotherapy | 0.54[-1.49-2.51] (2.43) / RE | 0.17(3)/ RE | 0.24(3)/ RE | 0.63[0.39-1.01] (0.939/ RE) | Not included | Not included | Not included | Not included |
| PROFILE 1029 | Crizotinib | Chemotherapy | Not included | Not included | Not included | Not included | Not included | Not included | Not included | Not included |
| J-ALEX | Low-dose alectinib | Crizotinib | Not included | Not included | Not included | Not included | Not included | Not included | Not included | Not included |
| ALESIA | Alectinib | Crizotinib | Not included | Not included | Not included | Not included | Not included | Not included | Not included | Not included |
| TQ-B3139 | Envonalkib | Crizotinib | Not included | Not included | Not included | Not included | Not included | Not included | Not included | Not included |
| ALUR | Alectinib | Chemotherapy | Not included | Not included | Not included | Not included | Not included | Not included | Not included | Not included |
| ASCEND-5 | Ceritinib | Chemotherapy | Not included | Not included | Not included | Not included | Not included | Not included | Not included | Not included |
| ALTA-3 | Brigatinib | Alectinib | Not included | Not included | Not included | Not included | Not included | Not included | Not included | Not included |
| Profile 1007 | Crizotinib | Chemotherapy | Not included | Not included | Not included | Not included | Not included | Not included | Not included | Not included |
| Sarah-2022 | Lorlatinib | Chemotherapy | Not included | Not included | Not included | Not included | Not included | Not included | Not included | Not included |

*, compared to crizotinib, month; #, compared to crizotinib, predicted by FP model, year；†, compared to crizotinib using Cox-PH model.

Table 6 Network of PFS for other subgroups

| Study | Arm 1 | Arm 2 | Network of PFS for subgroup analysis of first-line treatments on gloabl patients based on time-invarient Cox-PH model^*^, HR [95% CI] (P-score) | | | | | | | | |
| --- | --- | --- | --- | --- | --- | --- | --- | --- | --- | --- | --- |
|  |  |  | Non-Asian | Smoker | Non-smoker | Age less than 65 | Age over 65 | Male | Female | ECOG 0 or 1 | ECOG 2 |
| ALEX | Alectinib | Crizotinib | 0.44[0.30-0.65] (0.765/ 0.217) | 0.48[0.30-0.77] (0.743/ 0.236) | 0.40[0.27-0.59] (0.688/ 0.314) | 0.45[0.32-0.63] (0.635/ 0.287) | 0.40[0.21-0.76] (0.830/ 0.335) | 0.55[0.36-0.85] (0.577/ 0.198) | 0.38[0.25-0.58] (0.760/ 0.182) | Not included | 0.74[0.25-2.17] (0.848/ 0.642) |
| CROWN | Lorlatinib | Crizotinib | 0.19[0.11-0.32] (0.998/ RE) | 0.36[0.20-0.64] (0.909/ 0.236) | 0.24[0.14-0.41] (0.966/ RE) | 0.22[0.13-0.37] (0.989/ RE) | 0.35[0.20-0.63] (0.899/ RE) | 0.26[0.16-0.43] (0.958/ RE) | 0.31[0.18-0.54] (0.937/ RE) | 0.28[0.19-0.42] (0.980/ 0.333) | Not included |
| ALTA-1L | Brigatinib | Crizotinib | 0.56[0.38-0.83] (0.611/ RE) | 0.48[0.29-0.80] (0.742/ RE) | 0.43[0.28-0.66] (0.639/ RE) | 0.42[0.29-0.61] (0.688/ RE) | 0.58[0.33-1.01] (0.635/ RE) | 0.47[0.30-0.73] (0.624/ RE) | 0.48[0.30-0.76] (0.678/ RE) | Not included | Not included |
| eXalt3 | Ensartinib | Crizotinib | 0.61[0.34-1.10] (0.553/ RE) | 0.78[0.39-1.56] (0.415/ RE) | 0.39[0.23-0.66] (0.707/ RE) | 0.42[0.27-0.65] (0.688/ RE) | Not included | 0.45[0.25-0.81] (0.658/ RE) | 0.44[0.25-0.77] (0.736/ RE) | 0.44[0.29-0.66] (0.686/ RE) | Not included |
| PROFILE 1014 | Chemotherapy | Crizotinib | 1.89[1.30-2.74] (0.002/ RE) | 1.56[1.03-2.37] (0.011/ RE) | 2.44[1.72-3.45] (0.002/ RE) | 1.96[1.47-2.62] (0.000/ RE) | 2.70[1.27-5.75] (0.003/ RE) | 2.22[1.58-3.12] (0.000/ RE) | 1.85[1.23-2.79] (0.000/ RE) | Not included | 5.26[1.35-20.52] (0.011/ RE) |
| ASCEND-4 | Ceritinib | Chemotherapy | 0.83[0.48-1.43] (0.355/ RE) | 0.75[0.40-1.41] (0.444/ RE) | 1.37[0.82-2.27] (0.186/ RE) | 1.14[0.74-1.76] (0.214/ RE) | 1.22[0.45-3.27] (0.298/ RE) | 1.40[0.84-2.34] (0.182/ RE) | 0.76[0.42-1.37] (0.373/ RE) | Not included | Not included |
| PROFILE 1029 | Crizotinib | Chemotherapy | Not included | Not included | Not included | Not included | Not included | Not included | Not included | Not included | Not included |
| J-ALEX | Low-dose alectinib | Crizotinib | Not included | Not included | Not included | Not included | Not included | Not included | Not included | Not included | Not included |
| ALESIA | Alectinib | Crizotinib | Not included | Not included | Not included | Not included | Not included | Not included | Not included | Not included | Not included |
| TQ-B3139 | Envonalkib | Crizotinib | Not included | Not included | Not included | Not included | Not included | Not included | Not included | Not included | Not included |
| ALUR | Alectinib | Chemotherapy | Not included | Not included | Not included | Not included | Not included | Not included | Not included | Not included | Not included |
| ASCEND-5 | Ceritinib | Chemotherapy | Not included | Not included | Not included | Not included | Not included | Not included | Not included | Not included | Not included |
| ALTA-3 | Brigatinib | Alectinib | Not included | Not included | Not included | Not included | Not included | Not included | Not included | Not included | Not included |
| Profile 1007 | Crizotinib | Chemotherapy | Not included | Not included | Not included | Not included | Not included | Not included | Not included | Not included | Not included |
| Sarah-2022 | Lorlatinib | Chemotherapy | Not included | Not included | Not included | Not included | Not included | Not included | Not included | Not included | Not included |

*,compared to crizotinib；

Table 7 Network of Adverse events

| Study | Arm 1 | Arm 2 | Network of Adverse events for first-line treatments, Log OR [95% CI] (ranking) | | | Network of Adverse event for second-line treatments, Log OR [95% CI] (ranking) | |
| --- | --- | --- | --- | --- | --- | --- | --- |
|  |  |  | Any-grade | Grade 3-4 | Grade 5 | Any-grade | Grade 3-4 |
| ALEX | Alectinib | Crizotinib | -0.24[-1.71-1.17] (3) / 0(6) | -0.21[-0.57-0.15] (2) / 0(3) | -0.07[-0.95-0.85] (3) / 0(4) | Not included | Not included |
| CROWN | Lorlatinib | Crizotinib | 25.63[2.02-85.01] (7) / RE | 0.87[0.37-1.38] (8)/ RE | 0.33[-0.67-1.38] (6)/ RE | Not included | Not included |
| ALTA-1L | Brigatinib | Crizotinib | -0.01[-3.63-3.66] (5) / RE | 0.59[0.10-1.09] (6) / RE | 0.01[-0.87-0.89] (5)/ RE | Not included | Not included |
| eXalt3 | Ensartinib | Crizotinib | -0.91[-4.36-1.66] (2) / RE | Not included | -1.68[-5.07-0.42] (2)/ RE | Not included | Not included |
| PROFILE 1014 | Chemotherapy | Crizotinib | -0.01[-3.56-3.79] (4)/ RE | 0.80[0.34-1.26] (4)/ RE | -1.78[-3.07~-0.75] (1)/ RE | Not included | Not included |
| ASCEND-4 | Ceritinib | Chemotherapy | 30.74[2.47-70.01] (8) / RE | 0.12[-0.32-0.55] (4)/ RE | Not included | Not included | Not included |
| PROFILE 1029 | Crizotinib | Chemotherapy | Not included | Not included | Not included | Not included | Not included |
| J-ALEX | Low-dose alectinib | Crizotinib | -20.46[-58.03~-1.79] (1) / RE | -1.12[-1.72~-0.53] (1) / RE | Not included | Not included | Not included |
| ALESIA | Alectinib | Crizotinib | RE/RE | RE/RE | RE/RE | Not included | Not included |
| TQ-B3139 | Envonalkib | Crizotinib | Not included | 0.46[-0.02-0.95] (5) / RE | Not included | Not included | Not included |
| ALUR | Alectinib | Chemotherapy | Not included | Not included | Not included | 0.00[-1.28-1.45] (2) / 0(1) | -0.24[-1.03-0.56] (1) / 0(2) |
| ASCEND-5 | Ceritinib | Chemotherapy | Not included | Not included | Not included | 0.47[-0.03-2.01] (4) / RE | Not included |
| ALTA-3 | Brigatinib | Alectinib | Not included | Not included | Not included | 0.32[-1.39-1.87] (3) / RE | 1.04[0.06-2.03] (3) / RE |
| Profile 1007 | Crizotinib | Chemotherapy | Not included | Not included | Not included | Not included | Not included |
| Sarah-2022 | Lorlatinib | Chemotherapy | Not included | Not included | Not included | Not included | Not included |

Table 8 Network of Objective Response Rate

| Study | Arm 1 | Arm 2 | Network of ORR, Log OR [95% CI] (ranking) | | | |
| --- | --- | --- | --- | --- | --- | --- |
|  |  |  | First-line systemic ORR | First-line CNS ORR | Second-line systemic ORR | Second-line CNS ORR |
| ALEX | Alectinib | Crizotinib | 0.65[0.18-1.14] (4) / 0(8) | 1.86[0.95-2.80] (5) / 0(7) | Not included | Not included |
| CROWN | Lorlatinib | Crizotinib | 0.88[0.38-1.39] (2) / RE | 2.20[1.19-3.36] (3)/ RE | Not included | Not included |
| ALTA-1L | Brigatinib | Crizotinib | 0.25[-0.28-0.78] (7) / RE | 2.52[1.54-3.59] (2) / RE | Not included | Not included |
| eXalt3 | Ensartinib | Crizotinib | 0.36[-0.14-0.87] (6) / 0(8) | 2.02[0.34-3.90] (3) / RE | Not included | Not included |
| PROFILE 1014 | Chemotherapy | Crizotinib | -1.24[-1.71~-0.78] (9)/ RE | -1.41[-2.44~-0.43] (8)/ RE | Not included | Not included |
| ASCEND-4 | Ceritinib | Chemotherapy | 0.75[0.11-1.41] (3) / RE | 0.65[-1.01-2.38] (6)/ RE | Not included | Not included |
| PROFILE 1029 | Crizotinib | Chemotherapy | Not included due to heterogeneity | Not included | Not included | Not included |
| J-ALEX | Low-dose alectinib | Crizotinib | 1.11[0.19-2.10] (1) / RE | Not included | Not included | Not included |
| ALESIA | Alectinib | Crizotinib | RE/RE | RE/RE | Not included | Not included |
| TQ-B3139 | Envonalkib | Crizotinib | 0.65[0.08-1.25] (5) / 0(8) | 2.57[1.50-3.73] (1) / RE | Not included | Not included |
| ALUR | Alectinib | Chemotherapy | Not included | Not included | 4.03[2.34-7.23] (1) / 0(4) | 25.96[6.46-61.88] (2) / 0(4) |
| ASCEND-5 | Ceritinib | Chemotherapy | Not included | Not included | 2.19[1.42-3.13] (3) / RE | 2.70[0.58-6.53] (3) / RE |
| ALTA-3 | Brigatinib | Alectinib | Not included | Not included | 3.68[1.89-6.91] (2) / RE | 26.20[6.70-62.10] (3) / RE |
| Profile 1007 | Crizotinib | Chemotherapy | Not included | Not included | Not included | Not included |
| Sarah-2022 | Lorlatinib | Chemotherapy | Not included | Not included | Not included | Not included |

**REFERENCE**

[1] Peters S, Camidge D R, Shaw A T, et al. Alectinib versus Crizotinib in Untreated ALK-Positive Non-Small-Cell Lung Cancer[J]. N Engl J Med, 2017,377(9):829-838.

[2] Mok T, Camidge D R, Gadgeel S M, et al. Updated overall survival and final progression-free survival data for patients with treatment-naive advanced ALK-positive non-small-cell lung cancer in the ALEX study[J]. Ann Oncol, 2020,31(8):1056-1064.

[3] Wolf J, Helland A, Oh I J, et al. Final efficacy and safety data, and exploratory molecular profiling from the phase III ALUR study of alectinib versus chemotherapy in crizotinib-pretreated ALK-positive non-small-cell lung cancer[J]. ESMO Open, 2022,7(1):100333.

[4] Shaw A T, Bauer T M, de Marinis F, et al. First-Line Lorlatinib or Crizotinib in Advanced ALK-Positive Lung Cancer[J]. N Engl J Med, 2020,383(21):2018-2029.

[5] Camidge D R, Kim H R, Ahn M J, et al. Brigatinib versus Crizotinib in ALK-Positive Non-Small-Cell Lung Cancer[J]. N Engl J Med, 2018,379(21):2027-2039.

[6] Horn L, Wang Z, Wu G, et al. Ensartinib vs Crizotinib for Patients With Anaplastic Lymphoma Kinase-Positive Non-Small Cell Lung Cancer: A Randomized Clinical Trial[J]. JAMA Oncol, 2021,7(11):1617-1625.

[7] Solomon B J, Kim D W, Wu Y L, et al. Final Overall Survival Analysis From a Study Comparing First-Line Crizotinib Versus Chemotherapy in ALK-Mutation-Positive Non-Small-Cell Lung Cancer[J]. J Clin Oncol, 2018,36(22):2251-2258.

[8] Solomon B J, Mok T, Kim D W, et al. First-line crizotinib versus chemotherapy in ALK-positive lung cancer[J]. N Engl J Med, 2014,371(23):2167-2177.

[9] Soria J C, Tan D, Chiari R, et al. First-line ceritinib versus platinum-based chemotherapy in advanced ALK-rearranged non-small-cell lung cancer (ASCEND-4): a randomised, open-label, phase 3 study[J]. Lancet, 2017,389(10072):917-929.

[10] Qing Z, Kim H R, Soo R A, et al. 992P Updated analyses from the CROWN study of first-line lorlatinib vs crizotinib in Asian patients with ALK-positive non-small cell lung cancer (NSCLC)[J]. Annals of Oncology, 2022,33:S1007.

[11] Ahn M J, Kim H R, Yang J, et al. Efficacy and Safety of Brigatinib Compared With Crizotinib in Asian vs. Non-Asian Patients With Locally Advanced or Metastatic ALK-Inhibitor-Naive ALK+ Non-Small Cell Lung Cancer: Final Results From the Phase III ALTA-1L Study[J]. Clin Lung Cancer, 2022,23(8):720-730.

[12] Nishio M, Kim D W, Wu Y L, et al. Crizotinib versus Chemotherapy in Asian Patients with ALK-Positive Advanced Non-small Cell Lung Cancer[J]. Cancer Res Treat, 2018,50(3):691-700.

[13] Tan D, Geater S, Yu C J, et al. Ceritinib Efficacy and Safety in Treatment-Naive Asian Patients With Advanced ALK-Rearranged NSCLC: An ASCEND-4 Subgroup Analysis[J]. JTO Clin Res Rep, 2021,2(3):100131.

[14] Wu Y L, Lu S, Lu Y, et al. Results of PROFILE 1029, a Phase III Comparison of First-Line Crizotinib versus Chemotherapy in East Asian Patients with ALK-Positive Advanced Non-Small Cell Lung Cancer[J]. J Thorac Oncol, 2018,13(10):1539-1548.

[15] Hotta K, Hida T, Nokihara H, et al. Final overall survival analysis from the phase III J-ALEX study of alectinib versus crizotinib in ALK inhibitor-naive Japanese patients with ALK-positive non-small-cell lung cancer[J]. ESMO Open, 2022,7(4):100527.

[16] Yang J C, Liu G, Lu S, et al. 319O ALTA-3: A randomized trial of brigatinib (BRG) vs alectinib (ALC) in crizotinib (CRZ)-refractory advanced ALK+ NSCLC[J]. Annals of Oncology, 2022,33:S1564.

[17] Zhou C, Kim S W, Reungwetwattana T, et al. Alectinib versus crizotinib in untreated Asian patients with anaplastic lymphoma kinase-positive non-small-cell lung cancer (ALESIA): a randomised phase 3 study[J]. Lancet Respir Med, 2019,7(5):437-446.

[18] Zhang L, Fang W, Min J, et al. 320MO Envonalkib vs crizotinib in treatment-naïve advanced ALK-positive NSCLC: A randomized, multicenter, phase III trial[J]. Annals of Oncology, 2022,33:S1564-S1565.

[19] Novello S, Mazieres J, Oh I J, et al. Alectinib versus chemotherapy in crizotinib-pretreated anaplastic lymphoma kinase (ALK)-positive non-small-cell lung cancer: results from the phase III ALUR study[J]. Ann Oncol, 2018,29(6):1409-1416.

[20] Shaw A T, Kim T M, Crino L, et al. Ceritinib versus chemotherapy in patients with ALK-rearranged non-small-cell lung cancer previously given chemotherapy and crizotinib (ASCEND-5): a randomised, controlled, open-label, phase 3 trial[J]. Lancet Oncol, 2017,18(7):874-886.

[21] Shaw A T, Kim D W, Nakagawa K, et al. Crizotinib versus chemotherapy in advanced ALK-positive lung cancer[J]. N Engl J Med, 2013,368(25):2385-2394.

[22] Smith S, Albuquerque D A F, Ines M, et al. Matching-Adjusted Indirect Comparisons of Lorlatinib Versus Chemotherapy for Patients With Second-Line or Later Anaplastic Lymphoma Kinase-Positive Non-Small Cell Lung Cancer[J]. Value Health, 2023,26(1):64-70.

[23] Gadgeel S, Peters S, Mok T, et al. Alectinib versus crizotinib in treatment-naive anaplastic lymphoma kinase-positive (ALK+) non-small-cell lung cancer: CNS efficacy results from the ALEX study[J]. Ann Oncol, 2018,29(11):2214-2222.

[24] Camidge D R, Kim H R, Ahn M J, et al. Brigatinib Versus Crizotinib in ALK Inhibitor-Naive Advanced ALK-Positive NSCLC: Final Results of Phase 3 ALTA-1L Trial[J]. J Thorac Oncol, 2021,16(12):2091-2108.

[25] Solomon B J, Cappuzzo F, Felip E, et al. Intracranial Efficacy of Crizotinib Versus Chemotherapy in Patients With Advanced ALK-Positive Non-Small-Cell Lung Cancer: Results From PROFILE 1014[J]. J Clin Oncol, 2016,34(24):2858-2865.

[26] Camidge D R, Dziadziuszko R, Peters S, et al. Updated Efficacy and Safety Data and Impact of the EML4-ALK Fusion Variant on the Efficacy of Alectinib in Untreated ALK-Positive Advanced Non-Small Cell Lung Cancer in the Global Phase III ALEX Study[J]. J Thorac Oncol, 2019,14(7):1233-1243.

[27] Solomon B J, Bauer T M, Mok T, et al. Efficacy and safety of first-line lorlatinib versus crizotinib in patients with advanced, ALK-positive non-small-cell lung cancer: updated analysis of data from the phase 3, randomised, open-label CROWN study[J]. Lancet Respir Med, 2022.

[28] Camidge D R, Kim H R, Ahn M J, et al. Brigatinib Versus Crizotinib in Advanced ALK Inhibitor-Naive ALK-Positive Non-Small Cell Lung Cancer: Second Interim Analysis of the Phase III ALTA-1L Trial[J]. J Clin Oncol, 2020,38(31):3592-3603.

[29] Perol M, Pavlakis N, Levchenko E, et al. Patient-reported outcomes from the randomized phase III ALEX study of alectinib versus crizotinib in patients with ALK-positive non-small-cell lung cancer[J]. Lung Cancer, 2019,138:79-87.

[30] Mazieres J, Iadeluca L, Shaw A T, et al. Patient-reported outcomes from the randomized phase 3 CROWN study of first-line lorlatinib versus crizotinib in advanced ALK-positive non-small cell lung cancer[J]. Lung Cancer, 2022,174:146-156.

[31] Garcia C M, Lin H M, Zhu Y, et al. Health-related quality of life in the randomized phase III trial of brigatinib vs crizotinib in advanced ALK inhibitor-naive ALK + non-small cell lung cancer (ALTA-1L)[J]. Lung Cancer, 2021,155:68-77.
