# Supplementary material for: Identifying optimal ALK inhibitors in first- and second-line treatment of patients with advanced ALK-positive non-small-cell lung cancer: a systematic review and network meta-analysis
Source: BMC Cancer. 2024 Feb 8;24:186. doi: 10.1186/s12885-024-11916-4 (PMC10851546; doi:10.1186/s12885-024-11916-4)
Supplement: Supplementary file 7 — Additional file 7. [file 12885_2024_11916_MOESM7_ESM.docx]

**Additional file 7**

**Part A Progression-free Survival for First-line Treatments of Global Patients Using Short-term IRC-accessed Data from ALEX**

1. **Results from RSMT model**

**
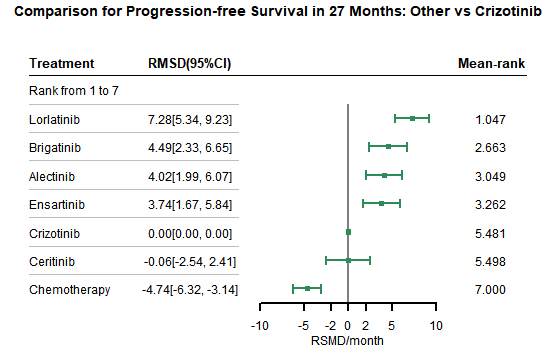
**


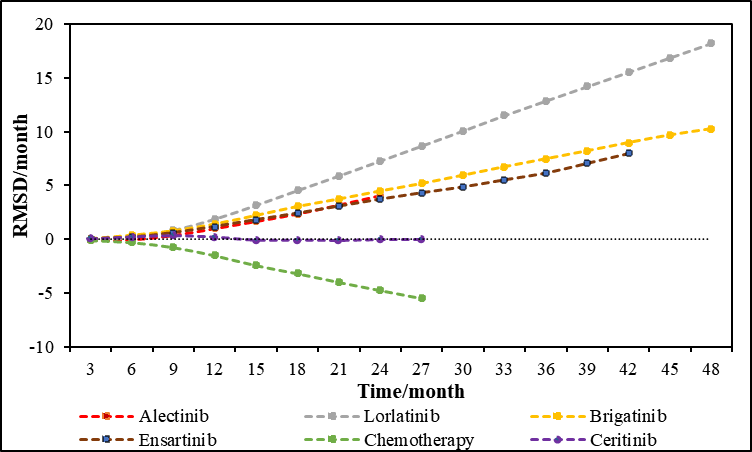


1. **Results from FP and RP model**

Survival curves predicted by FP model


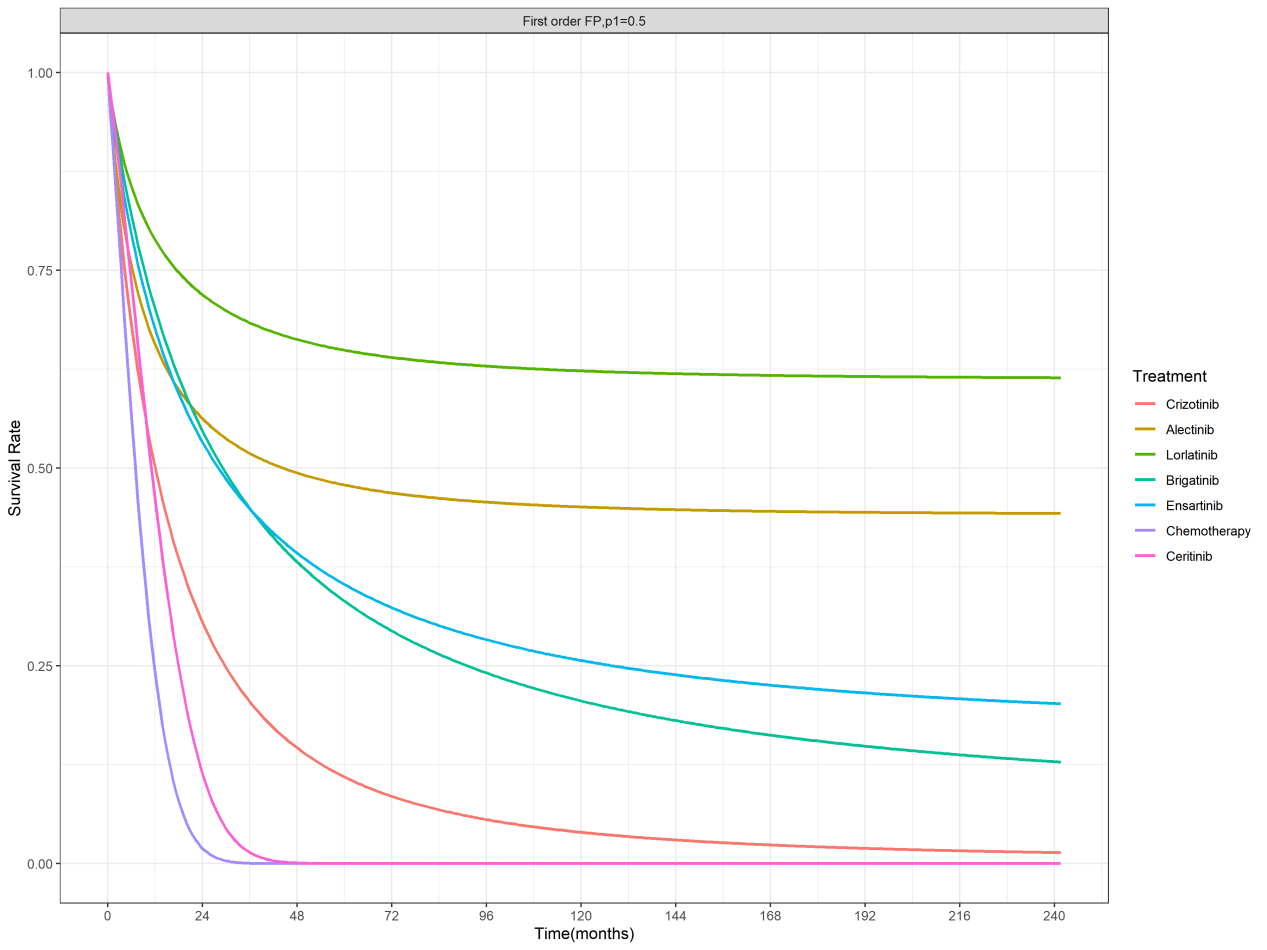


Time-varying HRs (FP model)


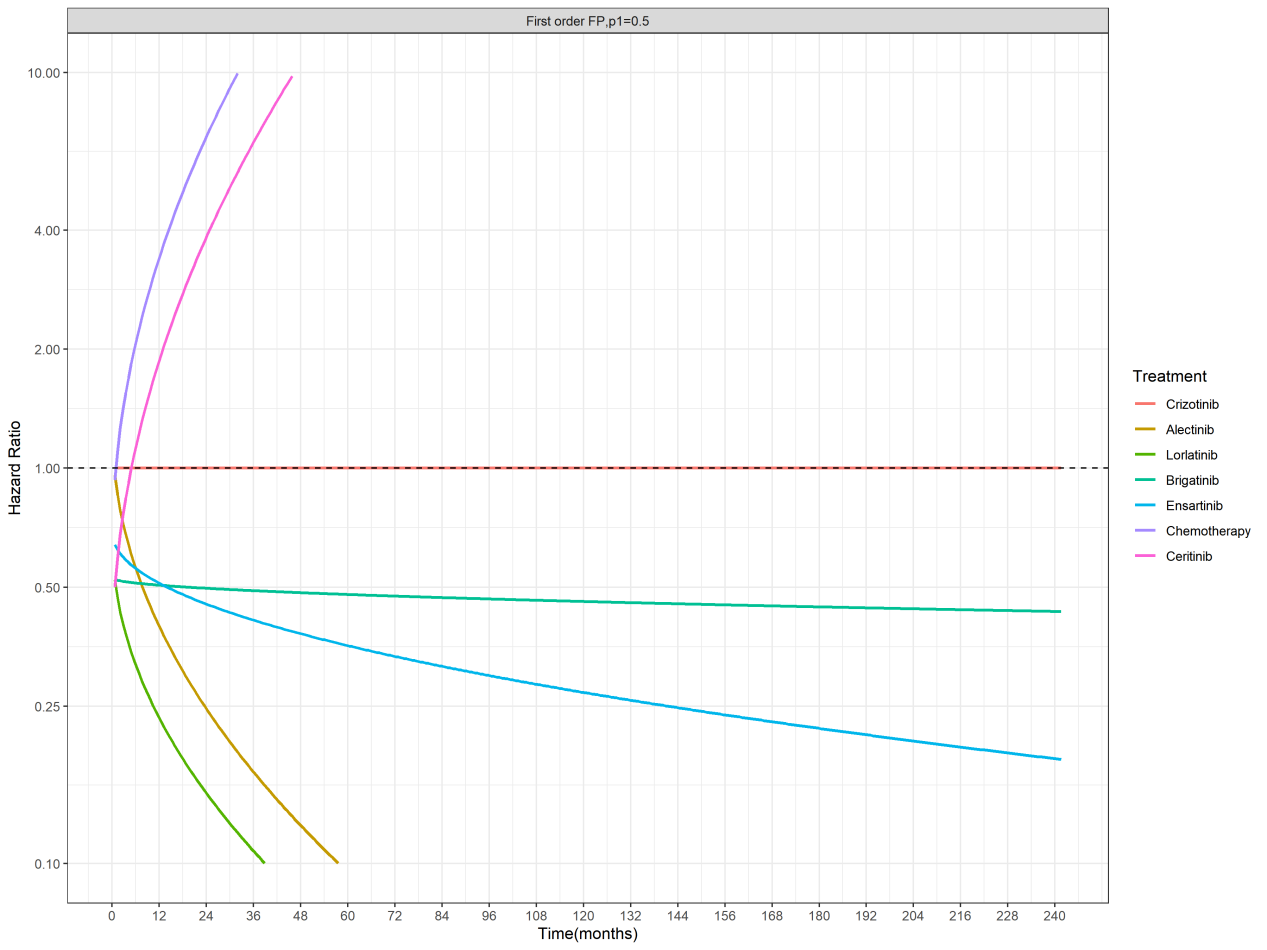


Survival curves predicted by RP model


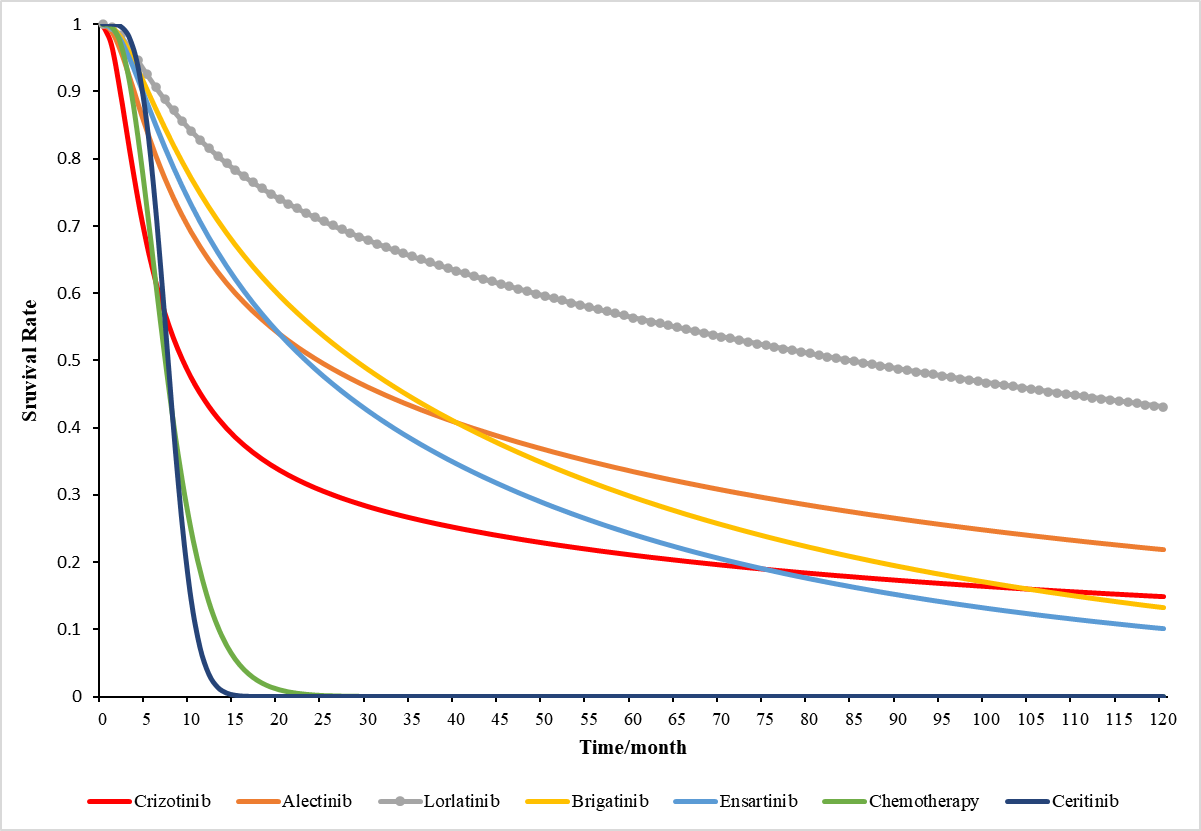


Ranking Plot (RP model)
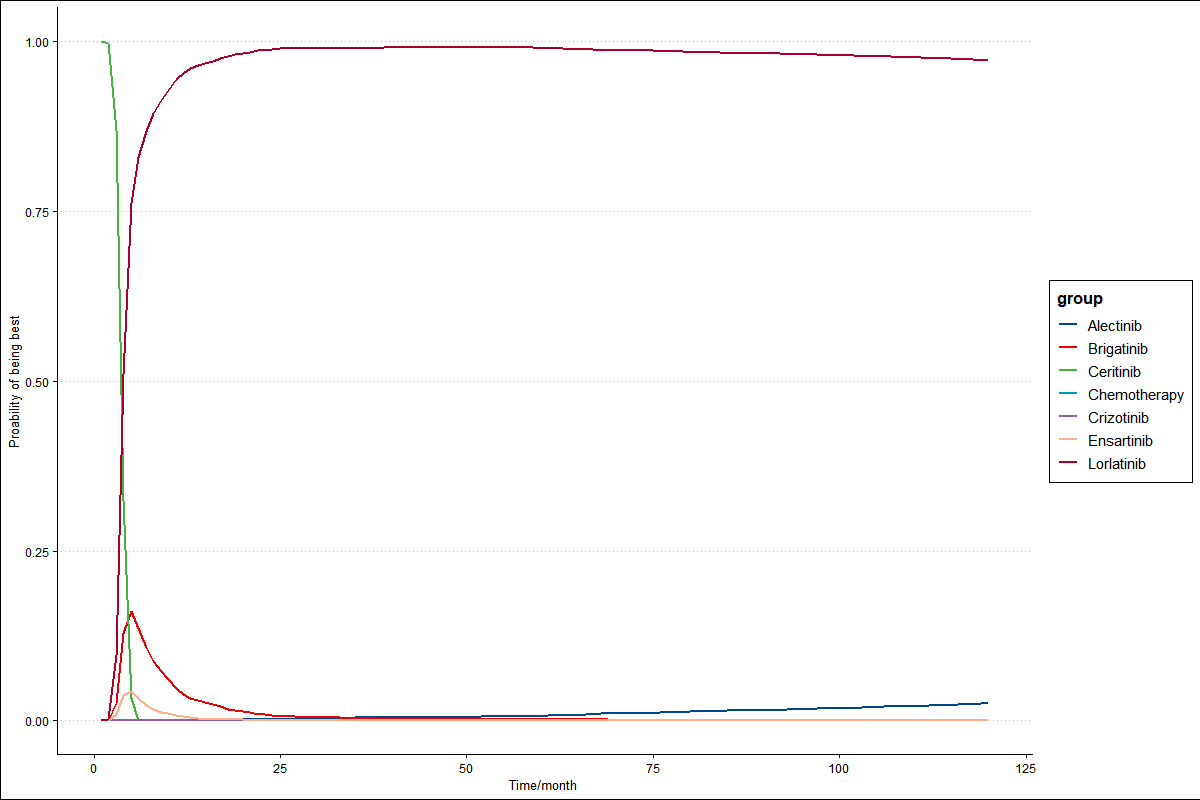


1. **Results from Cox-PH model**

**
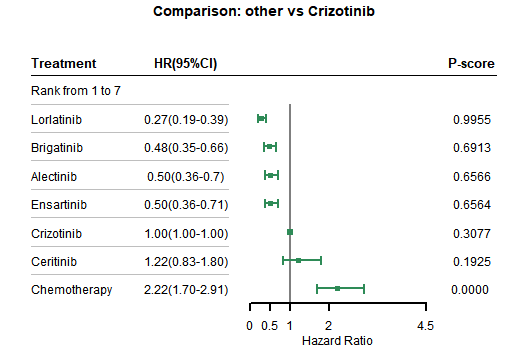
**

**Part B Progression-free Survival for First-line Treatments of Asian Patients Using Short-term IRC-accessed Data from ALESIA**

1. **Results from RSMT model**

**
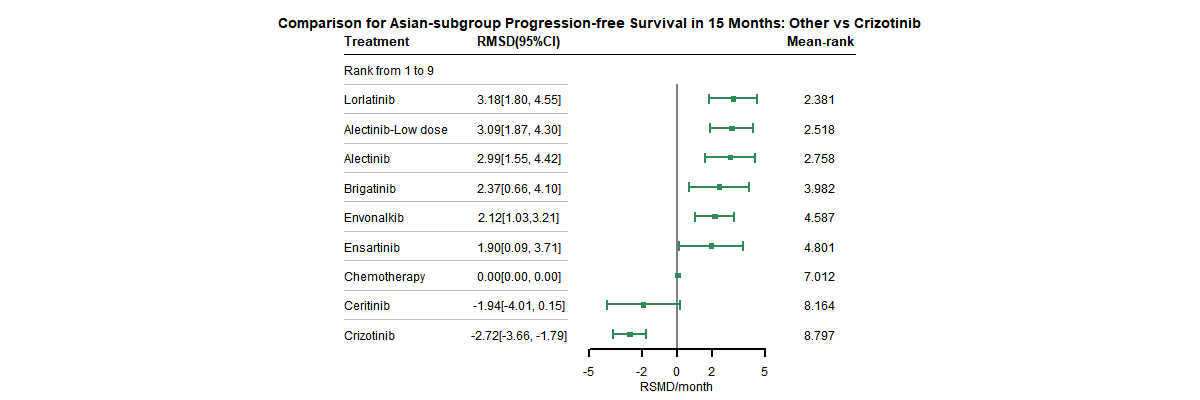
**


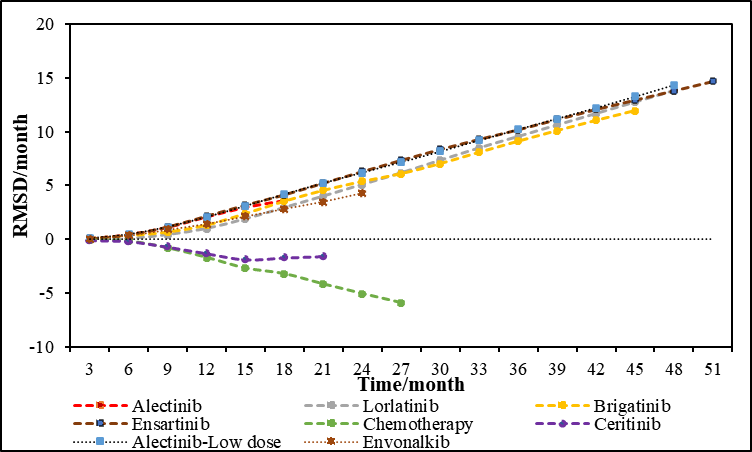


1. **Results from FP and RP model**

Survival curves predicted by FP model


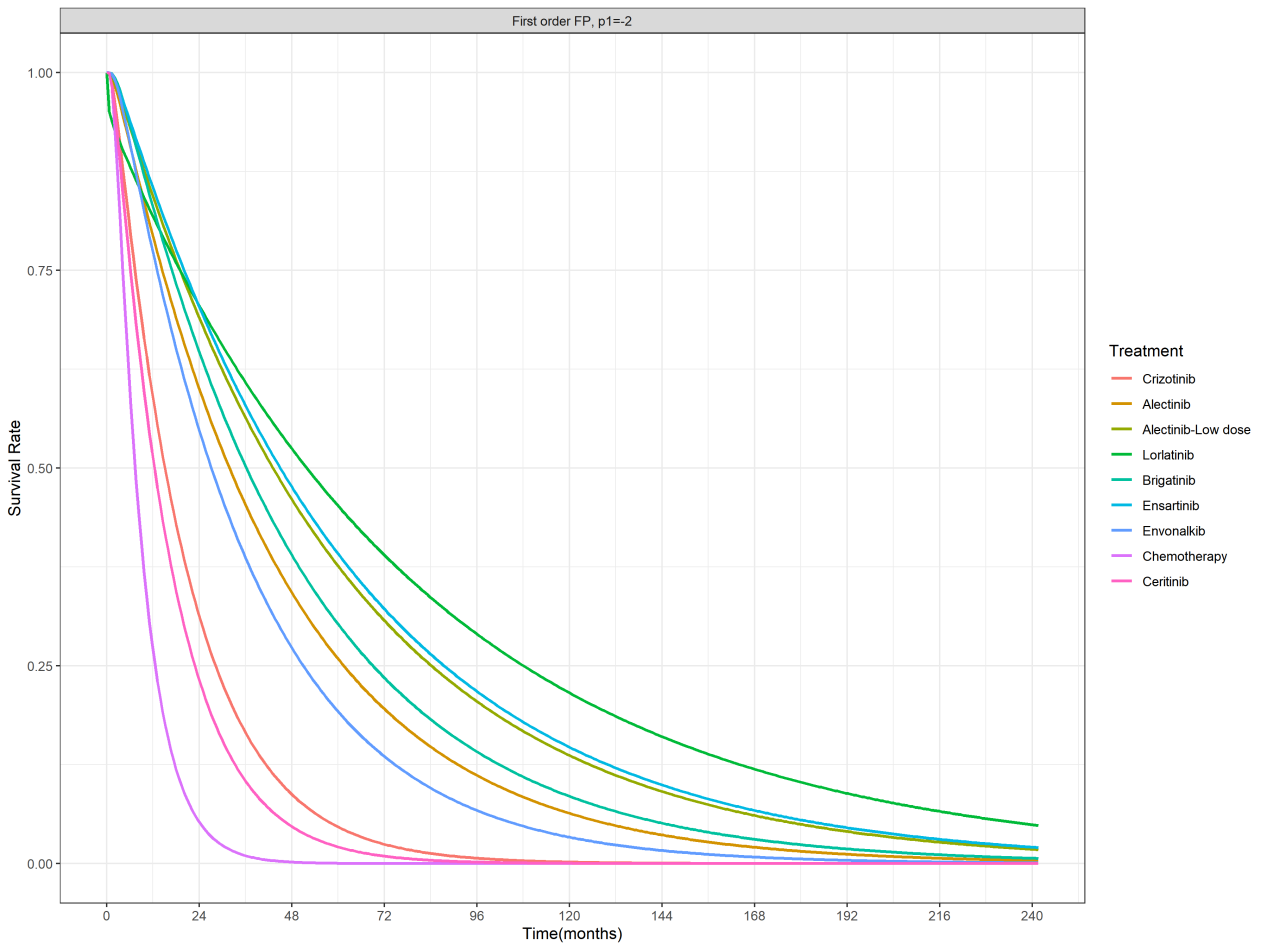


Time-varying HRs (FP model)


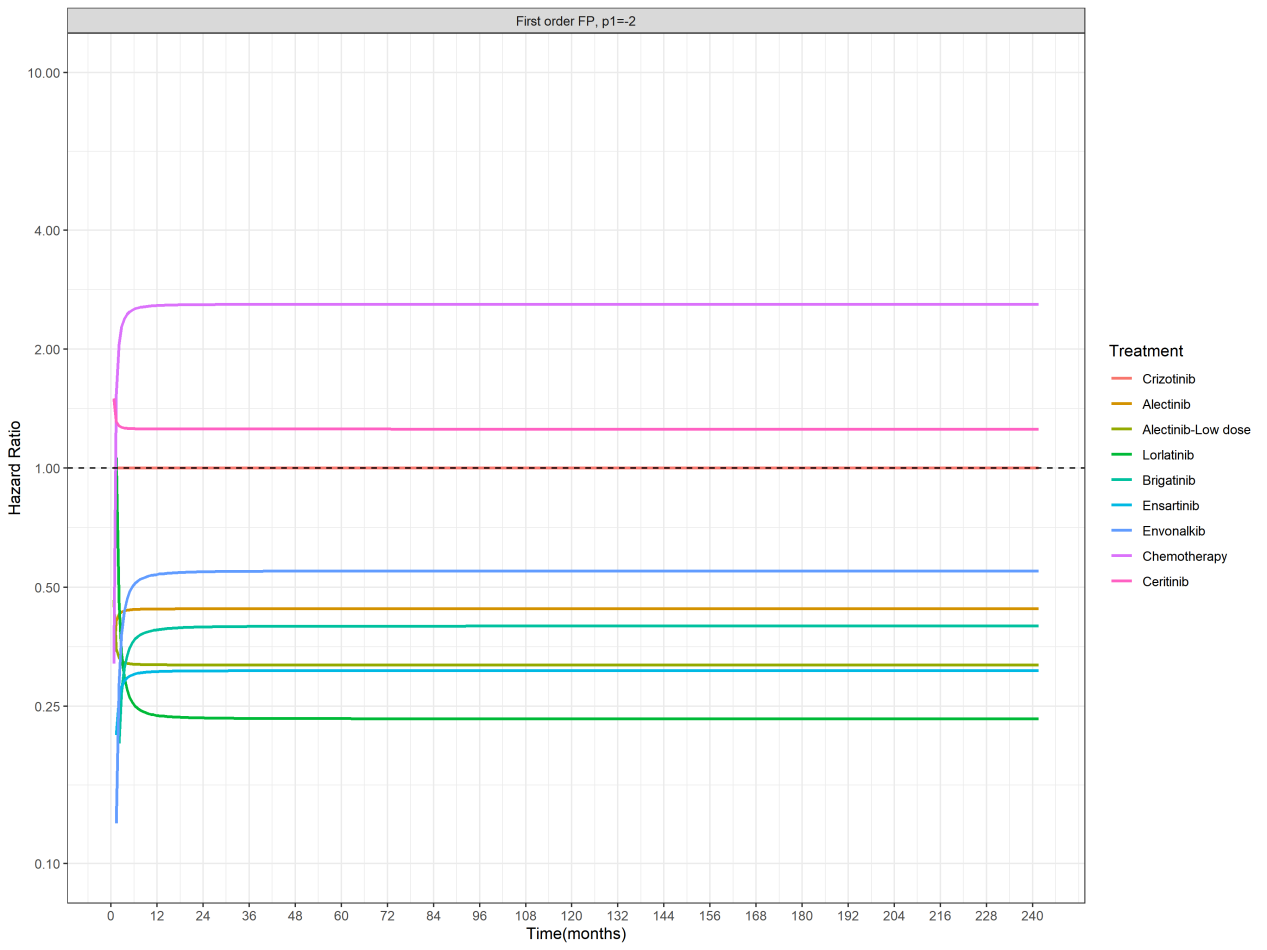


Survival curves predicted by RP model


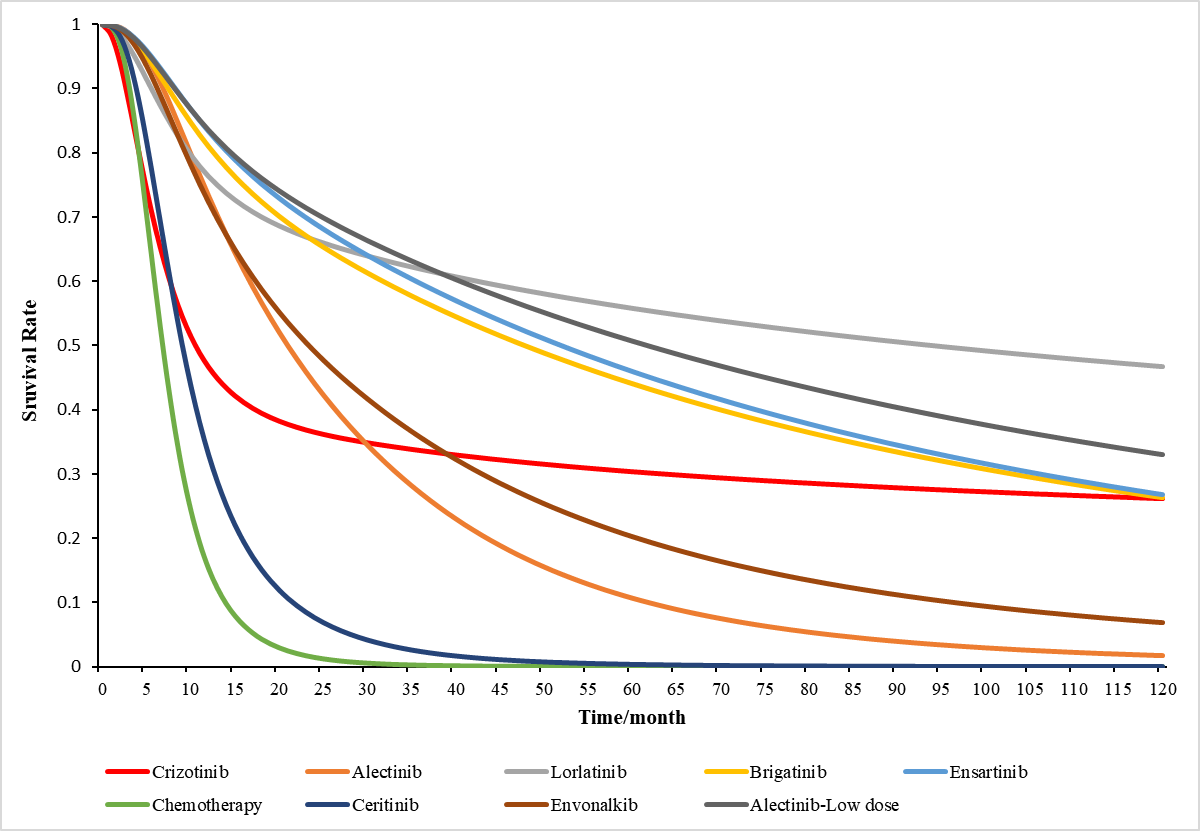


Ranking Plot (RP model)


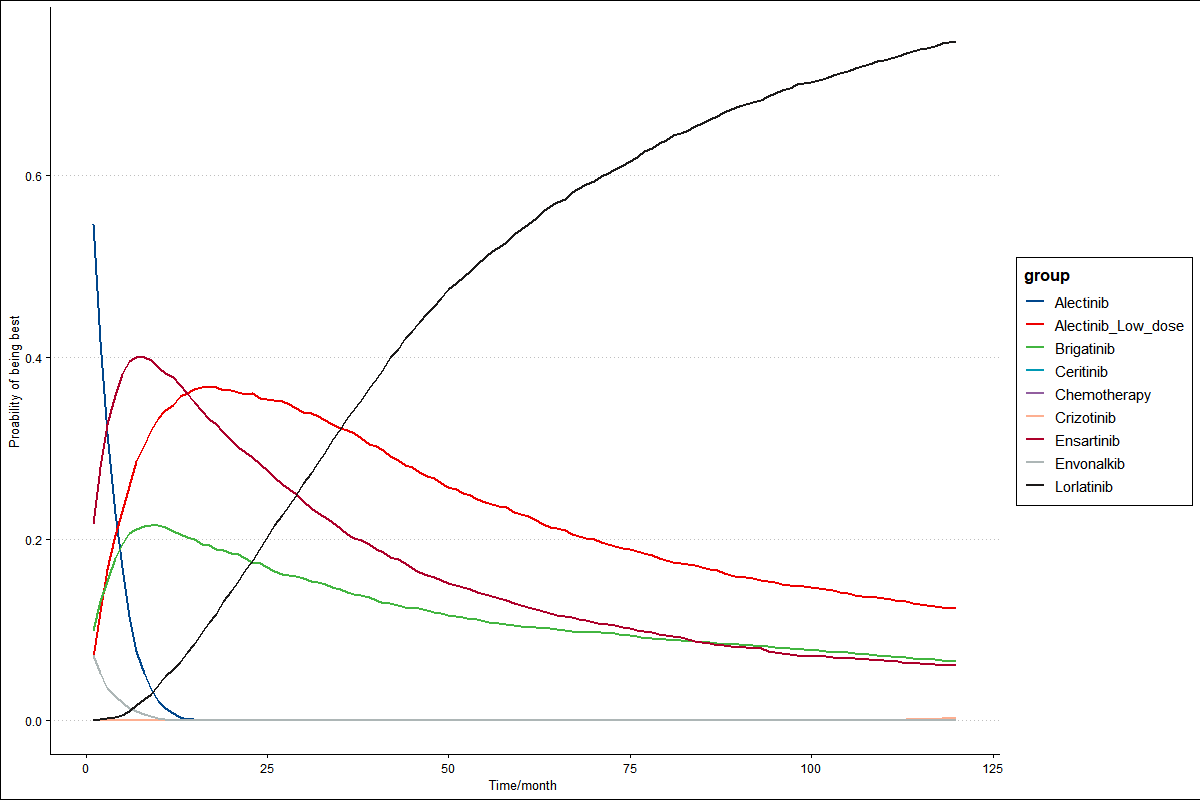


1. **Results from Cox-PH model**

**
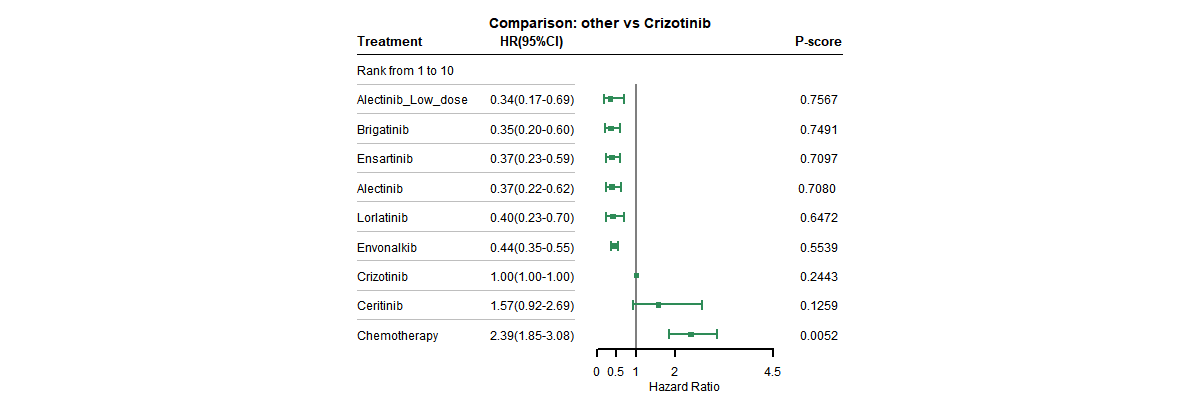
**

**Part C Progression-free Survival for Second-line Treatments Using Short-term IRC-accessed Data from ALUR**

1. **Results from RSMT model**

**
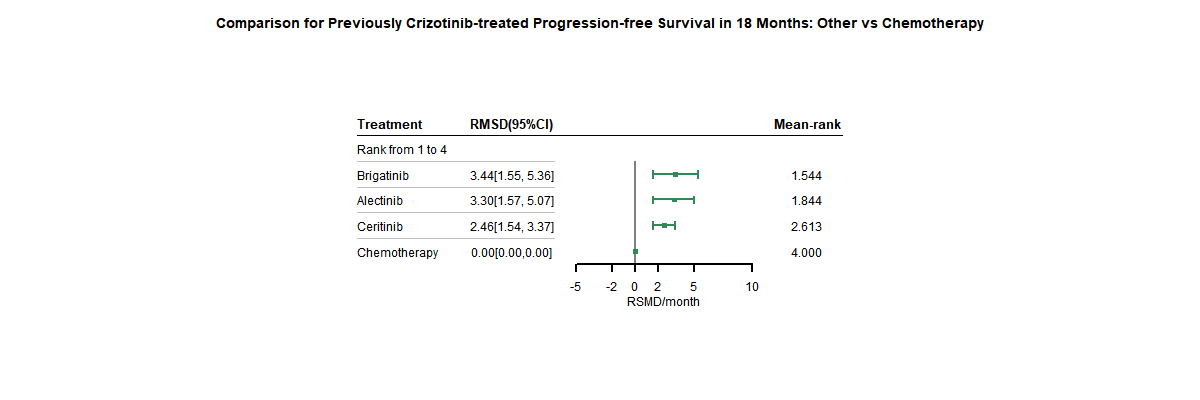
**


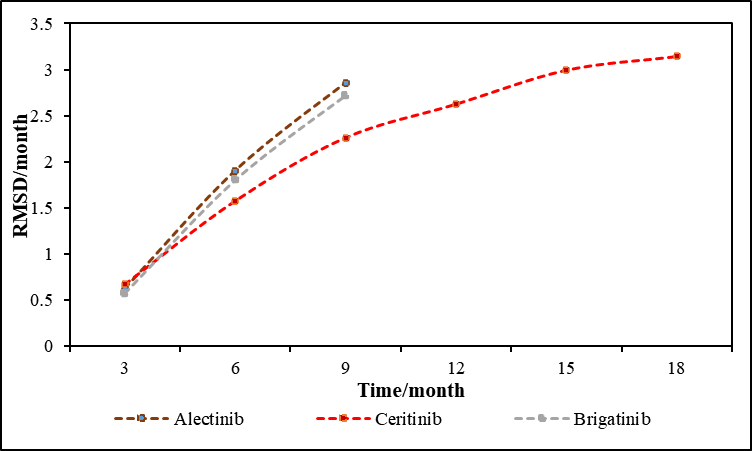


1. **Results from FP model**

Survival curves predicted by FP model


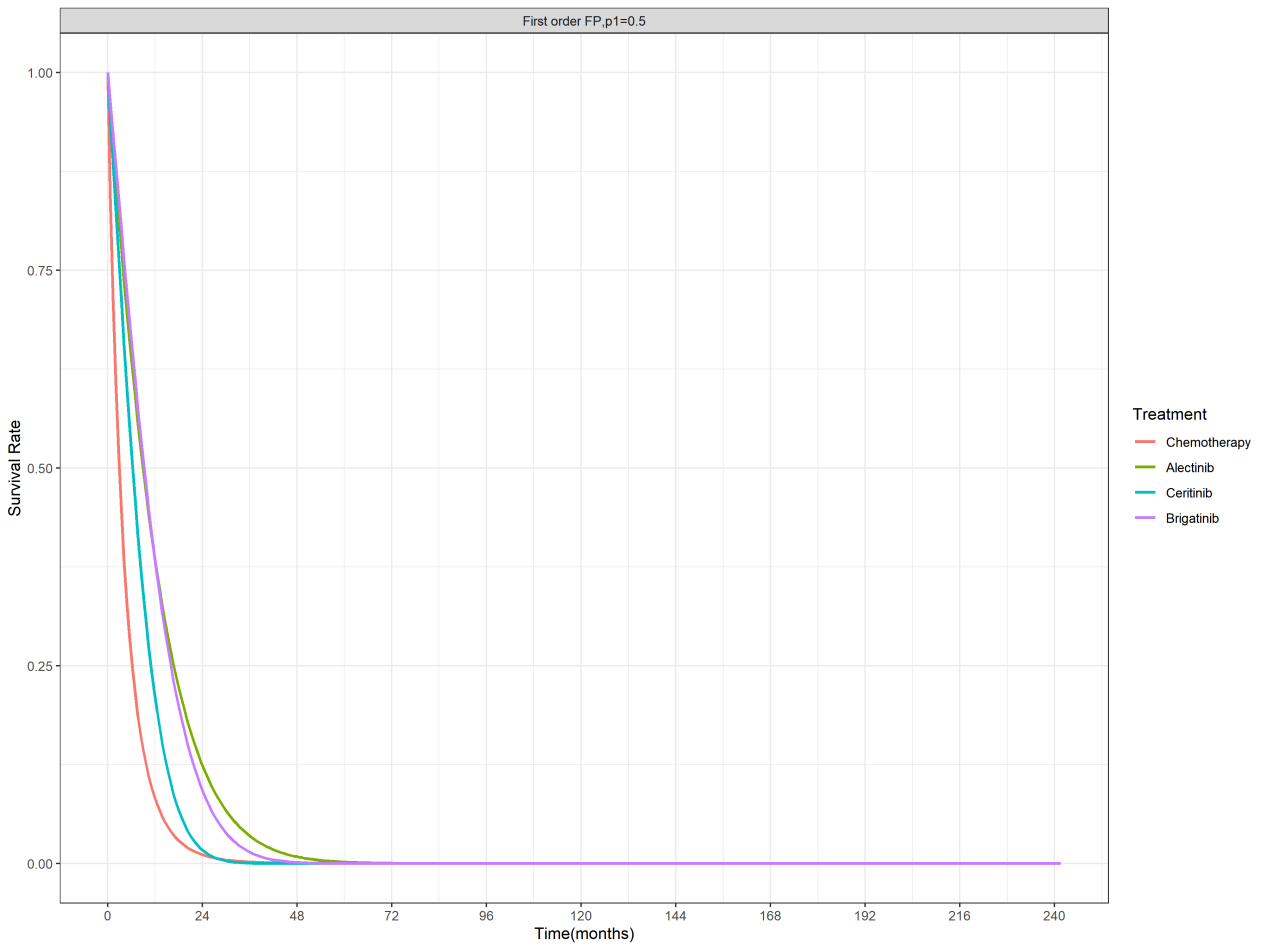


Time-varying HRs (FP model)


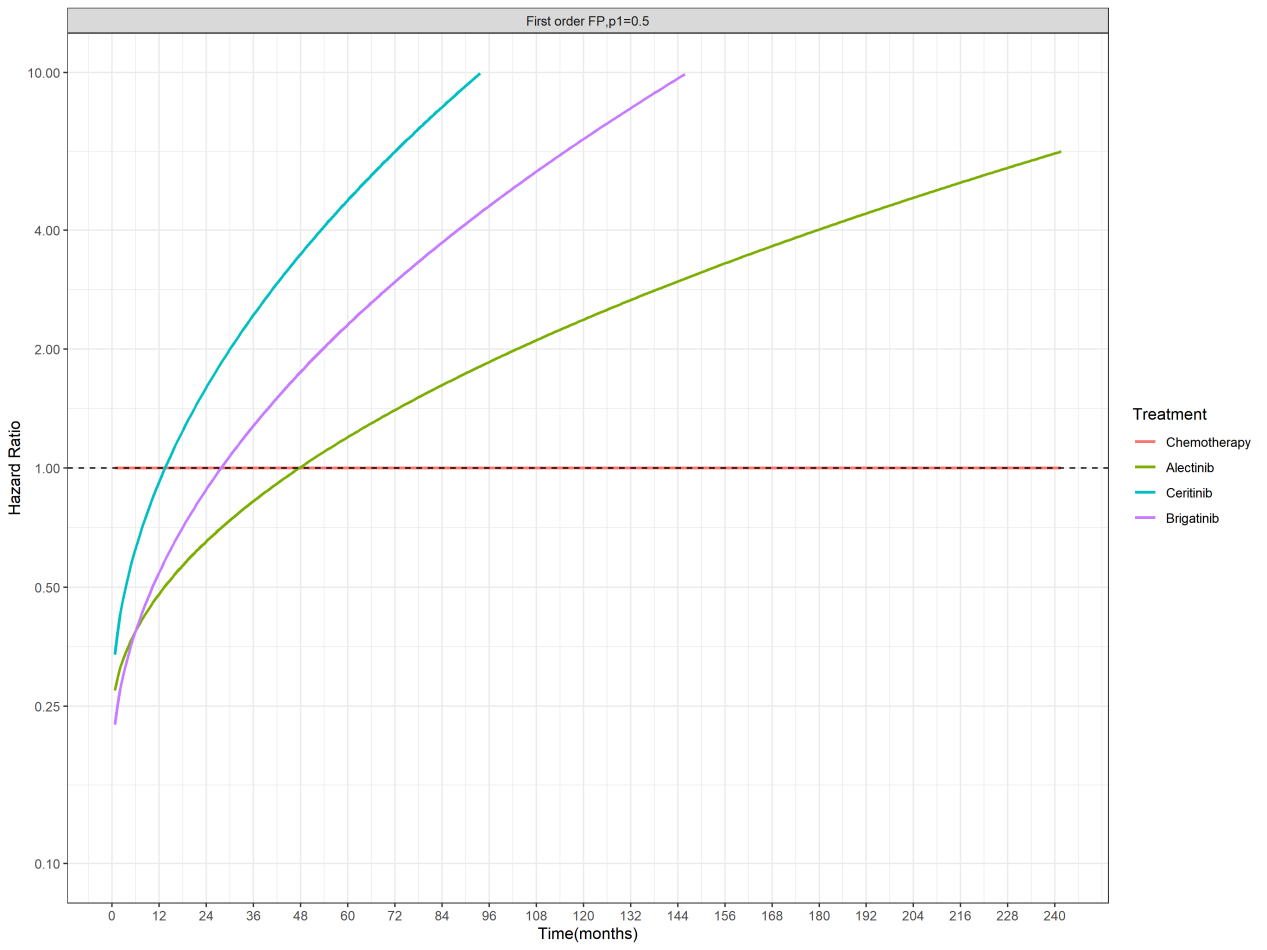


1. **Results from Cox-PH model**

**
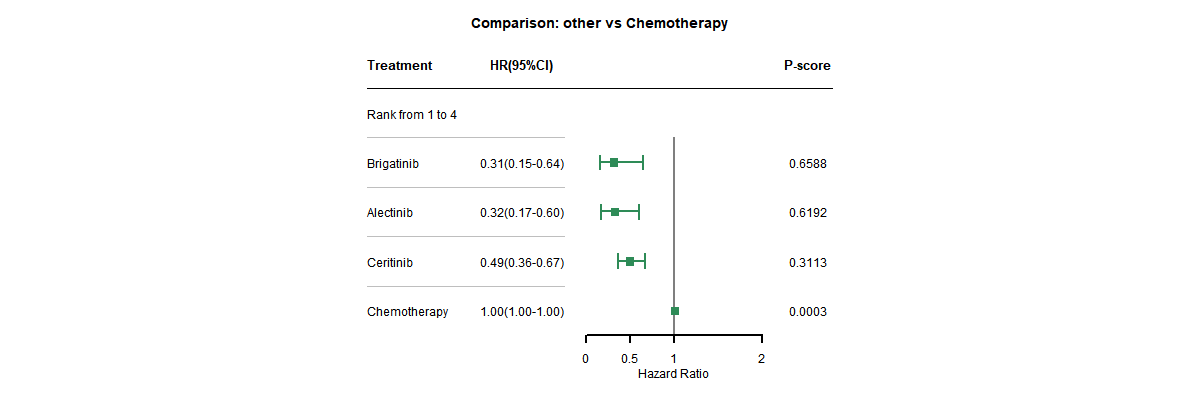
**
